# Parkinson’s disease-related Leucine-rich repeat kinase 2 modulates nuclear morphology and genomic stability in striatal projection neurons during aging

**DOI:** 10.1186/s13024-020-00360-0

**Published:** 2020-02-19

**Authors:** Xi Chen, Chengsong Xie, Wotu Tian, Lixin Sun, Zheng Wang, Sarah Hawes, Lisa Chang, Justin Kung, Jinhui Ding, Shengdi Chen, Weidong Le, Huaibin Cai

**Affiliations:** 1 Transgenic Section, Laboratory of Neurogenetics, National Institute on Aging, National Institutes of Health, Building 35, Room 1A112, MSC 3707, 35 Convent Drive, Bethesda, MD 20892–3707 USA; 2 Clinical Research Center on Neurological Diseases, the First Affiliated Hospital, Dalian Medical University, Dalian, 116011 People’s Republic of China; 3 Department of Neurology, Ruijin Hospital Affiliated to Shanghai Jiao Tong University School of Medicine, Shanghai, 20025 China; 4 Computational Biology Group, Laboratory of Neurogenetics, National Institute on Aging, National Institutes of Health, Bethesda, MD 20892 USA

**Keywords:** Parkinson’s disease, LRRK2, Striatal spiny projection neuron, Nuclear hypertrophy, Nuclear invagination, Nuclear DNA damage, Dendritic hypotrophy, G2019S, R1441C, GABAA, Excitability, And aging

## Abstract

**Background:**

Multiple missense mutations in Leucine-rich repeat kinase 2 (LRRK2) are associated with familial forms of late onset Parkinson’s disease (PD), the most common age-related movement disorder. The dysfunction of dopamine transmission contributes to PD-related motor symptoms. Interestingly, LRRK2 is more abundant in the dopaminoceptive striatal spiny projection neurons (SPNs) compared to the dopamine-producing nigrostriatal dopaminergic neurons. Aging is the most important risk factor for PD and other neurodegenerative diseases. However, whether LRRK2 modulates the aging of SPNs remains to be determined.

**Methods:**

We conducted RNA-sequencing (RNA-seq) analyses of striatal tissues isolated from *Lrrk2* knockout (*Lrrk2*^−/−^) and control (*Lrrk2*^+/+^) mice at 2 and 12 months of age. We examined SPN nuclear DNA damage and epigenetic modifications; SPN nuclear, cell body and dendritic morphology; and the locomotion and motor skill learning of *Lrrk2*^+/+^ and *Lrrk2*^−/−^ mice from 2 to 24 months of age. Considering the strength of cell cultures for future mechanistic studies, we also performed preliminary studies in primary cultured SPNs derived from the *Lrrk2*^+/+^ and *Lrrk2*^−/−^ mice as well as the PD-related *Lrrk2* G2019S and R1441C mutant mice.

**Results:**

*Lrrk2*-deficiency accelerated nuclear hypertrophy and induced dendritic atrophy, soma hypertrophy and nuclear invagination in SPNs during aging. Additionally, increased nuclear DNA damage and abnormal histone methylations were also observed in aged *Lrrk2*^−/−^ striatal neurons, together with alterations of molecular pathways involved in regulating neuronal excitability, genome stability and protein homeostasis. Furthermore, both the PD-related *Lrrk2* G2019S mutant and LRRK2 kinase inhibitors caused nuclear hypertrophy, while the *Lrrk2 R1441C* mutant and γ-Aminobutyric acid type A receptor (GABA-AR) inhibitors promoted nuclear invagination in the cultured SPNs. On the other hand, inhibition of neuron excitability prevented the formation of nuclear invagination in the cultured *Lrrk2*^−/−^ and R1441C SPNs.

**Conclusions:**

Our findings support an important physiological function of LRRK2 in maintaining nuclear structure integrity and genomic stability during the normal aging process, suggesting that PD-related LRRK2 mutations may cause the deterioration of neuronal structures through accelerating the aging process.

## Background

Multiple missense mutations in *LRRK2* gene have been linked to the autosomal dominant familial forms of PD [1, 2]. *LRRK2* gene locus has also been associated with sporadic PD [3, 4]. Extensive studies have been focusing on understanding the pathogenic mechanisms of PD-related *LRRK2* mutations [5–10]. Notably, the genetic burden of LRRK2 variants appears to correlate with the age at onset of disease [11] and the penetrance of *LRRK2* mutations is increased with age [4]. These studies support a potential pathogenic interplay between aging and disease-related genetic mutations in determining the onset and progression of the disease. However, despite aging being the most significant risk factor for PD and other neurodegenerative diseases [2, 12, 13], whether LRRK2 regulates normal neuronal aging is unknown.

Aging research has experienced an unprecedented advance over recent years, particularly with the discovery that the rate of aging is controlled, at least to some extent, by genetic pathways and biochemical processes [14]. Genomic instability, epigenetic alterations and loss of proteostasis are among the key aging hallmarks [14]. The alterations of nuclear structures have been indicated in the neuronal aging [15–17]. Irregular shapes of nuclei had been reported in the neural precursor cells and hippocampal neurons of PD patients carrying PD-related *LRRK2* G2019S mutation [18, 19] and in the midbrain dopaminergic neurons of transgenic mice with ectopic expression of PD-related *LRRK2* R1441C mutation [20]. However, it is unclear whether these nuclear morphological changes are the result of *LRRK2* malfunction, aging or a combination of both, due to the absence of longitudinal in-vivo studies.

*LRRK2* is more abundantly expressed by the neurons in forebrain regions, such as cerebral cortical neurons and SPNs compared to dopaminergic neurons in midbrain areas [21–23]. Here, we performed longitudinal studies to systematically examine morphological, genetic, and functional abnormalities of SPNs in young and aged *Lrrk2*^−/−^ mice and revealed a critical physiological function of LRRK2 in maintaining nuclear morphology and genome integrity during the aging process.

## Methods and materials

### Animals

*Lrrk2*^−/−^ [24], *Lrrk2* G2019S knock-in (KI) [25], *Lrrk2* R1441C [26] KI mice were created as described previously and maintained in the C57BL/6 J strain background. Two to five mice were housed in each cage and in a 12 h light/dark cycle and fed regular diet ad libitum. All mouse work followed the guidelines approved by the Institutional Animal Care and Use Committee of National Institute on Aging, NIH.

### Primary neuronal cell cultures

Primary neuronal cultures from the striatum of postnatal day 0 (P0) pups were prepared as described previously [24]. In brief, neurons were dissociated by papain buffer (Sigma), and were then placed in the poly-D-lysine coated slides (BD) or plates in Basal Eagle Medium (Sigma). Arabinosylcytosine (Sigma) was used to inhibit glial cell growth. Tetrodotoxin (TTX, Sigma), Bicuculline (Sigma) and LRRK2 kinase inhibitor MLi-2 (Tocris Bioscience, Bristol, UK) were added directly to the medium of striatal neurons from the stock solutions.

### Electron microscope

Mice were transcardially perfused with 2% glutaraldehyde, 2% paraformaldehyde (PFA), in 150 mM cacodylate (CB) buffer (pH 7.4). The brain was dissected out and post-fixed in the same fixative solution for 8 h. Subsequently, the tissues were rinsed in 150 mM CB buffer for 4 h. Tissue sections from the perfused brain were cut on a vibratome (Leica, Germany) at 200 μm thickness and stained for EM. For the neuronal cultures, the samples were fixed with 4% PFA in PBS buffer for 20 min and subsequently rinsed with PBS buffer three times. EM tissue staining was performed in the Electron Microscopy Core (NHLBI, NIH). In brief, the brain slices and cell cultures were post-fixed with 1.5% potassium ferrocyanide and 1% osmium tetroxide, then with 1% osmium tetroxide alone, and finally in 1% aqueous uranyl formate (UF). The UF solution was prepared by dissolving UF salt to 1% w/v in boiling water and then titrating in 5 M NaOH until the yellow color became deeper and the final pH was measured about 4.5 by litmus test. The samples were dehydrated with increasing concentrations of alcohol, embedded in Durcupan resin, and hardened at 65 °C for 24 h. The region for thin sectioning was cut from the embedded sections and mounted onto a blank resin block. Silver-gray sections were cut at 50 nm thickness with a diamond knife and mounted onto single slot copper grids with a pioloform support film, stained with UF and imaged at 80 kV in a TEM (JEOL Ltd., Akishima, Tokyo, Japan).

### Immunofluorescence staining

Mice were anesthetized with ketamine and then transcardially perfused with 4% PFA/PBS solution as described previously [27]. Brains were isolated and post fixed in 4% PFA overnight, and then submerged in 30% sucrose for 24 h at 4 °C for later sectioning. Series of 40 μm (except for the dendritic morphology experiments) or 60 μm (only for the dendritic morphology experiments) sections were collected using a cryostat (Leica Biosystems). Sections were blocked in 10% normal donkey serum, 1% bovine serum albumin, 0.3% Triton X-100, PBS solution for overnight at 4 °C. The sections were then incubated with the primary antibodies over one to two nights at 4 °C. Sections were then washed three times in PBS before being incubated in the secondary antibody solutions with Alexa 488- or Alexa Fluor 546-, or Alexa Fluor 633-conjugated secondary antibodies (1:500, Invitrogen) at 4 °C for overnight. Following three washes in PBS, sections were mounted onto subbed slides, and coverslipped with mounting media (ProLong® Gold Antifade Mountant, Life technology). The stained sections were imaged using a laser scanning confocal microscope (LSM 780 or 880; Zeiss). The paired images in the figures were collected at the same gain and offset settings.

Cultured neurons were fixed in 4% PFA/PBS solution as described previously [24]. Briefly, they were permeabilized with 1% Triton-X-100 and incubated with 10% donkey serum for 1 h to block unspecific binding at room temperature and incubated overnight with the primary antibodies at 4 °C. Then the coverslips were washed three times in PBS before being incubated in the secondary antibody solutions with Alexa 488- or Alexa Fluor 546-, or Alexa Fluor 633-conjugated secondary antibodies (1:500, Invitrogen) at 4 °C for overnight. Fluorescent images were captured using a laser scanning confocal microscope (LSM 780 or 880; Zeiss). The paired images in the figures were collected at the same gain and offset settings.

### Image analysis

For the quantitative assessment of nuclear and soma size in the striatum, the patches and matrix compartments in the dorsal lateral striatum (DLS) were chosen randomly and imaged by 40 × oil immersion objective lens. The z-stacked images were taken and exported to ImageJ (NIH) for imaging analyses. When performing the analyses, the images were converted to an 8-bit color scale using ImageJ. The boundary of selected patch and adjacent matrix compartments was first defined by Freehand selection tools. Then the areas of nucleus and their soma in distinct compartments were outlined manually. The presented data included about 100 neurons per group (50 for patch and 50 for matrix) sampled from at least three independent experiments. The counters were blinded to the genotypes of the samples.

For the quantitative assessment of nuclear and soma size in the neuronal cultures, microscopic fields were chosen randomly and imaged by the 63 × oil immersion objective lens. The z-stacked images were taken and exported to ImageJ for imaging analyses. When performing the analyses, images were converted to an 8-bit color scale using ImageJ. The areas of nucleus and soma were outlined manually. The presented data included about 50 neurons per group from at least three independent experiments. The counters were blinded to the genotypes of the samples.

### RNA isolation and preparation

RNA was prepared as previously described [27]. Briefly, mice were anesthetized with CO_2_ followed by decapitation. The striatal regions were rapidly dissected and frozen in liquid nitrogen and stored at − 80 °C until further processing. RNA extraction from the frozen samples was performed using the QIAzol Lysis Reagent and RNeasy Lipid Tissue Mini Kit based on the manufacturer’s instruction (Qiagen).

### RNA sequencing and data analysis

The extracted RNA was quantified using a NanoDrop spectrophotometer (ThermoFisher) and the RNA integrity was measured using RNA Nano Chips and Agilent 2100 Bioanalyzer (Agilent). The cDNA libraries were generated from purified mRNA using TruSeq RNA Sample Preparation Kits (v2, Illumina) according to the manufacturer’s instructions. The samples were sequenced with Illumina HiSeq 2000 (BGI, Cambridge, MA).

The standard Illumina pipeline was used to generate Fastq files. The Ensembl annotated transcript abundance was quantified using Salmon in a non-alignment-based mode, and gene level counts were estimated using Tximport package (Bioconductor). For the differential gene expression analysis, we utilized the DESeq2 package (Bioconductor). Prior to calculating test statistics for each gene, we filtered out the lowest 25% of genes based on their mean counts. The counts for the resulting genes were then normalized using a variance-stabilizing transformation and the two groups were compared using a generalized linear model in DESeq2. *P* values were adjusted using the Benjamini-Hochberg method.

The list of genes with significantly altered expression (Benjamini-Hochberg adjusted *p* < 0.05) was run by DAVID enrichment analysis. Data were plotted using either R (http://www.rstudio.com/) or Excel.

### Protein extraction and Western blot

For the total protein lysates, the striatum tissues were homogenized using glass homogenizer with 10 volumes of RIPA buffer plus protease and phosphatase inhibitor cocktails and then the mixture was added with 4 × protein loading buffer (Invitrogen) with shaking and heating for 3 min. After that, the samples were centrifuged at 13, 000 rpm for 10 min at 4 °C and the supernatant was preserved.

The prepared protein extracts were size fractioned by 4 to 12% NuPAGE Bis-Tris gel electrophoresis (Invitrogen) using MES running buffer (Invitrogen). After transfer to the nitrocellulose membranes using Transfer Cell (Bio-Rad), the membranes were blocked with Odyssey Blocking Buffer (LI-COR) and probed overnight with the appropriate dilutions of the primary antibodies. Incubation with the IRDye-labeled secondary antibodies (LI-COR, 1:10000) was performed for 1 h at room temperature. The protein bands of interest were visualized with Odyssey CLx Infrared Imaging Studio. The band intensity was quantified using ImageJ.

### Stereology

According to the mouse brain in stereotaxic coordinates, a series of coronal sections across the striatum (40 μm per section, every eight section from bregma 1.70 mm to − 0.94 mm) were chosen and processed for DARPP-32 (CST, cat#2306) and DAPI staining, finally visualized using a laser scanning confocal microscope (LSM 780, Zeiss). We examined 11 sections per brain. The images were captured as a single optic layer under 10 × objective lens. The volume of dorsal striatum, ventral striatum and forebrain was first outlined based on the mouse brain atlas [28] and then reconstructed in 3D model using Stereo Investigator 10 (MBF Bioscience). After done with the quantification of volume, the outline of the dorsal striatum was considered as the boundary for counting the number of DARPP-32–positive neurons inside. The number of DARPP-32–positive neurons was assessed using the fractionator function of Stereo Investigator 10 (MBF Bioscience). The sampling scheme was designed to have coefficient of error (CE) of less than or equal to 5% in order to get reliable results. To achieve suitable CE, normally 11 serial sections, with a total of 320 counting frames were assessed. The final parameters of these studies were as follows: grid size, 350 × 300 μm; and frame size, 100 × 100 μm. Five mice were used per group. Counters were blinded to the genotypes of the samples.

### Stereotaxic viral injection

The stereotaxic AAV injections (AAV-hSyn1-eGFP, Penn Vector Core) were conducted on 2- and 11-month-old *Lrrk2*^+/+^ and *Lrrk2*^−/−^ mice. Before surgery, mice were deeply anesthetized by intraperitoneal injection of ketamine (100 mg/kg)/xylazine (10 mg/kg) solution. To achieve sparse labeling, 1.55 × 10^11^ viral particles with a total volume of 500 nl were injected into dorsal striatum (coordinates used, AP: 0.98 mm, ML: ±2.2 mm from bregma, DV: − 3.0 mm from exposed dura mater). Virus solution was injected at an infusion rate of 100 nl/min and the needle was withdrawn 10 min after the end of injection. Following virus injection, the scalp was sutured, and the mice were returned to their home cages. The virus-injected mice were used for experiment at least 4 weeks after the virus infusion.

### Stereology for neuronal tracing

Based on the previous study [29], the AAV-infused mouse brains were sectioned at 60 μm of thickness. The sections were stained with GFP antibody (Abcam, cat#ab6662) and CTIP2 antibody (Abcam, cat#ab18465). Afterwards, the stained sections were imaged using a laser scanning confocal microscope (LSM 780 or 880, Zeiss) under 40 × objective lens. The SPNs were identified based on the positive staining of CTIP2. Neuronal structure reconstruction and Sholl analysis were performed with Neurolucida 360 software (MBF Bioscience).

### Motor behavioral tests

Rotarod motor skill learning test. As described previously [30], mice were placed onto a rotating rod with auto acceleration from 0 to 40 rpm in 5 min (Panlab). The length of time the mouse stayed on the rotating rod was recorded across 10 trials. Such experiments were performed on 6 continuous days [30].

Open-field velocity test by video-tracking. As described previously [30], video recording of each mouse was conducted using a LifeCam cinema webcam. For each trial, a white arena floor allowed for further analyses using EthoVision XT software package (Noldus IT), which detects the subjects against a monochrome background. All video files were analyzed using EthoVision XT software, and for each video, a still frame of empty arena was used for calibration. The velocity was calculated and exported from this software.

### Antibodies

**Table:** 

LaminB1	Santa Cruz Biotechnology	sc-30,264
LaminB1 (1:1000 dilution)	Santa Cruz Biotechnology	sc-374,015
CTIP2 (1:200 dilution)	Abcam	ab18465
Nup98 (1:500 dilution)	CST	2598
MOR1 (1:3000 dilution)	Immunostar	24,216
Calbindin (1:500 dilution)	CST	13,176
βIII tubulin (1:3000 dilution)	Abcam	ab18207
LRRK2 (1:1000 dilution)	Abcam	ab133474
actin (1:3000 dilution)	Sigma	A3853
GFP (1:1000 dilution)	CST	2956
GFP (1:1000 dilution)	Life technology	G10362
GFP (1:1000 dilution)	Abcam	ab6662
Histone H3 (1:3000 dilution)	CST	4499
Histone H2A (1:1000 dilution)	CST	7631
Phospho-Histone H2A.X (1:1000 dilution)	CST	2577
Histone H3K9me2 (1:1000 dilution)	Abcam	ab1220
TH (1:2000 dilution)	Immunostar	22,941
TH (1:2000 dilution)	Pel-Freez	P40101–150
MAP2 (1:1000 dilution)	Abcam	ab5392
DAT (1:1000 dilution)	Millipore	MAB369
Darpp-32 (1:1000 dilution)	CST	2306

### Statistics

Graph Pad Prism 7 and R were used for statistical analysis. The data were collected and processed randomly. No statistical methods were used to predetermine sample size, but our sample sizes are similar to those reported in previous publications. The statistical significance was determined using Student’s t-test, 1way ANOVA with Sidak’s multiple comparisons, 2way ANOVA with Sidak’s multiple comparisons test, conditional logistic regression, and multiple t-test with Benjamini and Hochberg test.

## Results

### Differential alteration of gene expression in *Lrrk2*^−/−^ striatal neurons during aging

**Fig. 1:**
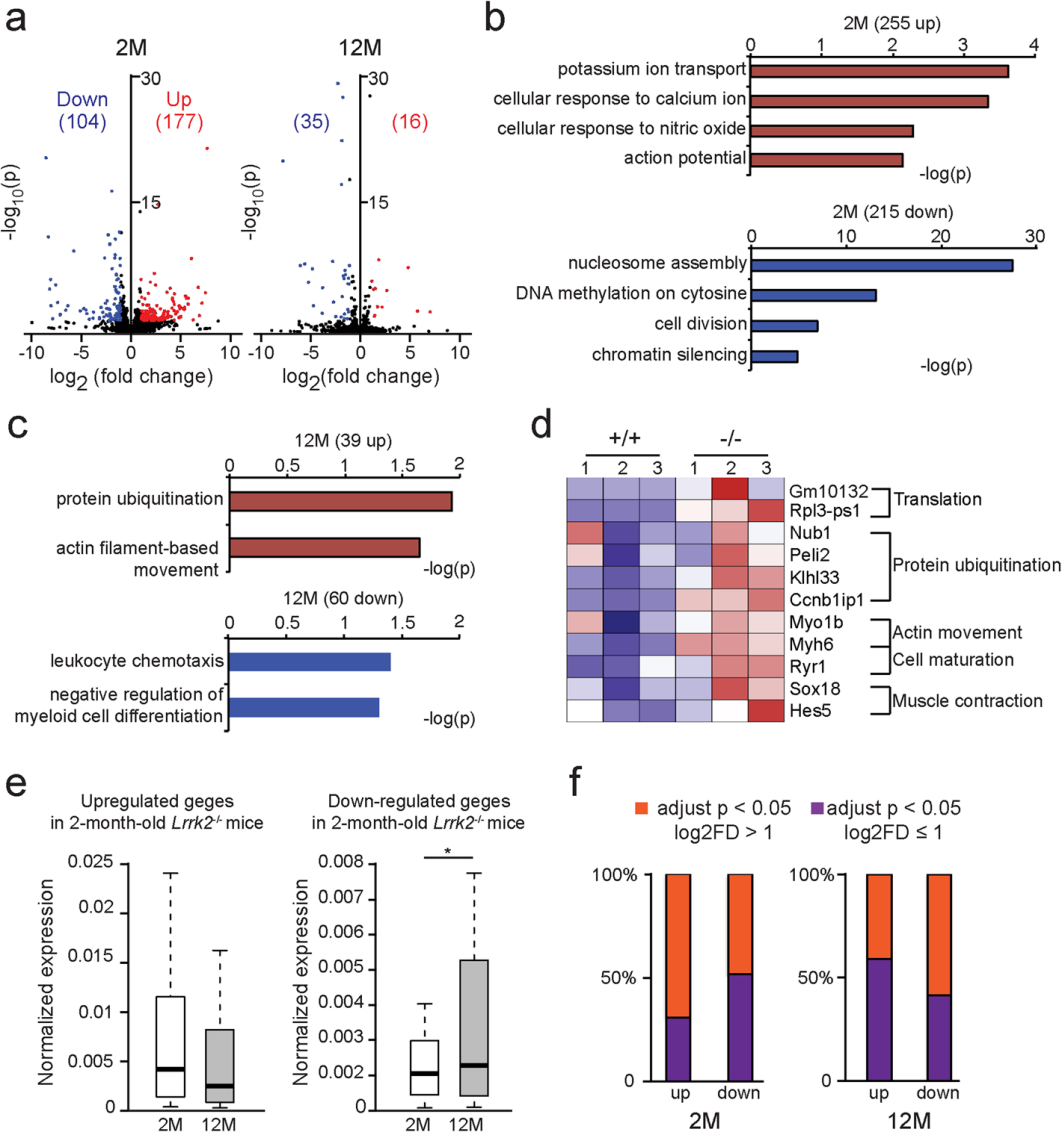
Alteration of gene expression in *Lrrk2*^−/−^ striatal neurons during aging. **a** The volcano plots of RNA-seq data collected from the dorsal striatum of *Lrrk2*^+/+^ and *Lrrk2*^−/−^ mice at 2 months (*n* = 3 and 4 for the *Lrrk2*^+/+^ and *Lrrk2*^−/−^ mice, respectively) and 12 months (n = 3 mice per genotype) of age. Adjusted *p* < 0.05, log_2_FD > 1. **b**-**d** GO enrichment analysis for the data collected from the *Lrrk2*^+/+^ and *Lrrk2*^−/−^ mice at 2 months (**b**) and 12 months of age **(c)** using DAVID. Adjusted *p* < 0.05. Supervised clustering for the data collected from the 12-month-old *Lrrk2*^+/+^ and *Lrrk2*^−/−^ mice (**d**). **e** The upregulated genes of the 2-month-old *Lrrk2*^−/−^ mice showed a decreased trend when calculated again at 12 months of age. In contrast, the downregulated genes display a significant enhancement during aging. Paired t-test, ^*^p = 0.0131. **f** The upregulated genes are more common in the 2-month-old *Lrrk2*^−/−^ samples. The downregulated genes occur more often in the 12-month-old *Lrrk2*^−/−^ samples

Since LRRK2 is highly enriched in SPNs [21], we performed gene expression analysis of the dorsal striatal tissues isolated from *Lrrk2*^+/+^ and *Lrrk2*^−/−^ mice at 2 and 12 months of age. In contrast to previous microarray gene expression studies [31, 32], we performed whole-genome RNA-seq analyses and Salmon indexing subsequently [33]. Overall, we found more dynamic changes in gene expression between the 2-month-old *Lrrk2*^+/+^ and *Lrrk2*^−/−^ mice compared to the 12-month-old pairs (Fig. 1a and Additional file 1: Table S1). Gene ontology (GO) analysis of genes affected by *Lrrk2*-deficiency indicate that in the 2-month-old *Lrrk2*^−/−^ mice, the upregulated genes are mainly involved in potassium ion (K^+^) transport, cellular response to calcium ion (Ca^2+^) and action potential pathway (Fig. 1b). On the other hand, the downregulated genes are extensively linked to nucleosome assemble pathways (Fig. 1b). In contrast to the gene expression profile at 2 months of age, in the 12-month-old *Lrrk2*^−/−^ mice, the upregulated genes are more engaged in protein ubiquitination and actin movement, which were further elucidated by supervised clustering analysis (Fig. 1c, d and Additional file 2: Table S2). Meanwhile, the molecular pathways involved in leukocyte chemotaxis and myeloid cell differentiation were among those most downregulated in the 12-month-old *Lrrk2*^−/−^ mice (Fig. 1c). Furthermore, while the genes upregulated in the 2-month-old *Lrrk2*^−/−^ mice showed a tendency toward decreased expression at 12 months of age, the genes downregulated in young *Lrrk2*^−/−^ mice exhibited an opposite, increased expression pattern during aging (Fig. 1e). Additionally, a higher percentage of the upregulated genes displayed more robust changes (> 2 fold) compared to the downregulated genes in 2-month-old *Lrrk2*^−/−^ mice (Fig. 1f). By contrast, a higher percentage of the downregulated genes displayed more substantial changes (> 2 fold) compared to the upregulated genes in 12-month-old *Lrrk2*^−/−^ mice (Fig. 1f). These gene expression analyses reveal a dynamic interplay between *Lrrk2*-deficiency and aging that differentially affects gene expression in distinct molecular pathways. The alteration of nuclear assemble pathway in *Lrrk2*^−/−^ mice implies an important physiological function of LRRK2 in regulating nuclear structure during aging.

### *Lrrk2*-deficiency disturbs genomic stability during aging

**Fig. 2:**
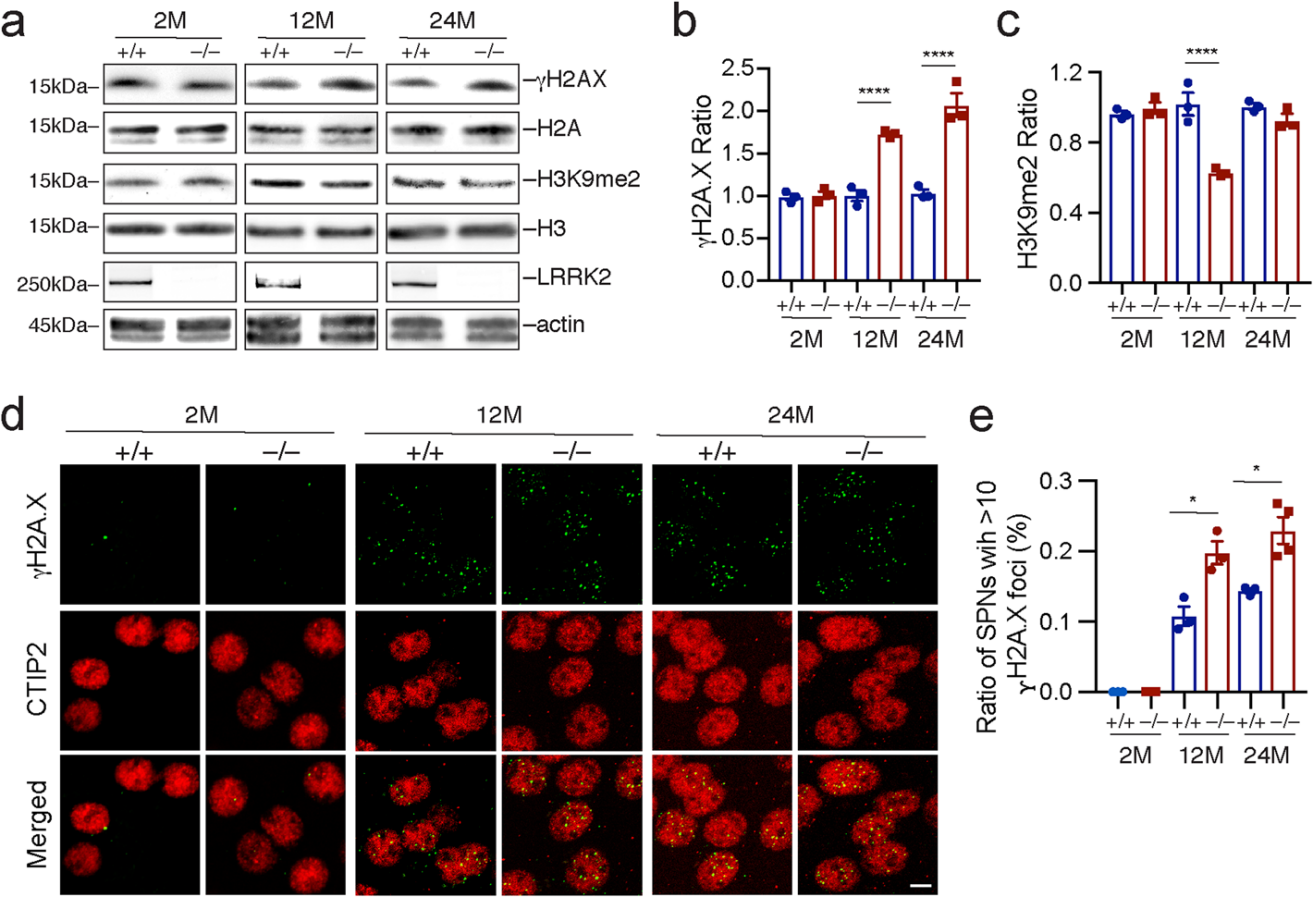
*Lrrk2*-deficiency disturbs genomic stability and epigenetic modification during aging. **a** The expression levels of γH2A.X and H3K9me2 were detected by western blot analysis from the striatal samples of *Lrrk2*^+/+^ and *Lrrk2*^−/−^ mice at 2, 12 and 24 months of age. **b, c** The ratios of γH2A.X (**b**) and H3K9me2 (**c**) against H2A and H3, respectively. *N* = 3 per genotype and per time point. Data were presented as mean ± SEM. 2way ANOVA analysis with Sidak’s multiple comparisons test of γH2A.X, ^****^p < 0.0001 (*Lrrk2*^+/+^ vs. *Lrrk2*^−/−^ at 12 months of age), ^****^p < 0.0001 (*Lrrk2*^+/+^ vs. *Lrrk2*^−/−^ at 24 months of age). 2way ANOVA analysis with Sidak’s multiple comparisons test of H3K9me2 expressions, ^****^p < 0.0001. **d** Co-staining of γH2A.X and CTIP2 in the striatal sections of 2-, 12-, and 24-month-old *Lrrk2*^+/+^ and *Lrrk2*^−/−^ mice. Scale bar, 5 μm. **e** The ratios of SPNs with 10 or more γH2A.X-positive foci in the nuclei. N = 3 or 4 mice per genotype, 400 neurons per animal. Data were presented as mean ± SEM. Unpaired t-test, ^*^p = 0.0126 (12 M, +/+ vs. −/−), ^*^p = 0.0132 (24 M, +/+ vs. −/−)

Since nuclear assembly pathways were altered during aging, we examined the markers for DNA damage and repair, as well as epigenetic modifications in the striatal tissues of *Lrrk2*^+/+^ and *Lrrk2*^−/−^ mice at 2, 12, and 24 months of age. We found a substantial increase of γH2AX, a marker for DNA double-strand breaks and damage [34] in the striatal tissues of 12- and 24-month-old *Lrrk2*^−/−^ mice compared to the 2-month-old ones (Fig. 2a, b). By contrast, no significant changes of γH2AX ratios were found in the hippocampal tissues of 12-month-old *Lrrk2*^−/−^ mice (*n* = 4) compared to the age-matched controls (*n* = 3) (un-paired t test, *p* = 0.96). In addition, we detected a marked reduction of histone methylation in H3K9me2, an epigenetic marker for heterochromatin structures indicative of transcriptional suppression [35], only in the striatal tissues of 12-month-old *Lrrk2*^−/−^ mice (Fig. 2a, c).

Immunostaining further revealed a substantial increase of the percentage of SPNs with 10 or more γH2AX-positive foci in the nuclei of 12-and 24-month-old *Lrrk2*^−/−^ mice compared to age-matched controls (Fig. 2d, e). There were on average 4.7 and 7.3 γH2A.X-positive foci per nucleus in the SPNs of 12-month-old *Lrrk2*^+/+^ and *Lrrk2*^−/−^ mice, respectively (n = 3 mice per genotype, 200 SPNs per animal; un-paired t test, *p* = 0.005). Given that the average nuclear size was 75.8 and 85.0 μm^2^ for the *Lrrk2*^+/+^ and *Lrrk2*^−/−^ SPNs, respectively, the average numbers of γH2A.X-positive foci when normalized to the nuclear area were 0.06 and 0.09 per μm^2^ in the *Lrrk2*^+/+^ and *Lrrk2*^−/−^ SPNs, respectively. Together, these results suggest an important function of LRRK2 in maintaining genomic stability during neuronal aging.

### *Lrrk2*-deficiency accelerates age-related nuclear hypertrophy

**Fig. 3:**
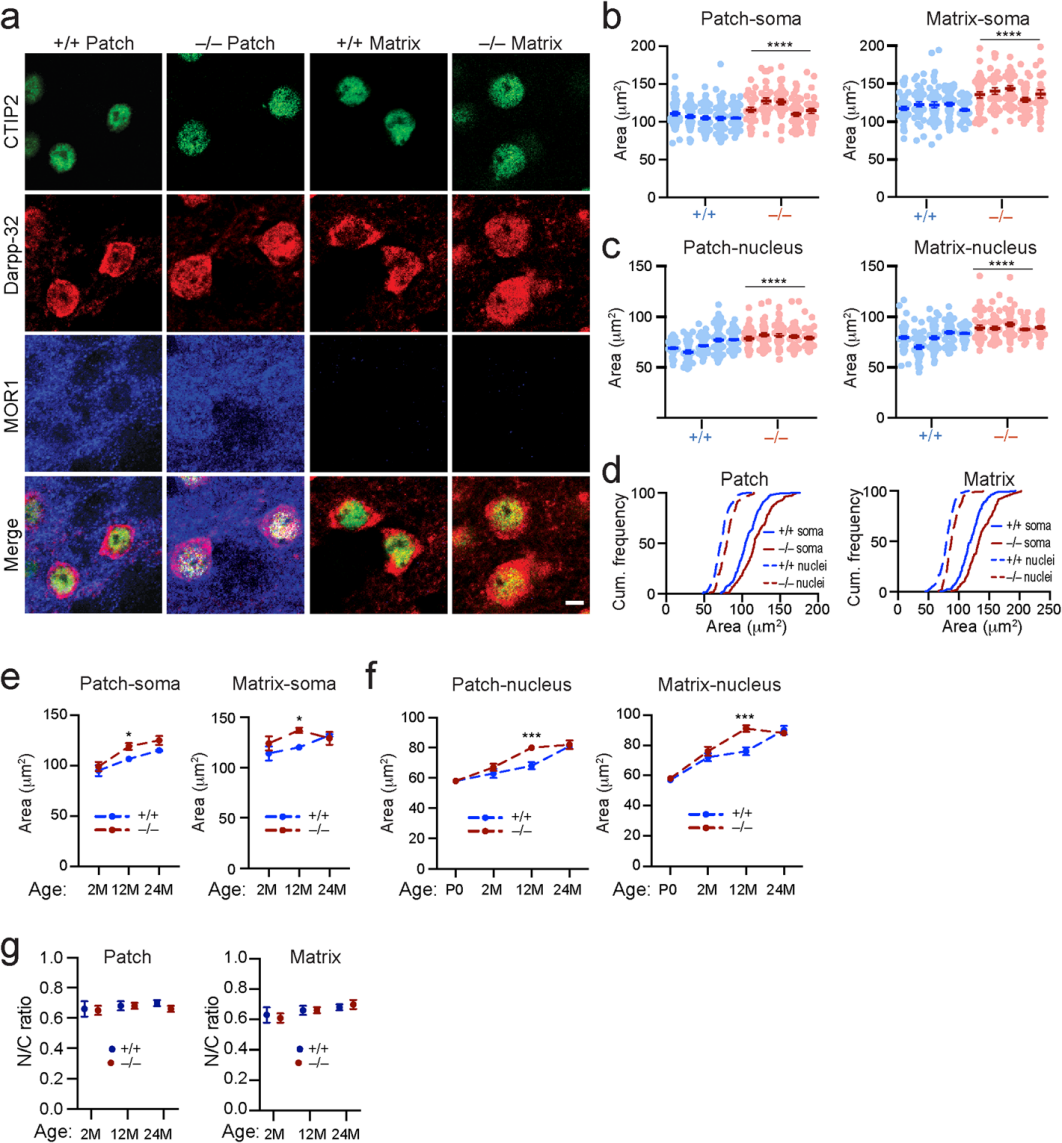
*Lrrk2*-deficiency accelerates nuclear hypertrophy during aging. **a** Co-staining of CTIP2, Darpp-32 and MOR1 in patch and matrix compartments of 12-month-old *Lrrk2*^+/+^ and *Lrrk2*^−/−^ mice. Scale bar, 5 μm. **b**, **c** The soma (**b**) and nucleus (**c**) size of SPNs in the patch and matrix compartments of 12-month-old *Lrrk2*^+/+^ and *Lrrk2*^−/−^ mice. *N* = 5 mice per genotype. 600–100 neurons were counted per animal. Conditional logistic regression test, ^****^p < 0.0001. **d** Cumulative (Cum.) frequency of the soma and nuclear size distribution in the patch and matrix compartments of *Lrrk2*^+/+^ and *Lrrk2*^−/−^ mice. **e** The soma area of SPNs in the patch and matrix compartments of *Lrrk2*^+/+^ and *Lrrk2*^−/−^ mice at 2 (*n* = 3 mice per genotype, 50–80 neurons per animal), 12 (*n* = 5 mice per genotype, 60–100 neurons per animal) and 24 months (*n* = 3 mice per genotype, 60–90 neurons per animal) of age. 2way NOVA analysis with Sidak’s multiple comparisons test, ^*^p = 0.025 (patch soma), ^*^p = 0.0118 (matrix soma). **f** The nuclear size of SPNs in the patch and matrix compartments of *Lrrk2*^+/+^ and *Lrrk2*^−/−^ mice at P0 (*n* = 3 mice per genotype, 60–100 neurons per animal), 2 (*n* = 6 mice per genotype, 50–200 neurons per animal), 12 (*n* = 8 mice per genotype, 60–150 neurons per animal) and 24 months (*n* = 3 per genotype, 60–90 neurons per animal) of age. 2way ANOVA analysis with Sidak’s multiple comparisons test, ^***^p = 0.0005 (patch nuclei), ^***^p = 0.0002 (matrix nuclei). **g** The nuclear size and soma size ratio (N/C ratio) of SPNs in the patch and matrix compartments of *Lrrk2*^+/+^ and *Lrrk2*^−/−^ mice. The number of mice and neurons as indicated in **e** and **f**. 1way ANOVA analysis with Sidak’s multiple comparisons test, no statistically significant difference was identified

When examining the nuclei of the SPNs, we noticed that the nuclear size of SPNs was substantially enlarged in the 12-month-old *Lrrk2*^−/−^ mice compared to the age-matched control animals. Nuclear enlargement or hypertrophy has been associated with disrupted genomic structures [36]. The dorsal striatum can be divided into two complementary compartments named patch (or striosome) and matrix [37]. Since LRRK2 is more abundant in the patch SPNs than the matrix SPNs in rodent brains [22], we quantified the soma and nuclear size of SPNs in both the patch and matrix compartments of 12-month-old *Lrrk2*^−/−^ mice and age-matched littermate controls. The results showed marked enlargement of the soma and nuclear size in the *Lrrk2*^−/−^ SPNs of both compartments (Fig. 3a-d). Moreover, our longitudinal data demonstrate that the soma and nuclear sizes of patch and matrix SPNs were steadily increased in the *Lrrk2*^+/+^ mice from 2 to 24 months of age (Fig. 3e, f). In contrast, a lack of *Lrrk2* abnormally accelerated the enlargement of soma and nuclear sizes in the *Lrrk2*^−/−^ SPNs from 2 to 12 months of age, while no further size increases were observed between 12 and 24-month-old animals (Fig. 3e, f). Despite the alterations in soma and nuclear size, the nucleus to soma ratio (N/C ratio) remained unchanged (Fig. 3g). On the other hand, no apparent alterations of nuclear size were observed in the hippocampal dentate gyrus neurons or nigrostriatal dopaminergic neurons of 12-month-old *Lrrk2*^−/−^ mice compared to the age-matched controls (Additional file 3: Figure S1). Taken collectively, these data demonstrate that LRRK2 is involved in regulating nuclear and soma size during the aging of SPNs.

### *Lrrk2*-deficiency induces nuclear invagination during aging

**Fig. 4:**
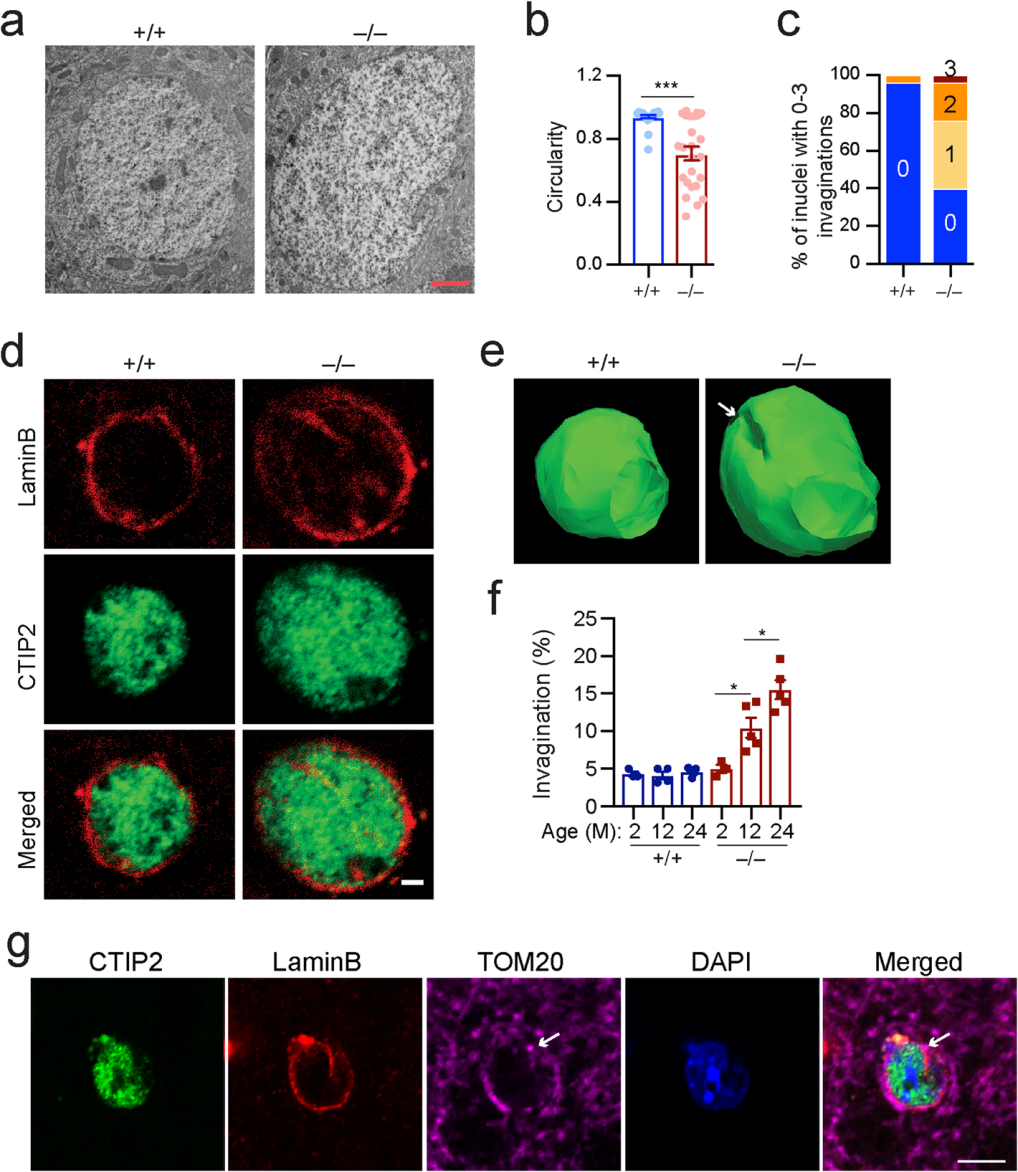
*Lrrk2*-deficiency promotes nuclear invaginations during aging. **a**-**c** Sample EM images from the striatal cells of 12-month-old *Lrrk2*^+/+^ and *Lrrk2*^−/−^ mice (**a**). Scale bar, 2 μm. The nuclear circularity (**b**) and the ratio of nuclei containing 0 to 3 invaginations (**c**) were calculated from the EM images. *N* = 28 neurons for *Lrrk2*^+/+^ mice. *N* = 25 neurons for *Lrrk2*^−/−^ mice. Unpaired t-test of nuclear circularity, ^***^p < 0.0001. **d** Co-staining of Lamin B and CTIP2 in the striatal sections of 12-month-old *Lrrk2*^+/+^ and *Lrrk2*^−/−^ mice. Scale bar, 2 μm. **e** 3D reconstruction of (**d**). The white arrow points to a nuclear invagination. **f** Ratio of SPN nuclei containing ≥1 invagination in *Lrrk2*^+/+^ and *Lrrk2*^−/−^ mice at 2 (*n* = 3 mice per genotype, 30–60 neurons per animal) and 12 (*n* = 4 and 5 for *Lrrk2*^+/+^ and *Lrrk2*^−/−^ mice, respectively; 30–75 neurons per animal), and 24 months of age (*n* = 5 per genotype; 50–70 neurons per animal). 1way ANOVA analysis with multiple comparisons test, ^*^p = 0.0406 (2-month-old *Lrrk2*^−/−^ vs. 12-month-old *Lrrk2*^−/−^ samples), ^*^p = 0.0275 (12-month-old *Lrrk2*^−/−^ samples vs. 24-month-old *Lrrk2*^−/−^ samples). 2way ANONVA analysis with multiple comparisons test, ^***^p = 0.0004 (12-month-old *Lrrk2*^+/+^ vs. 12-month-old *Lrrk2*^−/−^ samples), ^****^p < 0.0001 (24-month-old *Lrrk2*^+/+^ vs. 24-month-old *Lrrk2*^−/−^ samples). **g** Co-staining of CTIP2, LaminB, TOM20, and DAPI in the striatal section of 24-month-old *Lrrk2*^−/−^ mice. The arrow points to the mitochondria inside the mouth of nuclear invagination. Scale bar, 5 μm

We next performed electron microscopy (EM) analyses to further elucidate the morphological alterations of nuclear structure in the SPNs of *Lrrk2*^−/−^ mice. Besides nuclear enlargement, we also found increased nuclear invaginations together with reduced circularity in the SPNs of 12-month-old *Lrrk2*^−/−^ mice (Fig. 4a-c). This increase in invaginations was further confirmed by subsequent immunofluorescent staining, in which the nuclear envelope marker Lamin B [18] and SPN-specific nuclear marker CTIP2 [38] were used (Fig. 4d, e). We found that the percentage of SPNs with nuclear invaginations was around 5% in the *Lrrk2*^+/+^ mice at 2, 12, and 24 months of age (Fig. 4f). By contrast, the percentage of SPNs with nuclear invaginations was progressively increased to 15% in the *Lrrk2*^−/−^ mice from 2 to 24 months of age (Fig. 4f). We additionally stained the striatal sections of 24-month-old *Lrrk2*^+/+^ and *Lrrk2*^−/−^mice using antibodies against the mitochondrial import receptor subunit TOM20 [39]. We randomly selected 12 *Lrrk2*^+/+^ SPNs and 22 *Lrrk2*^−/−^ SPNs containing one or more nuclear invaginations and collected serial Z-stack images under high magnification. With this approach, we were able to visualize mitochondria near nuclear invaginations. Example images from a single optical layer in Fig. 4 show the presence of mitochondria at the mouth as well as inside of the nuclear invagination in one of the *Lrrk2*^−/−^ SPNs (Fig. 4g). We estimate that the percentage of cells with mitochondria near the nuclear invaginations is around 50% in the *Lrrk2*^−/−^ SPNs compared to 8% in the *Lrrk2*^+/+^ SPNs in the striatal sections. In line with previous findings [18–20], these results demonstrate that LRRK2 is required to maintain the integrity of the nuclear envelop structures during the aging process**.**

### *Lrrk2*-deficiency causes striatal atrophy in the aged *Lrrk2*^−/−^ mice

**Fig. 5:**
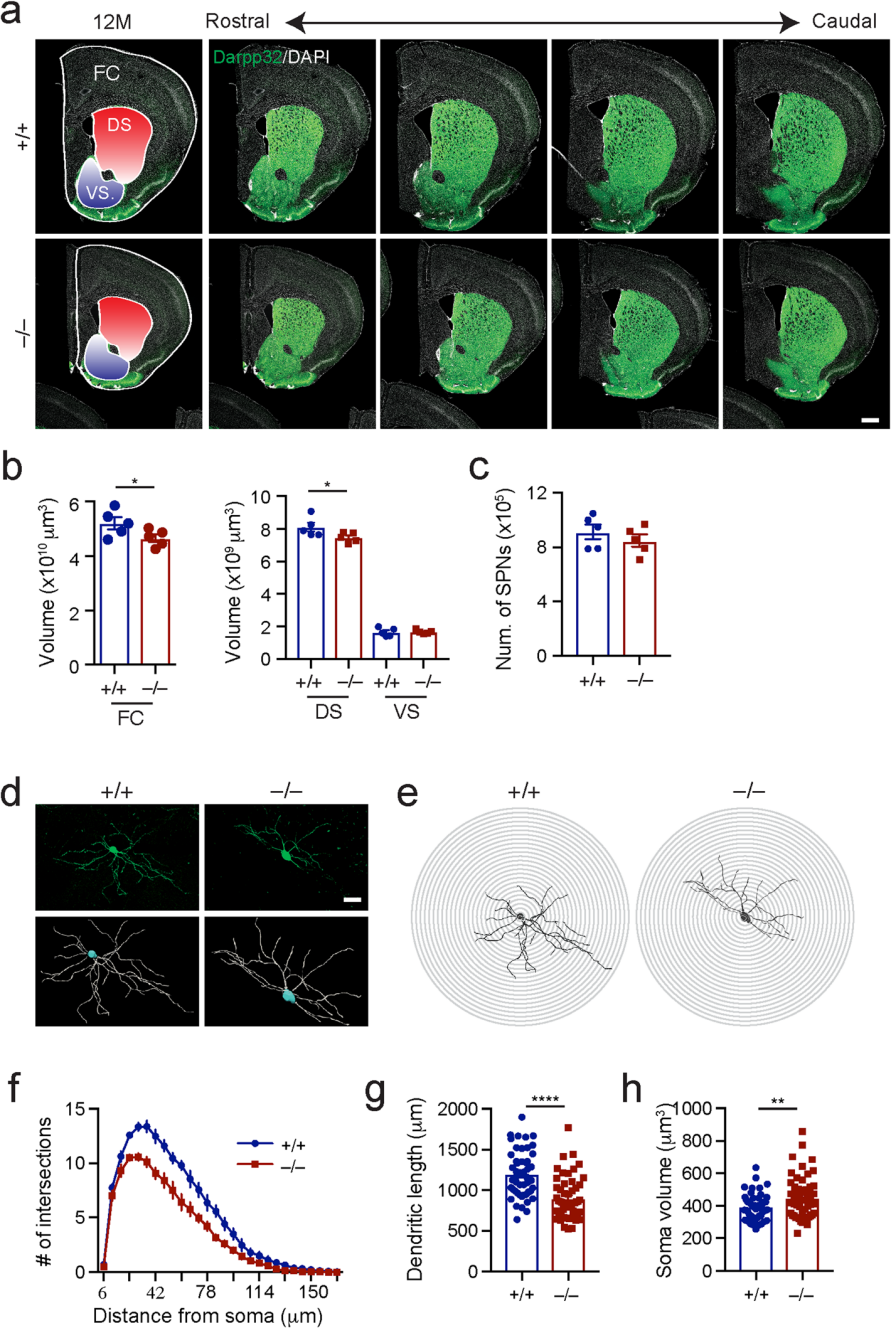
Forebrain atrophy and reduced dendritic complexity in the SPNs in 12-month-old *Lrrk2*^−/−^ mice. **a** Co-staining of Darpp-32 and DAPI in the forebrain coronal sections of 12-month-old *Lrrk2*^+/+^ and *Lrrk2*^−/−^ mice. The dorsal striatum (DS) and ventral striatum (VS) are highlighted with red and blue colors, respectively. Scale bar, 500 μm. **b** The volumes of frontal cerebral cortex (FC), DS and VS. *N* = 5 mice per genotype. Unpaired t-test, ^*^p = 0.034 (FC); ^*^p = 0.037 (DS); *p* = 0.856 (VS). **c** The numbers of SPNs in the DS. N = 5 mice per genotype. Unpaired t-test, *p* = 0.387 of 12-month-old *Lrrk2*^−/−^ mice. **d** The GFP-labeled SPNs (top panel). 3D reconstruction of the top fluorescent images (bottom panel). Scale bar, 50 μm. **e, f** Sholl analysis of dendritic complexity of GFP-labeled SPNs. N = 5 mice per genotype. 5–9 neurons were examined per animal. Benjamin-Hochberg multiple comparison test of dendritic complexity at 24, 30, 36, 42, 48, 54, 60, 72, 84 and 132 μm from soma, q ≤ 0.05. **g** Dendritic length of GFP-labeled SPNs. N = 5 mice per genotype. 5–9 neurons were counted. Unpaired t-test, ^****^p < 0.0001. **h** Soma volume of GFP-labeled SPNs. N = 5 mice per genotype. For each animal, 5–9 neurons were counted. Unpaired t-test, ^**^p = 0.0086

We next examined the volume of striatum and surrounding forebrain regions of 12-month-old littermate *Lrrk2*^+/+^ and *Lrrk2*^−/−^ mice and found a marked reduction of cerebral cortex and dorsal striatum volume in the *Lrrk2*^−/−^ mice (Fig. 5a, b). By contrast, no apparent change of ventral striatum volume was found (Fig. 5a, b). Despite reduced volume, the numbers of SPNs, which account for 95% of neurons in the dorsal striatum [40], were comparable between the *Lrrk2*^+/+^ and *Lrrk2*^−/−^ mice (Fig. 5c). The SPNs were identified by Darpp-32 staining [41]. These data imply a potential shrinkage of individual SPNs in 12-month-old *Lrrk2*^−/−^ mouse brains.

### *Lrrk2*-deficiency reduces SPN dendritic complexity in aged *Lrrk2*^−/−^ mice

Despite SPN soma size being increased and the total number of SPNs remainingconstant, the volume of the dorsal striatum was reduced in the aged *Lrrk2*^−/−^ mice. To reconcile these seemly paradoxical observations, we further explored individual SPN morphology. We performed stereotactic injection of adeno-associated viruses (AAVs) carrying green fluorescent protein (GFP)-expressing transgene in the dorsal striatum of 2- and 12-month-old *Lrrk2*^+/+^ and *Lrrk2*^−/−^ mice. Using a low viral titer, we managed to label only a few SPNs by GFP in each hemisphere for 3D reconstruction of individual SPN soma and dendritic trees (Fig. 5d). Subsequent dendritic complexity analyses revealed substantial reductions in the total numbers of dendritic branches and cumulative length of all dendrites in the SPNs of the 12-month-old *Lrrk2*^−/−^ mice compared to age-matched controls (Fig. 5e-g). Consistent with our earlier findings (Fig. 3), the soma volume was also markedly increased in the *Lrrk2*^−/−^ SPNs (Fig. 5h). These morphological changes were age-dependent, since we did not detect any apparent alterations in SPN dendritic complexity, length, or soma volume in the 2-month-old *Lrrk2*^−/−^ mice compared to the age-matched controls (Additional file 4: Figure S2). Together, these findings suggest that dendritic atrophy contributes to the reduced volume of dorsal striatum in the aged *Lrrk2*^−/−^ mice.

### *Lrrk2*^−/−^ mice develop age-dependent motor abnormalities

**Fig. 6:**
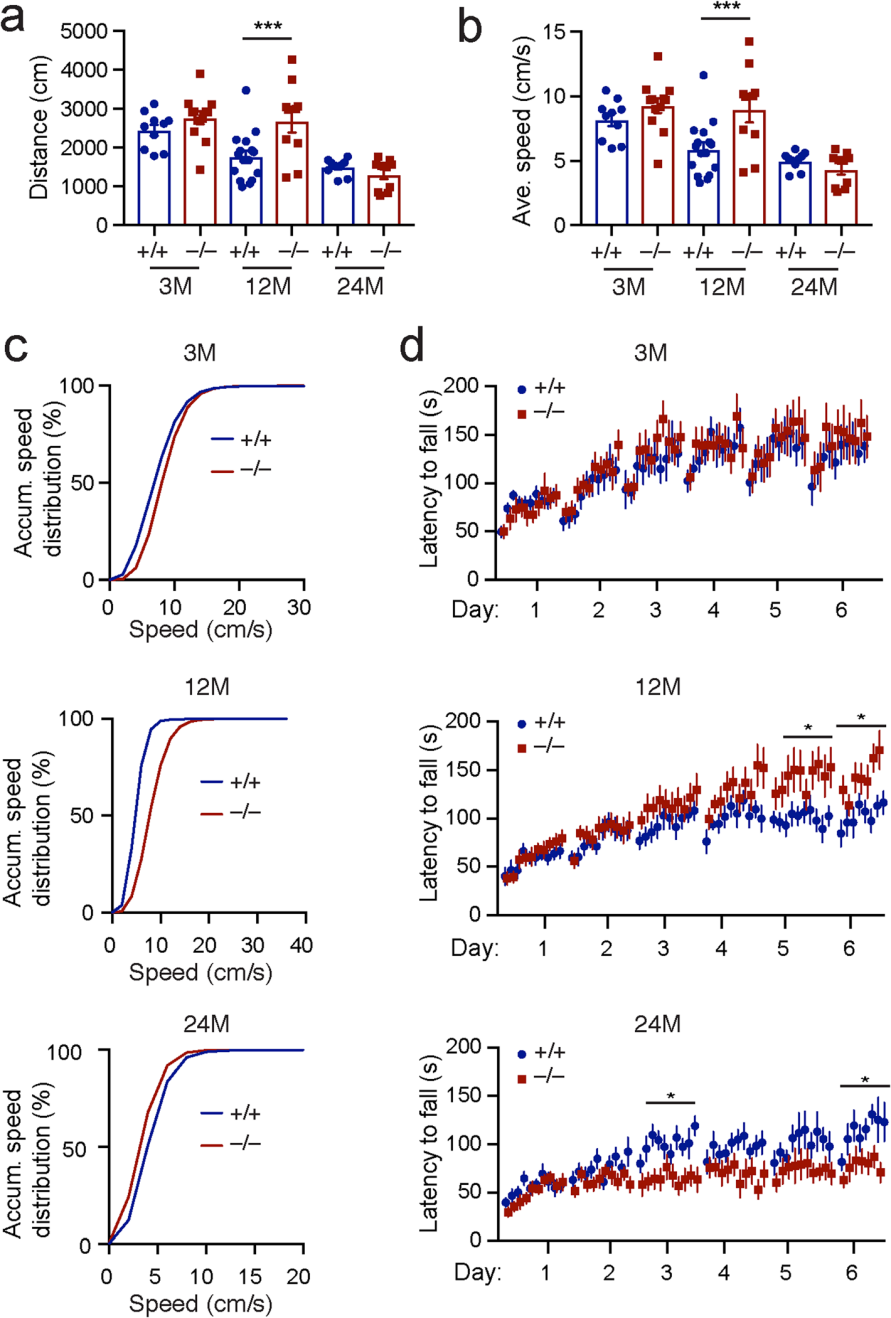
*Lrrk2*^−/−^ mice developed age-dependent motor abnormalities. **a, b** The travel distance (**a**) and average speed (**b**) of *Lrrk2*^+/+^ and *Lrrk2*^−/−^ mice at 3 (*n* = 10 and 12 for *Lrrk2*^+/+^ and *Lrrk2*^−/−^ mice, respectively), 12 (n = 10 and 11 for *Lrrk2*^+/+^ and *Lrrk2*^−/−^ mice, respectively) and 24 months of age (*n* = 12 mice per genotype). 2way ANOVA analysis with Sidak’s multiple comparisons test, ^***^p = 0.0008 (travel distance), ^***^p = 0.0008 (average speed). **c** Accumulative (Accum.) frequency of speed distribution in *Lrrk2*^+/+^ and *Lrrk2*^−/−^ mice at 3 months, 12 months and 24 months of age. **d** The latency to fall from rotarod was recorded from the same cohorts of *Lrrk2*^+/+^ and *Lrrk2*^−/−^ mice at 3, 12 and 24 months of age. 2way ANOVA analysis with Sidak’s multiple comparisons test at 12 months, ^*^p = 0.0308 (day 5), ^*^p = 0.0266 (day 6). 2way ANOVA analysis with Sidak’s multiple comparisons test at 24 months, ^*^p = 0.016 (day 3), ^*^p = 0.0124 (day 6)

To evaluate the impact of the age-dependent molecular and neuronal morphological alterations on the function of *Lrrk2*-deficient neurons, we conducted Open-field and rotarod motor skill learning tests with 3-, 12-, and 24-month-old *Lrrk2*^+/+^ and *Lrrk2*^−/−^ mice. We previously showed that the postnatal 21-day old *Lrrk2*^−/−^ mice display hyperactivity in the Open-field test [5]. Here we found that the 3- and 12-month-old *Lrrk2*^−/−^ mice also traveled longer distance and moved more frequently at higher walking speed compared to age-matched controls (Fig. 6a-c). In contrast, the 24-month-old *Lrrk2*^−/−^ mice walked more often at lower speed compared to controls (Fig. 6c). We next examined the motor skill learning of *Lrrk2*^+/+^ and *Lrrk2*^−/−^ mice using repeated rotarod tests [30, 42]. The 3- and 12-month-old *Lrrk2*^−/−^ mice performed equally well or better than age-matched control mice during the 6-day trials, while the 24-month-old *Lrrk2*^−/−^ mice showed markedly less improvements after the first 2 days’ training (Fig. 6d). These results demonstrate that *Lrrk2* is physiologically involved in the regulation of motor control and motor skill learning, although the related cell type- and circuit-specific mechanisms remain to be determined.

### *Lrrk2*-deficiency causes nuclear hypertrophy and increases nuclear invaginations in SPNs after prolonged culture

**Fig. 7:**
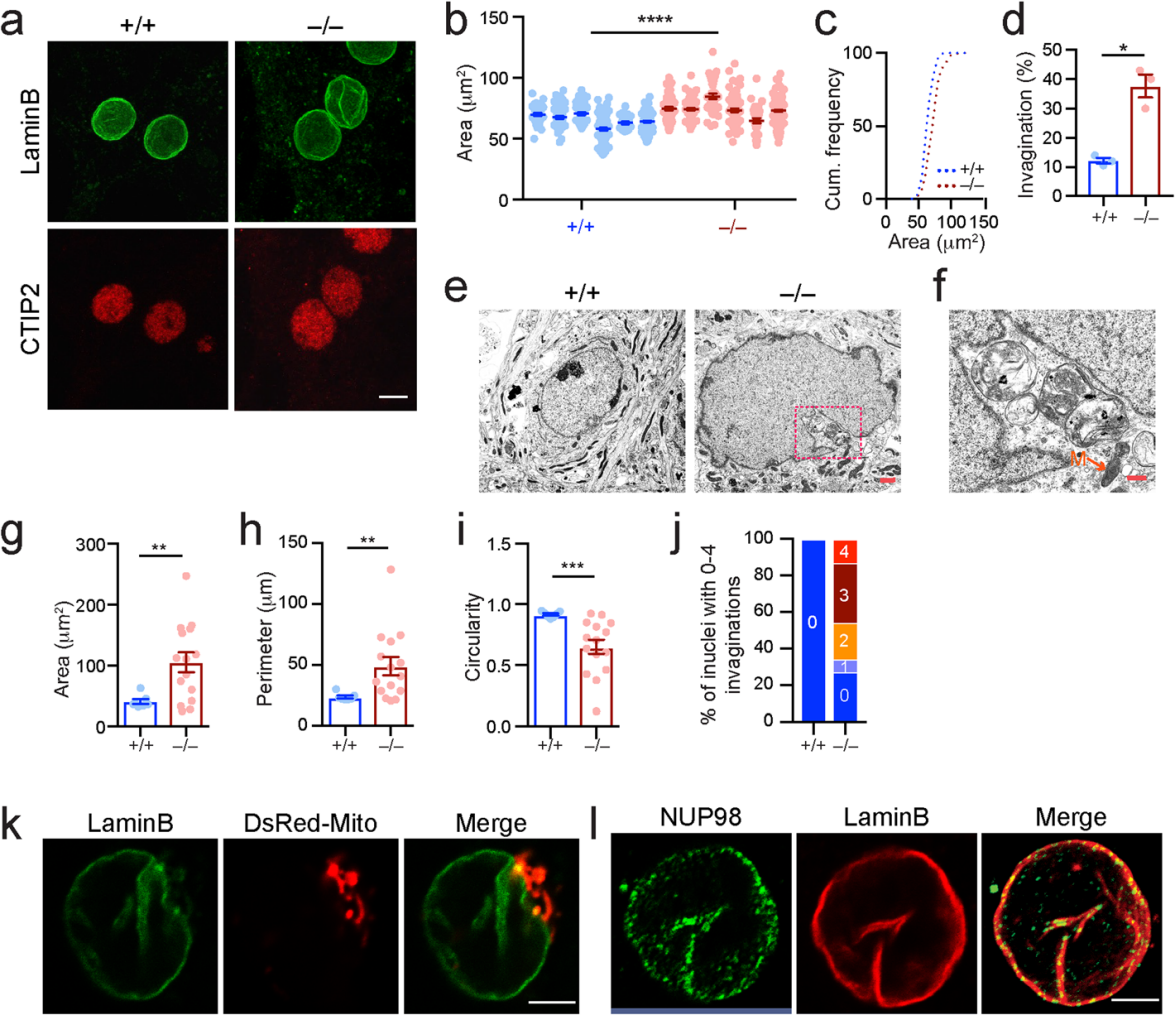
*Lrrk2*-deficiency caused nuclear hypertrophy and increased nuclear invaginations in SPNs after prolonged culture. **a** Co-staining of Lamin B and CTIP2 in *Lrrk2*^+/+^ and *Lrrk2*^−/−^ SPNs after 3 weeks in culture. Scale bar, 10 μm. **b**, **c** The areas of SPN nuclei were measured from six independent *Lrrk2*^+/+^ and *Lrrk2*^−/−^ cultures (**b**) and cumulative (Cum.) frequency was calculated to show the nuclear size distribution in each genotype (**c**). *N* = 300 neurons per genotype. Conditional logistic regression test, ^****^p < 0.0001. **d** Ratio of SPN nuclei containing ≥1 invagination was calculated from three independent *Lrrk2*^+/+^ and *Lrrk2*^−/−^ cultures. *N* = 200 neurons per genotype. Unpaired t-test, ^*^p = 0.0181. **e**, **f** Sample EM images of cultured *Lrrk2*^+/+^ and *Lrrk2*^−/−^ striatal neurons (**e**). The boxed area was shown in **f**. M indicates mitochondria. Scale bars: 2 μm (e), 0.5 μm (f). **g**-**j** The nuclear area (**g**), perimeter (**h**), and circularity (**i**), as well as the ratio of nuclei containing 0 to 4 invaginations (**j**) were calculated from the EM images. *N* = 7 and 15 neurons for *Lrrk2*^+/+^ and *Lrrk2*^−/−^ cultures. Unpaired t-test, ^**^p = 0.0018 (area), ^**^p = 0.0045 (perimeter), ^***^p = 0.0005 (circularity). **k** Co-staining of Lamin B and dsRed-Mito in a *Lrrk2*^−/−^ striatal neuron. Scale bar, 5 μm. **l** Co-staining of NUP98 and Lamin B in a *Lrrk2*^−/−^ striatal neuron. Scale bar, 5 μm

In an attempt to recapitulate our in vivo findings in cell cultures, a more amendable system for the future mechanistic studies, we also performed preliminary studies on primary cultured SPNs derived from the *Lrrk2*^+/+^ and *Lrrk2*^−/−^ mice as well as the PD-related G2019S and R1441C mutant mice. We first determined whether a loss of *Lrrk2* could cause similar nuclear morphological changes in cultured SPNs. We found that nuclear sizes were substantially larger, and the occurrences of nuclear invaginations were remarkably increased, in the *Lrrk2*^−/−^ SPNs compared to the *Lrrk2*^+/+^ controls after 3 weeks in culture (Fig. 7a-d). In contrast, no apparent alterations of nuclear or soma size were observed in *Lrrk2*^−/−^ SPNs cultured for less than 2 weeks (Additional file 5: Figure S3). The nuclear morphological abnormalities observed in *Lrrk2*^−/−^ striatal neurons after prolonged culture were confirmed by EM observations (Fig. 7e-j). It appeared that both outer and inner nuclear membranes were enfolded, a feature of type II nuclear invagination [40] (Fig. 7f). Consistent with the in vivo findings (Fig. 4g), we identified clusters of mitochondria near the mouth of nuclear invagination in the EM and fluorescent images of cultured *Lrrk2*^−/−^ striatal neurons (Fig. 7e, f, k). Why the mitochondria reside near or inside the nuclear invaginations remain speculative. With the current understanding of the physiological functions of mitochondria, we suspect the accumulation of mitochondria may provide extra ATP and/or calcium buffering capacity to protect against the deformation of nuclear structures. In addition, the nuclear pore structures marked by staining with the antibodies against nuclear pore complex protein NUP98 were also identified in the enfolded nuclear envelop (Fig. 7l), which further confirms the presence of type II nuclear invagination in the *Lrrk2*^−/−^ neurons. Therefore, the in vivo *Lrrk2*-deficiency-induced, age-dependent nuclear morphological abnormalities are recapitulated in the cultured *Lrrk2*^−/−^ SPNs after prolonged cultures.

### The malfunction of *LRRK2* kinase and GTPase domains induces differential nuclear morphological alterations

**Fig. 8:**
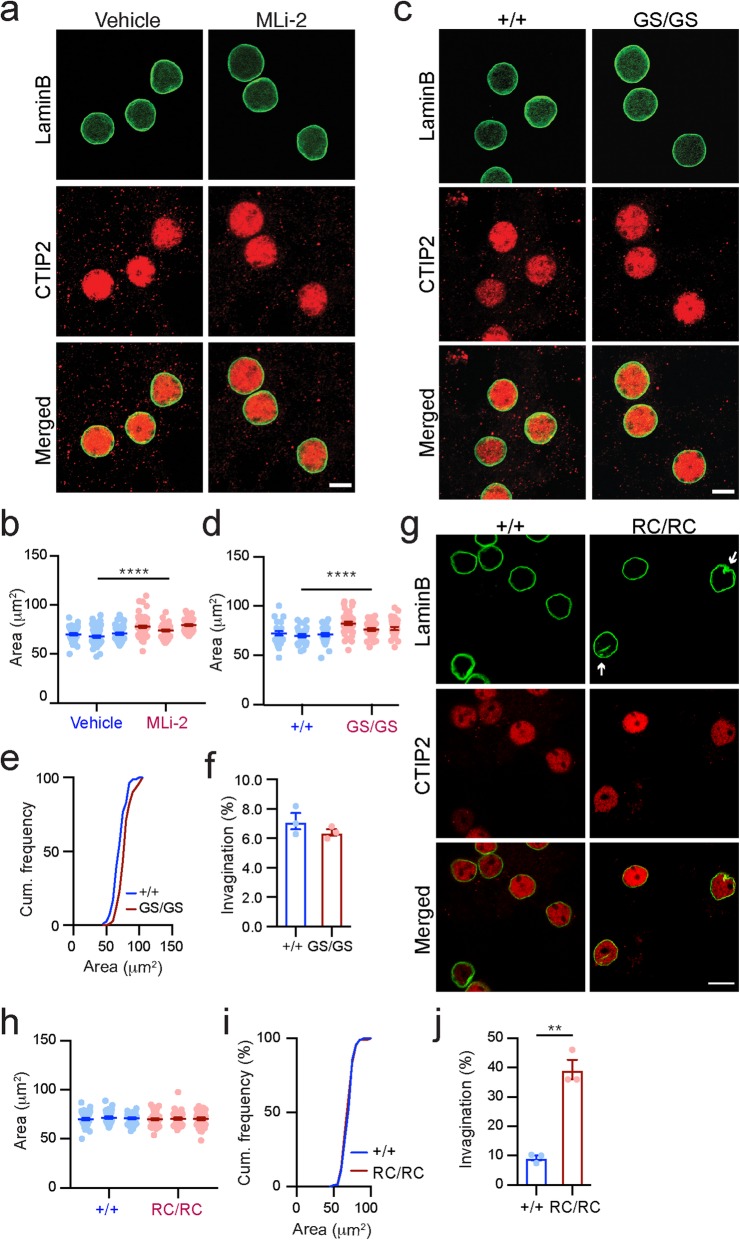
The malfunction of *LRRK2* kinase and GTPase domains induces differential nuclear morphological alterations. **a**, **b** Co-staining of Lamin B and CTIP2 in 3-week *Lrrk2*^+/+^ SPN cultures treated with vehicle or MLi-2 (**a**). Scale bar, 5 μm. The area of SPN nuclei was measured from three independent *Lrrk2*^+/+^ cultures (**b**). *N* = 150 neurons per treatment. Conditional logistic regression test, ^****^p < 0.0001. **c** Co-staining of Lamin B and CTIP2 in the *Lrrk2*^+/+^ and *Lrrk2* G2019S SPNs after 3 weeks in culture. Scale bar, 5 μm. **d**-**f** The area of SPN nuclei was measured from three independent *Lrrk2*^+/+^ and *Lrrk2* G2019S cultures (**d**). *N* = 100 neurons per genotype. Conditional logistic regression test, ^****^p < 0.0001. Cumulative (Cum.) frequency was calculated to show the nuclear size distribution in each genotype (**e**). Ratio of SPN nuclei containing ≥1 invagination was calculated from three independent *Lrrk2*^+/+^ and *Lrrk2* G2019S cultures (**f**). N = 100 neurons per genotype. Paired t-test, no statistically significant difference was identified. **g** Co-staining of Lamin B and CTIP2 in *Lrrk2*^+/+^ and *Lrrk2* R1441C SPNs after 3 weeks in culture. Scale bar, 5 μm. **h**-**j** The area of SPN nuclei was measured from three independent *Lrrk2*^+/+^ and *Lrrk2* R1441C cultures (**h**). N = 100 neurons per genotype. Conditional logistic regression test, no statistically significant difference was identified. Cumulative (Cum.) frequency was calculated to show the nuclear size distribution in each genotype (**i**). Ratio of SPN nuclei containing ≥1 invagination was calculated from three independent *Lrrk2*^+/+^ and *Lrrk2* R1441C cultures (**j**). N = 100 neurons per genotype. Paired t-test, ^**^p = 0.0092

LRRK2 protein possesses multiple functional and structural domains, including a protein kinase domain and a small GTPase domain [10]. To investigate whether *LRRK2* kinase activity regulates nuclear morphology, we treated cultured *Lrrk2*^+/+^ SPNs for 24 h with 3 nM MLi-2, a potent and selective LRRK2 kinase inhibitor [43]. We found that application of MLi-2 caused substantial increase of nuclear size but not invagination in the treated neurons (Fig. 8a, b). The PD-related G2019S missense mutation in *LRRK2* kinase domain is generally regarded to cause an increase of *LRRK2* kinase activity [10]. We next cultured SPNs from *Lrrk2* G2019S homozygous KI (GS/GS) mice for 3 weeks and found that G2019S mutation caused a similarly substantial increase in nuclear size, with only a modest reduction in nuclear invaginations compared to controls (Fig. 8c-f). These results suggest that *LRRK2* kinase activity is involved in regulating the nuclear size and that such regulation is a delicate process, i.e., either high or low kinase activity could lead to nuclear hypertrophy. Unlike the G2019S mutation, PD-related R1441C mutation in the Ras of comlex proteins (ROC) domain altered the nuclear shape but not the nuclear size in the cultured SPNs from *Lrrk2* R1441C homozygous KI (RC/RC) mice compared to the controls (Fig. 8g-j). Together, these data imply that different LRRK2 functional domains are involved in regulating the nuclear size and shape of SPNs.

### Suppression of neural activity reduces nuclear invaginations in cultured *Lrrk2*^−/−^ SPNs

**Fig. 9:**
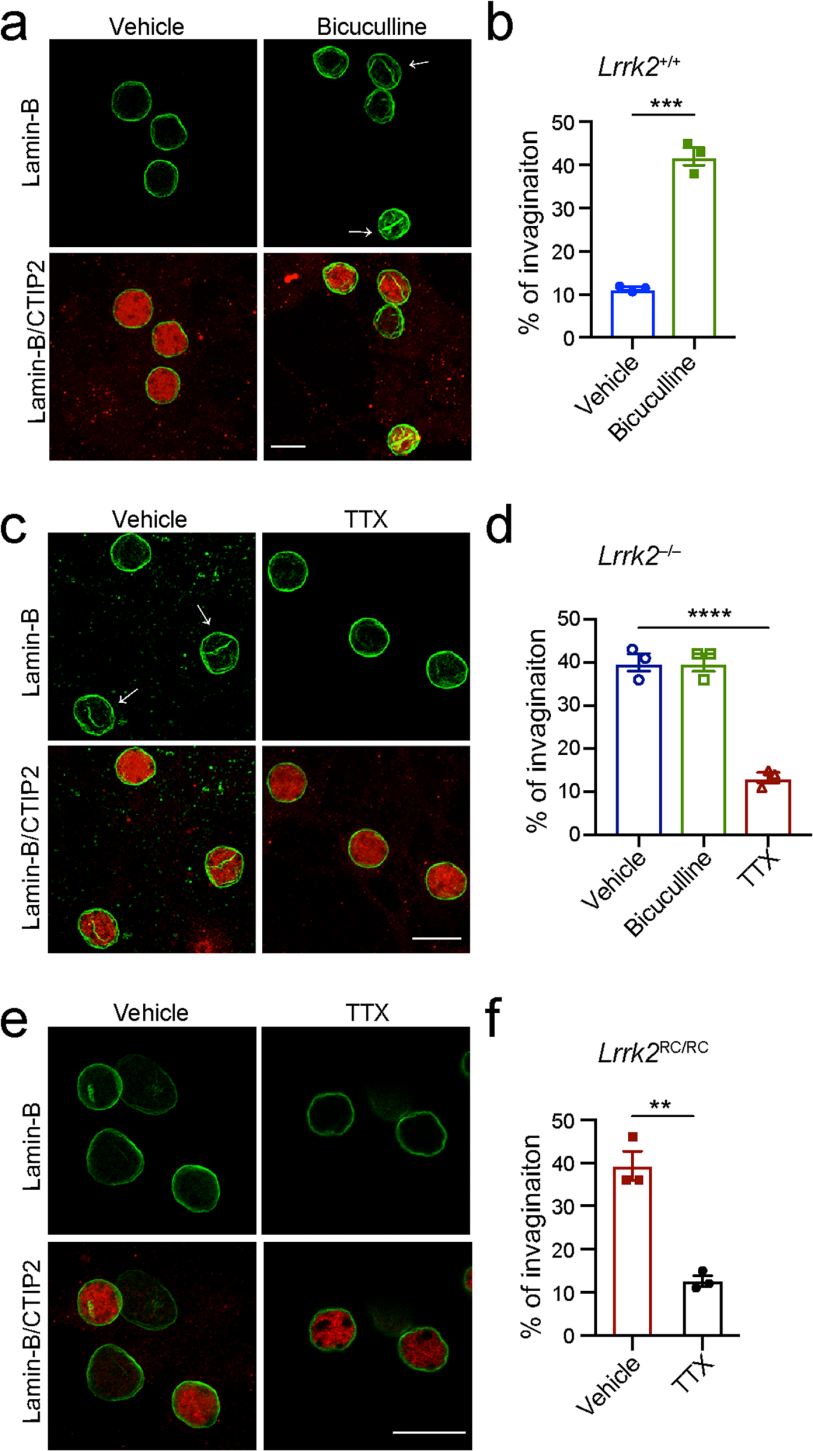
Suppression of neural activity reduces nuclear invaginations in cultured *Lrrk2*^−/−^ SPNs. **a** Co-staining of Lamin B and CTIP2 in the *Lrrk2*^+/+^ SPN cultures treated with vehicle or bicuculline. Scale bar, 10 μm. Arrow marks the site of nuclear invagination. **b** Percentage of the SPN nuclei containing ≥1 invagination was calculated from three independent *Lrrk2*^+/+^ cultures treated with vehicle or bicuculline. N = 200 neurons per treatment. Unpaired t-test, ^***^p = 0.0035. **c** Co-staining of Lamin B and CTIP2 in the *Lrrk2*^−/−^ SPN cultures treated with vehicle or TTX. Scale bar, 10 μm. Arrow marks the site of nuclear invagination. **d** Percentage of SPN nuclei containing ≥1 invagination was calculated from three independent *Lrrk2*^−/−^ cultures treated with vehicle, bicuculline and TTX. N = 200, 200, and 150 neurons for vehicle, bicuculline, and TTX treatment, respectively. 1way ANOVA, ^****^p < 0.0001. **e** Co-staining of Lamin B and CTIP2 in the *Lrrk2*^RC/RC^ SPN cultures treated with vehicle or TTX. Scale bar, 10 μm. **f** Percentage of SPN nuclei containing ≥1 invagination was calculated from three independent *Lrrk2*^RC/RC^ cultures treated with vehicle and TTX. *N* = 100 neurons per treatment, respectively. Unpaired t-test, ^**^p = 0.0017

Increased neural activity has been shown to facilitate nuclear invagination formation [44]. In line with this notion, the percentage of cultured neurons with nuclear invaginations was increased from 11 to 42% in the *Lrrk2*^+/+^ SPNs after treatment with 50 μm bicuculline for 24 h (Fig. 9a, b). Application of bicuculline, an antagonist of inhibitory neurotransmitter γ-aminobutyric acid type A receptors (GABA-ARs) [45], depolarizes neurons and increases neural excitability [44]. Previous studies reported that LRRK2 regulates Na^+^/Ca^2+^ exchanger activity in neurons [46] and Na^+^/K^+^-ATPase activity in dendritic cells [47]. A lack of *Lrrk2* may therefore lead to neural depolarization and hyper-excitability. We treated the cultured *Lrrk2*^−/−^ SPNs with 1 μM sodium channel blocker tetrodotoxin (TTX) for 1 h to suppress neural activity and found that the percentage of cells with nuclear invaginations in *Lrrk2*^−/−^ SPNs was substantially reduced from 40 to 13% after TTX treatment (Fig. 9c, d). A similar reduction of nuclear invagination was also observed in the TTX-treated RC/RC SPNs compared to the vehicle-treated ones (Fig. 9e, f). These data suggest that the hyper-excitability of *Lrrk2*^−/−^ and RC/RC SPNs likely contributes to the increased formation of nuclear invaginations.

## Discussion

Irregular shapes of nuclei have been reported in the neurons of PD patients with *LRRK2*-related G2019S [18, 19] and transgenic mice carrying R1441C [20] mutations. As an extension to these early findings, our present studies demonstrate that dysfunction of LRRK2 not only caused nuclear invagination, but also led to size enlargement in SPNs. Importantly, these morphological alterations are progressive and become more severe with aging. Nuclear hypertrophy has been reported previously in aged rat neurons [48]. The increase of nuclear size likely reflects increased biosynthetic activities of DNA repair/synthesis, transcription, and translation in cells [49]. It has been shown that *LRRK2* G2019S mutation promotes protein translation [50]. However, *LRRK2*-deficiency as well as R1441C or G2019S mutation also impairs export and maturation of newly synthesized proteins [6, 51]. We thereby suspect that the nuclear hypertrophy likely serves as an adaptive response to boost production of functional proteins in the *LRRK2* mutant SPNs.

Nuclear invagination has been well documented in various cell types during development and pathological conditions [52]. Nuclear invagination is formed at least in part to assist endoplasmic reticulum (ER) in sequestering cytoplasm calcium [52], since the outer membrane of the nuclear envelop is connected with ER membranes [53]. A high excitability has been reported in neurons [54] and immune cells [47] with different *LRRK2* mutations, which could lead to increased calcium influx, resulting in the formation of nuclear invagination. Therefore, the observed alterations of nuclear morphology likely represent compensatory responses in the SPNs against the dysfunction of *LRRK2* during aging. However, the resulting downstream effects might damage the genome integrity and eventually lead to the aberrant dopamine responses and motor symptoms seen in PD. While we observed increased γH2AX and decreased H3K9me2 levels in the striatal tissues of 12-month-old *Lrrk2*^−/−^ mice compared to age-matched controls, we found only an increase of γH2AX but no change of H3K9me2 levels in the 24-month-old *Lrrk2*^−/−^ animals. Similarly, there is also no significant change of nuclear size in the SPNs of 24-month-old *Lrrk2*^−/−^ mice compared to age-matched controls. Considering that DNA methylation affects chromatin organization and arrangement, we suspect the alteration of H3K9me2 levels is more closely correlated with nuclear size. On the other hand, DNA damage as marked by elevated γH2AX levels might be persistent and accumulative during the aging process even though there is no apparent continued enlargement of *Lrrk2*^−/−^ SPN nuclei.

As an initial attempt to understand how LRRK2 regulates nuclear morphology, we explored the roles of LRRK2’s different functional domains in the process. We found that the G2019S missense mutation in the LRRK2 kinase domain led to an increase of nuclear size, but not nuclear invagination in the SPNs. It needs to be pointed out that irregular nuclear shapes were previously reported in human neural stem cells derived from patients carrying *LRRK2* G2019S mutations after extended rounds of cell subcultures, as well as in patients’ hippocampal dentate gyrus granule cells and dopaminergic neurons [18, 19]. These studies suggest that the LRRK2 G2019S mutation may disrupt the organization of nuclear lamins and lead to the irregular nuclear shape [18, 19]. In our studies, we did not detect any increase in nuclear invaginations in the SPNs of *Lrrk2* G2019S KI mice, but did observe nuclear enlargement similar to the *Lrrk2*^−/−^ mice. This discrepancy may reflect cell type difference in response to the same disease-related genetic insults. On the other hand, the R1441C mutation in the ROC domain of LRRK2 increased the occurrence of nuclear invaginations, with little effects on the nuclear size. These results suggest differential effects of LRRK2 functional domains on regulating the nuclear size and shape of SPNs, such that the R1441C and G2019S mutations each seem to partially compromise the activity of LRRK2 in maintaining the integrity of nuclear morphology during aging. However, the underlying mechanisms remain to be determined.

Since the abnormal enhancement of LRRK2 kinase activity is implicated in the PD-related LRRK2 mutants, including the most common G2019S mutation, extensive efforts have been devoted to the development of LRRK2 kinase inhibitors as potential therapeutic agents [10]. However, increasing evidence also highlights the functional significance of LRRK2 in various cellular processes in the brain and peripheral organs. For example, the genetic deletion of *Lrrk2* in mice causes impairments of synaptogenesis during postnatal brain development [24], as well as an age-dependent, autophagy dysfunction in the kidney [55]. Our present study further reveals an involvement of LRRK2 in maintaining the nuclear integrity of dopaminoreceptive SPNs during aging. In support of the functional significance of LRRK2 in neuronal aging, we show an acceleration of age-dependent nuclear hypertrophy in the *Lrrk2*^−/−^ SPNs as well as impaired motor control in the aged *Lrrk2*^−/−^ mice. Therefore, prudent clinical trials should keep in mind of these observed side-effects of LRRK2 kinase inhibitors. Paradoxically, the LRRK2 G2019S mutant, which enhances the kinase activity, also leads to nuclear morphological abnormalities similar to the LRRK2 lost-of-function mutation in human neural stem cells [18], human dopaminergic and cortical neurons [18, 19], and mouse SPNs, suggesting an optimal level of LRRK2 kinase activity is required for maintaining the nuclear structure integrity. As a result, therapeutic benefits of LRRK2 kinase inhibitors could only arise upon striking a balance between too much and too little LRRK2 kinase activity.

There is a consensus accepted that missense mutations may alter the conformation of LRRK2 proteins and thereby increase the kinase activity of LRRK2 [10]. On the other hand, the resultant conformation changes and increased autophosphorylation may also interfere with the dimerization of LRRK2 monomers as well as impair the interactions of LRRK2 with other molecular targets, leading to potential loss-of-function phenotypes. However, it would be premature to conclude that LRRK2 is all about the kinase activity. The GTPase activity possessed by the ROC-COR domain of LRRK2 remains under studied [10]. We previously have demonstrated that the R1441C mutation in the ROC domain induces distinct biochemical and subcellular phenotypes compared to the G2019S mutation. For example, we found that the LRRK2 R1441C mutant disrupts the interaction of LRRK2 with protein kinase A subunits [5] and ER protein Sec16A [6], resulting in the same loss-of-function phenotypes as the *Lrrk2* null mutation. By contrast, the G2019S mutation does not exert such effects. Similarly, in our present studies we found that the *Lrrk2* G2019S and R1441C mutant SPNs recapitulate different aspects of nuclear morphological abnormalities exhibited in the *Lrrk2*-deficient SPNs. Consistent with our studies, a recent study demonstrates that both LRRK2 G2019S and R1441C mutations disrupt the interactions between LRRK2 and nuclear cytoskeleton protein LaminA/C and cause nuclear morphological abnormalities [19]. Together, we propose that these missense mutations may exert a dominant negative effect and compromise the normal physiological functions of LRRK2, resulting in the disruption of nuclear integrity. Our previous work also demonstrates an age-dependent reduction of LRRK2 expression in the striatal and other brain regions [23]. Considering that an important function of LRRK2 appears to be maintaining the integrity of nuclear morphology, reduced expression of LRRK2 in the aged brain might contribute to the alterations of nuclear structures observed in aged wild-type mice.

Synaptic activity is closely correlated with nuclear invagination [44]. In line with this notion, we found that fewer *Lrrk2*^−/−^ SPNs showed nuclear invagination after treatment with TTX, a potent neural activity inhibitor. By contrast, more *LRRK2*^+/+^ neurons displayed nuclear invagination after treatment with bicuculline, which increases neural activity by suppressing the GABA-AR-mediated inhibitory synaptic inputs. These observations suggest that increased nuclear invagination likely results from the hyper-excitability of *Lrrk2*^−/−^ and R1441C KI neurons. The increase of K^+^ channel expression in the *Lrrk2*^−/−^ striatal neurons might serve as a compensatory mechanism to mitigate the hyper-excitability of *Lrrk2*^−/−^ neurons. Future experiments will be needed to monitor firing rates of SPNs in the *Lrrk2*^−/−^, R1441C and G2019S KI mice during aging, since the alterations of neural firing could serve as a key indicator for the disruptions of various homeostatic networks in the brain [56]. More detailed biochemical and electrophysiological mechanisms of how LRRK2 regulates the neuronal excitability are also needed.

## Conclusions

Here we identify abnormal nuclear hypertrophy and invagination as two aging markers in *Lrrk2*^−/−^ SPNs. Different functional domains of LRRK2 may regulate nuclear morphology during aging. We propose that nuclear structural alterations reflect a compensatory response in the *Lrrk2*^−/−^ SPNs to cope with the dysregulation of various homeostatic networks during aging. As reported, LRRK2 is involved in the dynamic regulation of actin and microtubule networks [10, 57], as well as nuclear lamina [18, 19], thus more detailed mechanistic studies will be required to further elucidate the contribution of actin, microtubule, and lamina dysregulation to the nuclear hypertrophy and nuclear invagination occurring in *LRRK2* mutant neurons.

## Supplementary information

**Additional file 1: Table S1.** Differentially expressed genes with adjust *p* < 0.05 in 3-month-old *Lrrk2*^+/+^ and *Lrrk2*^−/−^ mice.

**Additional file 2: Table S2.** Differentially expressed genes with adjust p < 0.05 in 12-month-old *Lrrk2*^+/+^ and *Lrrk2*^−/−^ mice.

**Additional file 3: Figure S1.** No alteration of nuclear size in midbrain dopaminergic neurons and hippocampal neurons of 12-month-old *Lrrk2*^−/−^ mice. a-c Co-staining of Lamin B and TH in SNc and VTA neurons of 12-month-old *Lrrk2*^+/+^ and *Lrrk2*^−/−^ mice (a). Scale bar, 20 μm. The areas of soma and nuclei in SNc neurons were measured from five 12-month-old *Lrrk2*^+/+^ and *Lrrk2*^−/−^ mice (b). *N* = 5 mice per genotype, about 30 neurons counted per animal. Conditional logistic regression test, no statistically significant difference was identified. The areas of soma and nuclei in VTA neurons were measured from five 12-month-old *Lrrk2*^+/+^ and *Lrrk2*^−/−^ mice (c). N = 5 mice per genotype, about 30 neurons counted per animal. Conditional logistic regression test, no statistically significant difference was identified. d-f Co-staining of Lamin B and MAP2 in hippocampal dentate gyrus (DG) neurons of 12-month-old *Lrrk2*^+/+^ and *Lrrk2*^−/−^ mice (d). Scale bar, 20 μm. The area of nuclei in DG neurons was measured from three 12-month-old *Lrrk2*^+/+^ and *Lrrk2*^−/−^ mice e). *N* = 3 mice per genotype, about 50 neurons counted per animal. Conditional logistic regression test, no statistically significant difference was identified. The circularity of nuclei in DG neurons was measured from three 12-month-old *Lrrk2*^+/+^ and *Lrrk2*^−/−^ mice (f). N = 3 mice per genotype, about 50 neurons counted per animal. Conditional logistic regression test, no statistically significant difference was identified.

**Additional file 4: Figure S2.** No alteration of dendritic complexity in the SPNs of 3-month-old *Lrrk2*^−/−^ mice. a GFP-labeled SPNs (top panel). 3D reconstruction of the top fluorescent image (bottom panel). Scale bar, 20 μm. b, c Sholl analysis of dendritic complexity GFP-labeled SPNs. N = 5 mice per genotype, 5–9 neurons per animal. Benjamin-Hochberg multiple comparison test, no statistically significant difference was identified. d Dendritic length of GFP-labeled SPNs. N = 5 mice per genotype, 5–9 neurons per animal. Unpaired t-test, *p* = 0.195. e Soma volume of GFP-labeled SPNs. N = 5 mice per genotype, 5–9 neurons per animal. Unpaired t-test, *p* = 0.151.

**Additional file 5: Figure S3.** No alteration of nuclear and soma size of the *Lrrk2*^−/−^ SPNs after 2 weeks in culture. a Co-staining of βIII-tubulin and DAPI of the cultured SPNs. Scale bar, 20 μm. b Unpaired t-test, *n* = 50 neurons per genotype.

## Data Availability

All gene expression data will be deposited in public domains and all reagents will be available upon request.

## Abbreviations

AAV: Adeno-associated virus
DLS: Dorsal lateral striatum
GABA-AR: γ-aminobutyric acid type A receptor
KI: Knock-in
LRRK2: Leucine-rich repeat kinase 2
*Lrrk2*^−/−^: *Lrrk2* knockout
*Lrrk2*^+/+^: *Lrrk2* wild type
P0: Postnatal day 0
PD: Parkinson’s disease
SPN: Striatal spiny projection neuron
TTX: Tetrodotoxin

## References

[1] Paisan-Ruiz C, Jain S, Evans EW, Gilks WP, Simon J, van der Brug M (2004). Lopez de Munain a, Aparicio S, Gil AM, khan N, et al: cloning of the gene containing mutations that cause PARK8-linked Parkinson's disease. Neuron.

[2] Rodriguez M, Rodriguez-Sabate C, Morales I, Sanchez A, Sabate M (2015). Parkinson's disease as a result of aging. Aging Cell.

[3] Berg D, Schweitzer KJ, Leitner P, Zimprich A, Lichtner P, Belcredi P, Brussel T, Schulte C, Maass S, Nagele T (2005). Type and frequency of mutations in the LRRK2 gene in familial and sporadic Parkinson's disease*. Brain.

[4] Healy DG, Falchi M, O'Sullivan SS, Bonifati V, Durr A, Bressman S, Brice A, Aasly J, Zabetian CP, Goldwurm S (2008). Phenotype, genotype, and worldwide genetic penetrance of LRRK2-associated Parkinson's disease: a case-control study. Lancet Neurol.

[5] Parisiadou L, Yu J, Sgobio C, Xie C, Liu G, Sun L, Gu XL, Lin X, Crowley NA, Lovinger DM, Cai H (2014). LRRK2 regulates synaptogenesis and dopamine receptor activation through modulation of PKA activity. Nat Neurosci.

[6] Cho HJ, Yu J, Xie C, Rudrabhatla P, Chen X, Wu J, Parisiadou L, Liu G, Sun L, Ma B (2014). Leucine-rich repeat kinase 2 regulates Sec16A at ER exit sites to allow ER-Golgi export. EMBO J.

[7] Bonet-Ponce L, Cookson MR (2019). The role of Rab GTPases in the pathobiology of Parkinson’ disease. Curr Opin Cell Biol.

[8] Islam MS, Moore DJ (2017). Mechanisms of LRRK2-dependent neurodegeneration: role of enzymatic activity and protein aggregation. Biochem Soc Trans.

[9] Martin I, Kim JW, Dawson VL, Dawson TM (2014). LRRK2 pathobiology in Parkinson's disease. J Neurochem.

[10] Berwick DC, Heaton GR, Azeggagh S, Harvey K (2019). LRRK2 biology from structure to dysfunction: research progresses, but the themes remain the same. Mol Neurodegener.

[11] Xiao B, Deng X, Ng EY, Allen JC, Lim SY, Ahmad-Annuar A, Tan EK (2018). Association of LRRK2 haplotype with age at onset in Parkinson disease. JAMA Neurol.

[12] Reeve A, Simcox E, Turnbull D (2014). Ageing and Parkinson’s disease: why is advancing age the biggest risk factor?. Ageing Res Rev.

[13] Collier TJ, Kanaan NM, Kordower JH (2011). Ageing as a primary risk factor for Parkinson's disease: evidence from studies of non-human primates. Nat Rev Neurosci.

[14] Lopez-Otin C, Blasco MA, Partridge L, Serrano M, Kroemer G (2013). The hallmarks of aging. Cell.

[15] Feser J, Tyler J (2011). Chromatin structure as a mediator of aging. FEBS Lett.

[16] Mertens J, Paquola ACM, Ku M, Hatch E, Bohnke L, Ladjevardi S, McGrath S, Campbell B, Lee H, Herdy JR (2015). Directly reprogrammed human neurons retain aging-associated Transcriptomic signatures and reveal age-related Nucleocytoplasmic defects. Cell Stem Cell.

[17] Scaffidi P, Misteli T (2006). Lamin A-dependent nuclear defects in human aging. Science.

[18] Liu GH, Qu J, Suzuki K, Nivet E, Li M, Montserrat N, Yi F, Xu X, Ruiz S, Zhang W (2012). Progressive degeneration of human neural stem cells caused by pathogenic LRRK2. Nature.

[19] Shani V, Safory H, Szargel R, Wang N, Cohen T, Elghani FA, Hamza H, Savyon M, Radzishevsky I, Shaulov L (2019). Physiological and pathological roles of LRRK2 in the nuclear envelope integrity. Hum Mol Genet.

[20] Tsika E, Kannan M, Foo CS, Dikeman D, Glauser L, Gellhaar S, Galter D, Knott GW, Dawson TM, Dawson VL, Moore DJ (2014). Conditional expression of Parkinson's disease-related R1441C LRRK2 in midbrain dopaminergic neurons of mice causes nuclear abnormalities without neurodegeneration. Neurobiol Dis.

[21] Giesert F, Hofmann A, Burger A, Zerle J, Kloos K, Hafen U, Ernst L, Zhang J, Vogt-Weisenhorn DM, Wurst W (2013). Expression analysis of lrrk1, lrrk2 and lrrk2 splice variants in mice. PLoS One.

[22] Mandemakers W, Snellinx A, O'Neill MJ, de Strooper B (2012). LRRK2 expression is enriched in the striosomal compartment of mouse striatum. Neurobiol Dis.

[23] Cho HJ, Liu G, Jin SM, Parisiadou L, Xie C, Yu J, Sun L, Ma B, Ding J, Vancraenenbroeck R (2013). MicroRNA-205 regulates the expression of Parkinson's disease-related leucine-rich repeat kinase 2 protein. Hum Mol Genet.

[24] Parisiadou L, Xie C, Cho HJ, Lin X, Gu XL, Long CX, Lobbestael E, Baekelandt V, Taymans JM, Sun L, Cai H (2009). Phosphorylation of ezrin/radixin/moesin proteins by LRRK2 promotes the rearrangement of actin cytoskeleton in neuronal morphogenesis. J Neurosci.

[25] Herzig MC, Kolly C, Persohn E, Theil D, Schweizer T, Hafner T, Stemmelen C, Troxler TJ, Schmid P, Danner S (2011). LRRK2 protein levels are determined by kinase function and are crucial for kidney and lung homeostasis in mice. Hum Mol Genet.

[26] Tong Y, Pisani A, Martella G, Karouani M, Yamaguchi H, Pothos EN, Shen J (2009). R1441C mutation in LRRK2 impairs dopaminergic neurotransmission in mice. Proc Natl Acad Sci U S A.

[27] Lin X, Parisiadou L, Sgobio C, Liu G, Yu J, Sun L, Shim H, Gu X-L, Luo J, Long C-X (2012). Conditional expression of Parkinson's disease-related mutant alpha-Synuclein in the midbrain dopaminergic neurons causes progressive Neurodegeneration and degradation of transcription factor nuclear receptor related 1. J Neurosci.

[28] Bakker R, Tiesinga P, Kotter R (2015). The scalable brain atlas: instant web-based access to public brain atlases and related content. Neuroinformatics.

[29] Lu XH (2017). Yang XW: genetically-directed sparse neuronal labeling in BAC transgenic mice through mononucleotide repeat Frameshift. Sci Rep.

[30] Wu J, Kung J, Dong J, Chang L, Xie C, Habib A, Hawes S, Yang N, Chen V, Liu Z (2019). Distinct connectivity and functionality of aldehyde dehydrogenase 1a1-positive Nigrostriatal dopaminergic neurons in motor learning. Cell Rep.

[31] Habig K, Walter M, Poths S, Riess O, Bonin M (2008). RNA interference of LRRK2-microarray expression analysis of a Parkinson's disease key player. Neurogenetics.

[32] Dorval V, Mandemakers W, Jolivette F, Coudert L, Mazroui R, De Strooper B, Hebert SS (2014). Gene and MicroRNA transcriptome analysis of Parkinson's related LRRK2 mouse models. PLoS One.

[33] Patro R, Duggal G, Love MI, Irizarry RA, Kingsford C (2017). Salmon provides fast and bias-aware quantification of transcript expression. Nat Methods.

[34] Valdiglesias V, Giunta S, Fenech M, Neri M (2013). Bonassi S: gammaH2AX as a marker of DNA double strand breaks and genomic instability in human population studies. Mutat Res.

[35] Nakayama J, Rice JC, Strahl BD, Allis CD, Grewal SI (2001). Role of histone H3 lysine 9 methylation in epigenetic control of heterochromatin assembly. Science.

[36] Chou CC, Zhang Y, Umoh ME, Vaughan SW, Lorenzini I, Liu F, Sayegh M, Donlin-Asp PG, Chen YH, Duong DM (2018). TDP-43 pathology disrupts nuclear pore complexes and nucleocytoplasmic transport in ALS/FTD. Nat Neurosci.

[37] Crittenden JR, Graybiel AM (2011). Basal ganglia disorders associated with imbalances in the striatal striosome and matrix compartments. Front Neuroanat.

[38] Arlotta P, Molyneaux BJ, Jabaudon D, Yoshida Y, Macklis JD (2008). Ctip2 controls the differentiation of medium spiny neurons and the establishment of the cellular architecture of the striatum. J Neurosci.

[39] Likic VA, Perry A, Hulett J, Derby M, Traven A, Waller RF, Keeling PJ, Koehler CM, Curran SP, Gooley PR, Lithgow T (2005). Patterns that define the four domains conserved in known and novel isoforms of the protein import receptor Tom20. J Mol Biol.

[40] Gerfen CR, Surmeier DJ (2011). Modulation of striatal projection systems by dopamine. Annu Rev Neurosci.

[41] Ouimet CC, Miller PE, Hemmings HC, Walaas SI, Greengard P (1984). DARPP-32, a dopamine- and adenosine 3′:5′-monophosphate-regulated phosphoprotein enriched in dopamine-innervated brain regions. III. Immunocytochemical localization. J Neurosci.

[42] Costa RM, Cohen D, Nicolelis MA (2004). Differential corticostriatal plasticity during fast and slow motor skill learning in mice. Curr Biol.

[43] Fell MJ, Mirescu C, Basu K, Cheewatrakoolpong B, DeMong DE, Ellis JM, Hyde LA, Lin Y, Markgraf CG, Mei H (2015). MLi-2, a potent, selective, and centrally active compound for exploring the therapeutic potential and safety of LRRK2 kinase inhibition. J Pharmacol Exp Ther.

[44] Wittmann M, Queisser G, Eder A, Wiegert JS, Bengtson CP, Hellwig A, Wittum G, Bading H (2009). Synaptic activity induces dramatic changes in the geometry of the cell nucleus: interplay between nuclear structure, histone H3 phosphorylation, and nuclear calcium signaling. J Neurosci.

[45] Chua HC, Chebib M (2017). GABAA receptors and the diversity in their structure and pharmacology. Adv Pharmacol.

[46] Cherra SJ, Steer E, Gusdon AM, Kiselyov K, Chu CT (2013). Mutant LRRK2 elicits calcium imbalance and depletion of dendritic mitochondria in neurons. Am J Pathol.

[47] Hosseinzadeh Z, Singh Y, Shimshek DR, van der Putten H, Wagner CA, Lang F (2017). Leucine-rich repeat kinase 2 (Lrrk2)-sensitive Na(+)/K(+) ATPase activity in dendritic cells. Sci Rep.

[48] Villena A, Diaz F, Requena V, Chavarria I, Rius F (1997). Perez de Vargas I: quantitative morphological changes in neurons from the dorsal lateral geniculate nucleus of young and old rats. Anat Rec.

[49] Koda M, Takemura G, Okada H, Kanoh M, Maruyama R, Esaki M, Li Y, Miyata S, Kanamori H, Li L (2006). Nuclear hypertrophy reflects increased biosynthetic activities in myocytes of human hypertrophic hearts. Circ J.

[50] Martin I, Kim JW, Lee BD, Kang HC, Xu JC, Jia H, Stankowski J, Kim MS, Zhong J, Kumar M (2014). Ribosomal protein s15 phosphorylation mediates LRRK2 neurodegeneration in Parkinson's disease. Cell.

[51] Khurana V, Peng J, Chung CY, Auluck PK, Fanning S, Tardiff DF, Bartels T, Koeva M, Eichhorn SW, Benyamini H (2017). Genome-scale networks link neurodegenerative disease genes to alpha-Synuclein through specific molecular pathways. Cell Syst.

[52] Drozdz MM, Vaux DJ (2017). Shared mechanisms in physiological and pathological nucleoplasmic reticulum formation. Nucleus.

[53] Malhas A, Goulbourne C, Vaux DJ (2011). The nucleoplasmic reticulum: form and function. Trends Cell Biol.

[54] Bedford C, Sears C, Perez-Carrion M, Piccoli G, Condliffe SB (2016). LRRK2 regulates voltage-gated Calcium Channel function. Front Mol Neurosci.

[55] Tong Y, Giaime E, Yamaguchi H, Ichimura T, Liu Y, Si H, Cai H, Bonventre JV, Shen J (2012). Loss of leucine-rich repeat kinase 2 causes age-dependent bi-phasic alterations of the autophagy pathway. Mol Neurodegener.

[56] Frere S, Slutsky I (2018). Alzheimer's disease: from firing instability to homeostasis network collapse. Neuron.

[57] Parisiadou L, Cai H (2010). LRRK2 function on actin and microtubule dynamics in Parkinson disease. Commun Integr Biol.

