# Correction to: Parkinson’s disease-related Leucine-rich repeat kinase 2 modulates nuclear morphology and genomic stability in striatal projection neurons during aging

**DOI:** 10.1186/s13024-020-00371-x

**Published:** 2020-03-16

**Authors:** Xi Chen, Chengsong Xie, Wotu Tian, Lixin Sun, Wang Zheng, Sarah Hawes, Lisa Chang, Justin Kung, Jinhui Ding, Shengdi Chen, Weidong Le, Huaibin Cai

**Affiliations:** 1 Transgenic Section, Laboratory of Neurogenetics, National Institute on Aging, National Institutes of Health, Building 35, Room 1A112, MSC 3707, 35 Convent Drive, Bethesda, MD 20892-3707 USA; 2 Clinical Research Center on Neurological Diseases, the First Affiliated Hospital, Dalian Medical University, Dalian, 116011 People’s Republic of China; 3 Department of Neurology, Ruijin Hospital Affiliated to Shanghai Jiao Tong University School of Medicine, Shanghai, 20025 China; 4 Computational Biology Group, Laboratory of Neurogenetics, National Institute on Aging, National Institutes of Health, Bethesda, MD 20892 USA

**Correction to: Mol Neurodegeneration**

**https://doi.org/10.1186/s13024-020-00360-0**

The original article [1] had mistakenly inverted co-author, Wang Zheng’s name. This has since been corrected.
