# Supplementary material for: Parkinson’s disease-related Leucine-rich repeat kinase 2 modulates nuclear morphology and genomic stability in striatal projection neurons during aging
Source: Mol Neurodegener. 2020 Feb 19;15:12. doi: 10.1186/s13024-020-00360-0 (PMC7031993; doi:10.1186/s13024-020-00360-0)
Supplement: Supplementary file 1 — Additional file 1: Table S1. Differentially expressed genes with adjust p < 0.05 in 3-month-old Lrrk2+/+ and Lrrk2−/− mice. [file 13024_2020_360_MOESM1_ESM.docx]

**Supplementary Table 1 Differentially expressed genes with adjust *p* <0.05 in 3-month-old *Lrrk2*^+/+^ and *Lrrk2*^-/-^ mi**ce

| **Gene_ID** | **-/-**  **#1** | **-/-**  **#2** | **-/-**  **#3** | **-/-**  **#4** | **+/+**  **#1** | **+/+**  **#2** | **+/+**  **#3** | **Base**  **Mean** | **Log2**  **Fold-Change** | **p-value** | **p-adj** |
| --- | --- | --- | --- | --- | --- | --- | --- | --- | --- | --- | --- |
| Gm12751 | 1 | 1 | 1 | 1 | 478 | 218 | 319 | 139.28 | -8.33 | 1.37E-24 | 5.21E-21 |
| Gh | 0 | 1 | 1 | 0 | 109 | 169 | 164 | 60.44 | -8.09 | 5.08E-15 | 8.95E-12 |
| Gm13841 | 12 | 12 | 16 | 54 | 18 | 7945 | 9696 | 2359.26 | -7.97 | 2.05E-08 | 1.05E-05 |
| Gm15772 | 47 | 33 | 29 | 58 | 59 | 13969 | 17340 | 4190.25 | -7.87 | 1.07E-09 | 8.17E-07 |
| Gm6969 | 1 | 1 | 7 | 3 | 1 | 1121 | 1342 | 329.58 | -7.85 | 3.97E-06 | 0.000976372 |
| Prl | 7 | 0 | 0 | 0 | 11 | 496 | 516 | 137.67 | -7.66 | 0.000512584 | 0.031884691 |
| Gm6594 | 0 | 0 | 0 | 0 | 32 | 30 | 38 | 13.56 | -7.38 | 5.23E-09 | 3.52E-06 |
| Gm15920 | 22 | 11 | 23 | 13 | 3616 | 2413 | 2903 | 1229.84 | -7.32 | 3.48E-122 | 7.97E-118 |
| Gm5506 | 93 | 7 | 8 | 53 | 26 | 7128 | 9357 | 2208.83 | -7.18 | 1.36E-06 | 0.000414149 |
| Gm13230 | 0 | 0 | 1 | 0 | 0 | 62 | 62 | 16.78 | -6.95 | 9.94E-05 | 0.011085601 |
| Gm12663 | 4 | 2 | 2 | 1 | 0 | 386 | 560 | 126.33 | -6.94 | 8.57E-05 | 0.00981353 |
| Gm5436 | 3 | 1 | 1 | 6 | 1 | 294 | 379 | 90.79 | -6.39 | 1.83E-05 | 0.003273784 |
| Gm18199 | 1 | 0 | 0 | 0 | 15 | 24 | 33 | 9.79 | -6.17 | 3.90E-06 | 0.000969278 |
| Gm3962 | 1 | 2 | 1 | 2 | 88 | 83 | 57 | 32.37 | -5.60 | 2.72E-13 | 3.90E-10 |
| Gm6685 | 2 | 3 | 3 | 8 | 0 | 173 | 222 | 54.63 | -4.97 | 2.38E-05 | 0.003886709 |
| Gm4735 | 33 | 60 | 38 | 127 | 75 | 2342 | 2585 | 703.92 | -4.62 | 9.94E-06 | 0.002056207 |
| Gm10307 | 2 | 6 | 5 | 3 | 3 | 143 | 160 | 43.48 | -4.40 | 1.66E-05 | 0.003046242 |
| Gm3978 | 1 | 1 | 1 | 1 | 12 | 34 | 18 | 9.42 | -4.33 | 6.33E-05 | 0.008009527 |
| Spink5 | 3 | 0 | 2 | 9 | 107 | 48 | 2 | 23.97 | -4.08 | 0.000606476 | 0.035682448 |
| Tma7-ps | 11 | 2 | 3 | 43 | 4 | 209 | 301 | 74.77 | -3.70 | 0.00038535 | 0.02692233 |
| Trim10 | 5 | 1 | 5 | 5 | 112 | 25 | 17 | 23.84 | -3.66 | 3.69E-05 | 0.005254543 |
| Gm10182 | 480 | 309 | 239 | 482 | 4054 | 3247 | 3992 | 1751.91 | -3.25 | 2.87E-84 | 3.28E-80 |
| Lrrk2 | 3872 | 2033 | 2806 | 6747 | 34807 | 30364 | 37912 | 16107.05 | -3.15 | 2.50E-58 | 1.91E-54 |
| Bglap | 11 | 0 | 8 | 26 | 28 | 172 | 60 | 41.63 | -3.08 | 0.000514822 | 0.031937072 |
| 4930515G13Rik | 7 | 2 | 5 | 1 | 24 | 20 | 63 | 16.40 | -3.07 | 0.00030388 | 0.02366026 |
| Gm15382 | 6 | 4 | 7 | 3 | 51 | 36 | 38 | 20.26 | -2.92 | 6.50E-06 | 0.001458115 |
| Gypa | 19 | 6 | 20 | 12 | 194 | 64 | 45 | 50.76 | -2.74 | 4.06E-05 | 0.005638409 |
| Rbpms2 | 70 | 27 | 46 | 30 | 26 | 398 | 470 | 144.87 | -2.63 | 0.00022379 | 0.019186424 |
| Gm10800 | 761 | 216 | 462 | 344 | 1863 | 3685 | 2928 | 1415.09 | -2.57 | 1.93E-12 | 2.45E-09 |
| Gm2309 | 24 | 8 | 15 | 17 | 42 | 114 | 128 | 47.33 | -2.48 | 1.74E-06 | 0.000498373 |
| Fgr | 17 | 4 | 4 | 7 | 34 | 46 | 51 | 22.14 | -2.41 | 9.98E-05 | 0.011085601 |
| Slc4a1 | 42 | 48 | 66 | 36 | 497 | 205 | 144 | 148.36 | -2.37 | 9.98E-06 | 0.002056207 |
| Gm10801 | 87 | 14 | 27 | 39 | 106 | 312 | 223 | 110.17 | -2.35 | 9.02E-06 | 0.001930591 |
| Gm3972 | 13 | 11 | 11 | 21 | 45 | 67 | 112 | 37.93 | -2.32 | 8.02E-07 | 0.000258724 |
| Gm26870 | 385 | 191 | 236 | 294 | 831 | 1793 | 1521 | 723.94 | -2.23 | 2.71E-15 | 5.17E-12 |
| Gm10719 | 73 | 50 | 71 | 15 | 162 | 432 | 263 | 151.20 | -2.23 | 0.000157434 | 0.014953646 |
| Gm10720 | 24 | 11 | 11 | 14 | 41 | 93 | 84 | 38.25 | -2.19 | 1.19E-05 | 0.00232144 |
| S100a9 | 69 | 8 | 28 | 34 | 144 | 173 | 152 | 83.03 | -2.16 | 1.30E-05 | 0.00249221 |
| Gm21738 | 30 | 5 | 31 | 14 | 45 | 131 | 112 | 50.98 | -2.14 | 0.00099847 | 0.049258554 |
| Hbb-bt | 300 | 151 | 310 | 295 | 1306 | 1263 | 978 | 642.90 | -2.07 | 4.24E-14 | 6.47E-11 |
| Gm10718 | 53 | 26 | 67 | 25 | 160 | 234 | 216 | 109.77 | -2.06 | 1.44E-05 | 0.002667299 |
| S100a8 | 34 | 5 | 18 | 28 | 61 | 119 | 86 | 47.87 | -2.06 | 7.22E-05 | 0.008766237 |
| Masp2 | 56 | 53 | 60 | 80 | 67 | 330 | 444 | 148.88 | -2.02 | 9.55E-05 | 0.010767952 |
| Gm11168 | 21 | 12 | 21 | 14 | 35 | 102 | 84 | 40.35 | -1.97 | 0.000303669 | 0.02366026 |
| Plac9a | 64 | 9 | 15 | 22 | 141 | 75 | 104 | 58.53 | -1.96 | 0.000692975 | 0.039264561 |
| Gm9816 | 11 | 6 | 7 | 21 | 38 | 33 | 61 | 23.75 | -1.94 | 0.000246061 | 0.020407945 |
| Hist1h2bl | 458 | 177 | 247 | 477 | 1237 | 1326 | 1215 | 704.06 | -1.87 | 1.24E-20 | 4.07E-17 |
| Prss56 | 120 | 75 | 130 | 271 | 455 | 540 | 631 | 302.39 | -1.84 | 4.41E-11 | 4.39E-08 |
| Duxbl3 | 57 | 10 | 25 | 18 | 116 | 83 | 102 | 56.62 | -1.80 | 0.000688827 | 0.039126418 |
| Krt26 | 140 | 181 | 233 | 355 | 1718 | 371 | 419 | 481.98 | -1.80 | 0.000542071 | 0.03291391 |
| Hba-a1 | 57 | 33 | 149 | 106 | 314 | 316 | 253 | 173.34 | -1.64 | 0.000723339 | 0.040178415 |
| Slc39a2 | 477 | 158 | 183 | 220 | 536 | 921 | 1100 | 492.43 | -1.64 | 2.36E-06 | 0.000652203 |
| Hba-a2 | 250 | 148 | 208 | 217 | 837 | 677 | 495 | 398.67 | -1.61 | 8.67E-09 | 5.37E-06 |
| Gm10282 | 286 | 93 | 191 | 266 | 774 | 505 | 659 | 381.16 | -1.58 | 9.33E-09 | 5.48E-06 |
| Tmem181c-ps | 843 | 596 | 494 | 1601 | 2244 | 2496 | 3183 | 1553.12 | -1.57 | 2.10E-12 | 2.53E-09 |
| Hist1h2bm | 777 | 256 | 443 | 811 | 2128 | 1490 | 1388 | 1001.88 | -1.54 | 9.15E-09 | 5.48E-06 |
| Hist1h3c | 530 | 269 | 361 | 593 | 1660 | 1164 | 1110 | 788.02 | -1.54 | 8.07E-13 | 1.09E-09 |
| RP23-45G16.5 | 67 | 26 | 64 | 61 | 239 | 145 | 109 | 99.87 | -1.52 | 0.000349534 | 0.02524031 |
| Hbb-bs | 1283 | 858 | 1675 | 1158 | 4062 | 4416 | 3431 | 2394.17 | -1.52 | 4.42E-07 | 0.000150981 |
| Hist1h2bk | 1449 | 707 | 926 | 1496 | 4212 | 2981 | 2982 | 2042.87 | -1.52 | 1.01E-14 | 1.65E-11 |
| Pnp2 | 539 | 127 | 127 | 241 | 903 | 563 | 754 | 443.70 | -1.51 | 0.000129112 | 0.013223203 |
| Hist1h2bn | 1473 | 538 | 800 | 1354 | 3978 | 2378 | 2410 | 1783.81 | -1.47 | 2.65E-08 | 1.29E-05 |
| Hist1h4n | 3113 | 1489 | 1284 | 3819 | 4128 | 7125 | 8688 | 4006.24 | -1.44 | 4.21E-08 | 2.01E-05 |
| Hdhd3 | 745 | 265 | 349 | 809 | 1233 | 1430 | 1745 | 891.15 | -1.43 | 2.10E-10 | 1.93E-07 |
| Hist1h2bp | 190 | 100 | 100 | 214 | 494 | 340 | 402 | 253.06 | -1.41 | 2.81E-10 | 2.48E-07 |
| Il5 | 52 | 33 | 19 | 86 | 90 | 131 | 153 | 76.08 | -1.39 | 0.000332784 | 0.024914892 |
| Urah | 60 | 36 | 40 | 42 | 151 | 99 | 123 | 77.27 | -1.37 | 6.07E-05 | 0.007763888 |
| Dio3os | 96 | 43 | 60 | 129 | 145 | 246 | 255 | 132.55 | -1.36 | 5.06E-06 | 0.001181495 |
| Hist1h2bh | 377 | 220 | 309 | 438 | 1155 | 760 | 796 | 564.88 | -1.36 | 1.19E-09 | 8.80E-07 |
| Hist1h1b | 1027 | 535 | 839 | 1088 | 3160 | 1864 | 1938 | 1458.32 | -1.34 | 6.00E-08 | 2.64E-05 |
| Tusc1 | 182 | 84 | 73 | 254 | 166 | 395 | 541 | 226.79 | -1.30 | 0.000910787 | 0.046537553 |
| Gm6206 | 1086 | 480 | 457 | 1442 | 1236 | 2445 | 2704 | 1327.51 | -1.30 | 1.01E-05 | 0.002056207 |
| Aurkb | 119 | 78 | 124 | 110 | 403 | 221 | 238 | 183.00 | -1.29 | 0.000166072 | 0.01558013 |
| Atp6v1c2 | 312 | 123 | 164 | 457 | 445 | 664 | 808 | 400.02 | -1.28 | 6.11E-06 | 0.001383765 |
| Edar | 124 | 102 | 164 | 124 | 378 | 329 | 323 | 219.67 | -1.26 | 7.01E-05 | 0.00862402 |
| Gm10706 | 216 | 186 | 222 | 403 | 649 | 564 | 692 | 405.59 | -1.24 | 8.24E-10 | 6.74E-07 |
| Hist1h2af | 303 | 272 | 299 | 325 | 999 | 605 | 711 | 497.36 | -1.23 | 4.83E-06 | 0.001150707 |
| Fam64a | 241 | 172 | 217 | 240 | 630 | 499 | 512 | 353.63 | -1.22 | 7.64E-08 | 3.18E-05 |
| Bspry | 227 | 111 | 176 | 269 | 354 | 514 | 547 | 302.69 | -1.21 | 6.85E-08 | 2.90E-05 |
| Zar1l | 74 | 56 | 85 | 145 | 221 | 187 | 221 | 136.71 | -1.16 | 1.96E-05 | 0.003431519 |
| Ndc80 | 163 | 98 | 189 | 156 | 488 | 316 | 285 | 240.71 | -1.14 | 0.000840864 | 0.044095192 |
| Bub1 | 199 | 143 | 255 | 283 | 667 | 396 | 498 | 342.83 | -1.14 | 9.86E-05 | 0.011064259 |
| Alad | 2240 | 1123 | 1364 | 2313 | 3376 | 3906 | 4805 | 2630.95 | -1.13 | 1.06E-15 | 2.43E-12 |
| Hist1h3b | 851 | 565 | 893 | 813 | 2564 | 1514 | 1489 | 1232.89 | -1.13 | 0.000106311 | 0.011699824 |
| Hist1h2ah | 127 | 90 | 134 | 126 | 302 | 244 | 307 | 187.44 | -1.12 | 2.49E-05 | 0.003990652 |
| Pdlim3 | 260 | 150 | 156 | 200 | 630 | 309 | 386 | 293.40 | -1.12 | 0.000255007 | 0.020997749 |
| Ttk | 145 | 84 | 119 | 142 | 348 | 233 | 264 | 187.03 | -1.11 | 1.72E-05 | 0.003102711 |
| Gucy1b2 | 65 | 32 | 42 | 66 | 151 | 89 | 105 | 76.30 | -1.11 | 0.000776461 | 0.041821107 |
| Uhrf1 | 423 | 214 | 383 | 362 | 938 | 720 | 730 | 530.42 | -1.10 | 2.00E-05 | 0.003475308 |
| Dusp2 | 112 | 58 | 34 | 129 | 148 | 178 | 210 | 117.84 | -1.09 | 0.000884118 | 0.045377461 |
| St8sia2 | 296 | 311 | 364 | 593 | 736 | 941 | 953 | 585.05 | -1.07 | 4.54E-06 | 0.00109341 |
| Plekhg4 | 325 | 161 | 236 | 354 | 426 | 565 | 790 | 392.51 | -1.06 | 2.05E-06 | 0.000579209 |
| Itgb1bp1 | 7002 | 4578 | 4005 | 9066 | 10837 | 12880 | 16406 | 8888.13 | -1.06 | 2.41E-12 | 2.76E-09 |
| Hist1h2ad | 610 | 194 | 198 | 598 | 930 | 741 | 779 | 548.60 | -1.06 | 0.000293781 | 0.023194012 |
| Cenpa | 176 | 108 | 113 | 157 | 371 | 331 | 200 | 205.46 | -1.05 | 0.000549215 | 0.033259446 |
| Kifc1 | 190 | 101 | 178 | 233 | 512 | 312 | 314 | 257.15 | -1.05 | 0.000301412 | 0.023628821 |
| Hist1h3h | 913 | 481 | 486 | 1112 | 1834 | 1368 | 1512 | 1059.06 | -1.05 | 1.06E-08 | 6.06E-06 |
| Tmem181b-ps | 4257 | 3227 | 2836 | 6842 | 8294 | 8269 | 10958 | 6130.87 | -1.04 | 1.75E-11 | 1.82E-08 |
| Hist1h1a | 1902 | 1302 | 1753 | 2332 | 5518 | 3061 | 3330 | 2696.36 | -1.04 | 2.29E-05 | 0.003805933 |
| Trib3 | 75 | 59 | 47 | 141 | 199 | 146 | 152 | 112.46 | -1.02 | 0.00088129 | 0.045377461 |
| Pthlh | 307 | 241 | 305 | 567 | 786 | 644 | 807 | 505.44 | -1.01 | 3.52E-07 | 0.000127546 |
| Hist1h3a | 736 | 350 | 466 | 758 | 1618 | 915 | 1051 | 817.45 | -1.00 | 3.14E-05 | 0.004665337 |
| H2-Q2 | 118 | 68 | 111 | 151 | 295 | 171 | 245 | 161.19 | -1.00 | 0.000343526 | 0.024963991 |
| Hist1h2ab | 1312 | 625 | 749 | 1253 | 2695 | 1636 | 1778 | 1394.14 | -1.00 | 8.83E-06 | 0.001907682 |
| Hist1h2bf | 717 | 390 | 516 | 796 | 1592 | 1068 | 1106 | 861.18 | -1.00 | 1.44E-06 | 0.000433448 |
| Hist1h2br | 1010 | 395 | 474 | 1020 | 1746 | 1247 | 1371 | 995.08 | -1.00 | 9.28E-06 | 0.00194939 |
| Top2a | 3130 | 2323 | 3316 | 2966 | 7369 | 5997 | 5864 | 4403.65 | -0.99 | 3.68E-05 | 0.005254543 |
| Knstrn | 275 | 210 | 273 | 322 | 763 | 429 | 538 | 396.31 | -0.98 | 0.000202121 | 0.017868174 |
| Cdca7 | 586 | 578 | 931 | 793 | 1864 | 1399 | 1523 | 1095.61 | -0.98 | 0.000869581 | 0.045137365 |
| Coch | 10434 | 8975 | 7355 | 15587 | 23813 | 18109 | 23798 | 14992.21 | -0.97 | 2.30E-09 | 1.64E-06 |
| Pomc | 212 | 103 | 136 | 286 | 369 | 346 | 393 | 252.56 | -0.97 | 5.91E-07 | 0.000196012 |
| Pi16 | 99 | 74 | 98 | 188 | 239 | 193 | 266 | 159.29 | -0.96 | 0.000114104 | 0.012262654 |
| B3gnt5 | 439 | 246 | 440 | 467 | 964 | 775 | 742 | 573.23 | -0.96 | 7.80E-05 | 0.009016688 |
| Kntc1 | 491 | 211 | 327 | 350 | 880 | 673 | 561 | 490.85 | -0.96 | 0.000560119 | 0.033655001 |
| Alas2 | 1295 | 1149 | 1189 | 1353 | 2893 | 2479 | 2601 | 1838.04 | -0.96 | 3.20E-06 | 0.000831904 |
| Ccnb1 | 650 | 469 | 657 | 678 | 1608 | 1069 | 1167 | 891.96 | -0.94 | 0.000115052 | 0.012306825 |
| Cdc20 | 283 | 166 | 295 | 350 | 651 | 528 | 487 | 387.53 | -0.94 | 0.000148173 | 0.014443567 |
| Hist1h4a | 1925 | 1035 | 981 | 2190 | 3166 | 2788 | 3048 | 2082.37 | -0.94 | 3.28E-10 | 2.78E-07 |
| P2ry1 | 1153 | 679 | 1284 | 2622 | 2020 | 2957 | 3351 | 1915.21 | -0.93 | 0.00095919 | 0.048469805 |
| Hist1h2ae | 516 | 295 | 194 | 534 | 808 | 664 | 778 | 520.91 | -0.93 | 4.84E-05 | 0.006590198 |
| Gabra2 | 3925 | 2707 | 3351 | 5050 | 7510 | 7090 | 8145 | 5263.76 | -0.93 | 1.43E-15 | 2.97E-12 |
| Gm10163 | 12539 | 4013 | 6216 | 12269 | 10444 | 18202 | 21891 | 11600.70 | -0.92 | 0.000726856 | 0.040178415 |
| Cdc25c | 121 | 60 | 105 | 108 | 210 | 190 | 197 | 139.09 | -0.92 | 0.000718391 | 0.040178415 |
| Exo1 | 174 | 144 | 188 | 188 | 410 | 337 | 330 | 251.90 | -0.92 | 0.000499485 | 0.031239665 |
| Hist1h2bb | 2606 | 1222 | 1504 | 2535 | 5574 | 3025 | 2766 | 2677.03 | -0.91 | 0.000823217 | 0.043620959 |
| Hist1h2ag | 330 | 230 | 220 | 384 | 781 | 396 | 548 | 402.99 | -0.91 | 0.000337185 | 0.024914892 |
| Duxbl1 | 143 | 71 | 79 | 129 | 212 | 225 | 179 | 144.51 | -0.91 | 0.000280571 | 0.022560489 |
| Ccna2 | 809 | 672 | 968 | 983 | 2013 | 1624 | 1634 | 1235.98 | -0.90 | 0.000144833 | 0.014371574 |
| Col6a1 | 2568 | 1937 | 2360 | 5203 | 3981 | 6146 | 7319 | 4037.02 | -0.90 | 0.000108001 | 0.011828974 |
| Hist1h3i | 1418 | 653 | 729 | 1628 | 2459 | 1735 | 2084 | 1467.27 | -0.90 | 3.40E-06 | 0.000864864 |
| Gm26659 | 2378 | 2355 | 2247 | 3604 | 4488 | 5075 | 6492 | 3723.73 | -0.90 | 6.60E-07 | 0.000215816 |
| Hist1h4i | 1335 | 914 | 839 | 1777 | 1876 | 2334 | 2898 | 1648.65 | -0.90 | 6.23E-09 | 3.96E-06 |
| Ncapg | 371 | 298 | 369 | 389 | 877 | 661 | 633 | 511.59 | -0.90 | 0.000297424 | 0.023396298 |
| Hist1h2bg | 3044 | 1453 | 1730 | 3371 | 5428 | 4031 | 4026 | 3186.62 | -0.88 | 2.49E-06 | 0.000678541 |
| Pou3f4 | 1005 | 844 | 1099 | 1946 | 2360 | 2162 | 2506 | 1653.93 | -0.87 | 5.45E-06 | 0.001259046 |
| Insm1 | 418 | 330 | 384 | 579 | 965 | 728 | 765 | 584.90 | -0.85 | 1.09E-05 | 0.002178411 |
| Shcbp1 | 215 | 147 | 181 | 220 | 448 | 317 | 338 | 263.21 | -0.85 | 0.000337813 | 0.024914892 |
| Mxd3 | 214 | 152 | 224 | 302 | 364 | 400 | 524 | 303.65 | -0.84 | 6.67E-05 | 0.008298919 |
| Msantd1 | 296 | 265 | 265 | 591 | 562 | 646 | 772 | 468.15 | -0.84 | 6.71E-05 | 0.008298919 |
| Tmco5 | 96 | 77 | 89 | 161 | 171 | 203 | 222 | 141.36 | -0.84 | 0.000293719 | 0.023194012 |
| Slc5a11 | 208 | 140 | 114 | 208 | 356 | 300 | 279 | 224.58 | -0.83 | 0.000237718 | 0.020005879 |
| Chat | 983 | 905 | 925 | 2152 | 2512 | 1862 | 2440 | 1623.18 | -0.83 | 0.000161692 | 0.015231696 |
| Dynlt1b | 1991 | 893 | 1219 | 1821 | 2920 | 2557 | 2630 | 1947.76 | -0.81 | 1.73E-06 | 0.000498373 |
| Sv2c | 5284 | 3629 | 3370 | 10954 | 9703 | 10943 | 9594 | 7279.57 | -0.81 | 0.00048819 | 0.030904374 |
| Hist1h4j | 867 | 560 | 638 | 1008 | 1761 | 1248 | 1210 | 1020.84 | -0.80 | 3.69E-05 | 0.005254543 |
| Kif11 | 1297 | 764 | 1352 | 1318 | 2574 | 2070 | 2029 | 1613.44 | -0.80 | 0.000773492 | 0.041759472 |
| Gm27031 | 3717 | 2739 | 2345 | 7694 | 7009 | 7473 | 6836 | 5156.36 | -0.79 | 0.000531834 | 0.032624845 |
| Efcab7 | 449 | 199 | 354 | 678 | 653 | 750 | 812 | 532.49 | -0.79 | 0.000386049 | 0.02692233 |
| Nuf2 | 363 | 258 | 329 | 408 | 778 | 533 | 576 | 457.86 | -0.79 | 0.000434671 | 0.02916669 |
| Fndc9 | 786 | 447 | 454 | 914 | 795 | 1283 | 1417 | 838.58 | -0.78 | 0.000115848 | 0.012334292 |
| Rreb1 | 764 | 522 | 507 | 880 | 1296 | 1031 | 1300 | 877.20 | -0.78 | 1.14E-08 | 6.38E-06 |
| Pim1 | 314 | 182 | 237 | 388 | 537 | 443 | 523 | 364.06 | -0.77 | 1.66E-06 | 0.000486027 |
| Gm1043 | 610 | 446 | 447 | 878 | 1405 | 816 | 929 | 770.11 | -0.77 | 0.000820223 | 0.043620959 |
| Gm872 | 1945 | 1345 | 1923 | 1876 | 2648 | 3355 | 3917 | 2402.20 | -0.76 | 0.000456164 | 0.029971704 |
| Galr1 | 248 | 185 | 190 | 408 | 504 | 355 | 499 | 329.56 | -0.76 | 0.000206033 | 0.018125332 |
| Trpc7 | 668 | 386 | 535 | 1071 | 1002 | 1117 | 1352 | 840.44 | -0.76 | 1.93E-05 | 0.003390101 |
| Hist1h4k | 2200 | 1608 | 1549 | 3697 | 4778 | 3143 | 3816 | 2866.70 | -0.76 | 0.000145751 | 0.014371574 |
| Sox11 | 1147 | 547 | 808 | 1320 | 1920 | 1548 | 1494 | 1218.03 | -0.75 | 6.45E-05 | 0.008109865 |
| Iqgap3 | 275 | 160 | 211 | 274 | 442 | 373 | 413 | 300.96 | -0.74 | 7.49E-05 | 0.008838638 |
| Kif20a | 574 | 376 | 530 | 650 | 1064 | 883 | 891 | 699.70 | -0.73 | 0.000142136 | 0.014270352 |
| Fzd5 | 667 | 432 | 680 | 770 | 1081 | 1107 | 1229 | 839.06 | -0.73 | 0.000146284 | 0.014371574 |
| Slc25a34 | 623 | 411 | 504 | 947 | 1071 | 839 | 1312 | 786.09 | -0.73 | 2.55E-05 | 0.004022086 |
| 6530403H02Rik | 1626 | 1329 | 1669 | 2839 | 3599 | 2827 | 3302 | 2395.57 | -0.72 | 7.28E-05 | 0.008772516 |
| Abcc12 | 407 | 366 | 405 | 730 | 913 | 688 | 896 | 613.46 | -0.72 | 0.000280372 | 0.022560489 |
| Sox4 | 4576 | 2005 | 3066 | 4853 | 7045 | 5875 | 5453 | 4558.90 | -0.72 | 0.000166889 | 0.015592865 |
| Spc24 | 358 | 241 | 249 | 421 | 552 | 536 | 556 | 406.45 | -0.72 | 3.27E-07 | 0.000122822 |
| Hist1h1d | 5773 | 3253 | 3790 | 8249 | 11154 | 7099 | 7834 | 6497.44 | -0.70 | 0.000595951 | 0.035159592 |
| Pop1 | 1401 | 730 | 810 | 1804 | 1680 | 1923 | 2330 | 1460.73 | -0.70 | 5.83E-06 | 0.001334084 |
| Slc25a37 | 1441 | 1219 | 1224 | 1949 | 2247 | 2525 | 2832 | 1882.63 | -0.70 | 1.08E-06 | 0.000333444 |
| Gm16323 | 183 | 118 | 120 | 250 | 233 | 266 | 343 | 208.24 | -0.68 | 0.00046187 | 0.029971704 |
| Fam69c | 643 | 427 | 398 | 847 | 965 | 886 | 1039 | 719.61 | -0.68 | 1.88E-07 | 7.30E-05 |
| Dbpht2 | 7836 | 4164 | 4772 | 13489 | 12167 | 11351 | 12556 | 9004.02 | -0.68 | 0.00072841 | 0.040178415 |
| Hist1h2ak | 742 | 647 | 671 | 1010 | 1534 | 1064 | 1360 | 988.75 | -0.68 | 0.000361281 | 0.025844034 |
| Hist2h2bb | 2758 | 1710 | 1963 | 3367 | 5276 | 3333 | 3537 | 3061.54 | -0.67 | 0.000750015 | 0.040826339 |
| Pcdhb8 | 449 | 300 | 264 | 603 | 484 | 695 | 824 | 497.78 | -0.67 | 0.000993359 | 0.049112294 |
| Phex | 1197 | 579 | 708 | 1510 | 1758 | 1429 | 1622 | 1208.05 | -0.66 | 8.55E-05 | 0.00981353 |
| Ecel1 | 1824 | 976 | 1382 | 2292 | 2817 | 2202 | 2972 | 1999.42 | -0.66 | 1.31E-05 | 0.00249221 |
| Mcm3 | 740 | 459 | 621 | 1011 | 1369 | 1059 | 1036 | 877.83 | -0.65 | 0.000521573 | 0.032181495 |
| 3222401L13Rik | 326 | 256 | 289 | 366 | 510 | 524 | 533 | 396.47 | -0.65 | 0.00045688 | 0.029971704 |
| Mcm5 | 1372 | 868 | 1060 | 1964 | 2435 | 1875 | 2068 | 1614.51 | -0.64 | 4.68E-05 | 0.006418479 |
| Scg5 | 21523 | 13437 | 12150 | 31379 | 25241 | 30083 | 38143 | 23497.90 | -0.63 | 0.000206663 | 0.018125332 |
| Sncaip | 805 | 532 | 712 | 909 | 1214 | 1152 | 1311 | 932.05 | -0.63 | 4.31E-05 | 0.005939523 |
| Rfx4 | 607 | 367 | 412 | 905 | 803 | 1025 | 881 | 689.13 | -0.63 | 0.000471186 | 0.030277945 |
| Myh7 | 2130 | 1443 | 1981 | 3392 | 2964 | 3347 | 4537 | 2734.53 | -0.62 | 0.000380006 | 0.026683175 |
| Mycn | 760 | 442 | 462 | 649 | 964 | 924 | 927 | 719.70 | -0.61 | 0.000287177 | 0.022985208 |
| Tmem181a | 10500 | 7531 | 6947 | 16830 | 16176 | 14391 | 18831 | 12536.82 | -0.61 | 4.02E-05 | 0.005608421 |
| Racgap1 | 1843 | 1255 | 1418 | 2291 | 3087 | 2328 | 2680 | 2083.47 | -0.59 | 4.99E-05 | 0.006643727 |
| Dlx6os1 | 7125 | 5686 | 6548 | 12078 | 12880 | 11709 | 12255 | 9509.28 | -0.58 | 0.000174038 | 0.016064094 |
| 1700086L19Rik | 3020 | 1781 | 2263 | 4976 | 4580 | 4059 | 5046 | 3524.61 | -0.57 | 0.000459224 | 0.029971704 |
| Cbx2 | 410 | 261 | 307 | 479 | 609 | 535 | 560 | 442.07 | -0.57 | 0.000180038 | 0.016419302 |
| Dll1 | 1095 | 848 | 1088 | 1817 | 1806 | 1738 | 2127 | 1464.13 | -0.56 | 0.000488724 | 0.030904374 |
| Gm13889 | 1864 | 968 | 1131 | 2473 | 2114 | 2231 | 2920 | 1873.73 | -0.55 | 0.000310977 | 0.023807958 |
| Ehbp1l1 | 4109 | 2821 | 2945 | 7010 | 6329 | 5469 | 7167 | 4918.31 | -0.55 | 0.000607931 | 0.035682448 |
| Hmgcs2 | 1062 | 548 | 878 | 1297 | 1339 | 1427 | 1572 | 1128.03 | -0.55 | 0.00074337 | 0.040754187 |
| Khdrbs2 | 1202 | 745 | 742 | 1222 | 1680 | 1201 | 1630 | 1174.97 | -0.54 | 0.000617977 | 0.035995218 |
| Arx | 813 | 519 | 620 | 1141 | 1152 | 1155 | 1188 | 914.15 | -0.54 | 3.03E-05 | 0.004602063 |
| Kitl | 4510 | 2409 | 3362 | 6093 | 5474 | 6502 | 6147 | 4767.78 | -0.52 | 0.000535885 | 0.032624845 |
| Pcdh17 | 16311 | 8571 | 9033 | 18689 | 21476 | 18509 | 17713 | 15250.87 | -0.52 | 0.000827958 | 0.043634697 |
| 2810459M11Rik | 1986 | 1054 | 1140 | 1705 | 2232 | 2081 | 2350 | 1751.84 | -0.52 | 0.000466482 | 0.030079562 |
| Kptn | 2797 | 1737 | 1946 | 4531 | 4123 | 3567 | 4338 | 3159.94 | -0.51 | 0.000991369 | 0.049112294 |
| Rnf144a | 3713 | 2716 | 2210 | 4452 | 5010 | 4675 | 5043 | 3880.90 | -0.51 | 0.000113821 | 0.012262654 |
| Slx1b | 993 | 597 | 570 | 1131 | 1247 | 1058 | 1255 | 949.87 | -0.47 | 0.000338497 | 0.024914892 |
| Cldn5 | 2631 | 1898 | 1984 | 3423 | 3835 | 3171 | 3905 | 2910.82 | -0.47 | 5.30E-05 | 0.007009145 |
| Cyp2d22 | 2566 | 1414 | 1647 | 3296 | 2687 | 3122 | 3764 | 2544.54 | -0.47 | 0.000534404 | 0.032624845 |
| Btg1 | 5960 | 3465 | 4472 | 7212 | 8204 | 7064 | 7489 | 6113.66 | -0.46 | 0.000308696 | 0.023750519 |
| Serpine2 | 21886 | 12460 | 16317 | 30067 | 30952 | 24371 | 30797 | 23055.45 | -0.46 | 0.000993187 | 0.049112294 |
| 9430015G10Rik | 2014 | 1172 | 1320 | 2566 | 2096 | 2359 | 3097 | 2015.11 | -0.45 | 0.000929956 | 0.047242723 |
| Gm3764 | 3987 | 2905 | 3103 | 4966 | 4571 | 5531 | 5968 | 4345.69 | -0.43 | 0.000518235 | 0.032061963 |
| Sox2 | 4571 | 2354 | 3132 | 4810 | 5149 | 4897 | 5646 | 4250.14 | -0.43 | 0.000454871 | 0.029971704 |
| Ankrd6 | 5490 | 3246 | 4204 | 7546 | 6963 | 6481 | 7831 | 5780.19 | -0.41 | 0.000433837 | 0.02916669 |
| Pak6 | 2038 | 1496 | 1608 | 2493 | 2481 | 2620 | 2997 | 2206.94 | -0.41 | 0.000342345 | 0.024957385 |
| Pmm2 | 1733 | 1114 | 1044 | 2211 | 1918 | 2029 | 2331 | 1712.84 | -0.40 | 0.000560157 | 0.033655001 |
| Hjurp | 6545 | 4604 | 4762 | 8227 | 8295 | 7961 | 8902 | 6888.75 | -0.40 | 9.17E-06 | 0.001942828 |
| Mettl3 | 3417 | 2166 | 2294 | 4148 | 3943 | 3703 | 4463 | 3356.02 | -0.36 | 2.24E-05 | 0.003766199 |
| Akt2 | 15469 | 10090 | 11120 | 21231 | 17849 | 17372 | 21127 | 15841.89 | -0.31 | 0.000882665 | 0.045377461 |
| Ccdc41os1 | 4236 | 2766 | 2608 | 5686 | 2693 | 2838 | 3418 | 3364.64 | 0.41 | 0.000592673 | 0.03505651 |
| Socs4 | 2585 | 1812 | 2008 | 3368 | 1827 | 2012 | 1891 | 2185.49 | 0.43 | 0.000465883 | 0.030079562 |
| Scn1a | 30986 | 22629 | 22352 | 37320 | 22351 | 20138 | 24011 | 25404.35 | 0.44 | 1.70E-05 | 0.00308592 |
| Kcnip4 | 6159 | 4328 | 4525 | 6537 | 4527 | 3692 | 4469 | 4868.72 | 0.45 | 0.000924976 | 0.047157312 |
| Kcnab2 | 35546 | 21021 | 21523 | 35008 | 21624 | 19963 | 23953 | 25129.96 | 0.45 | 3.89E-05 | 0.00549634 |
| Slc3a2 | 11118 | 6509 | 7860 | 10408 | 7159 | 6137 | 7667 | 8060.05 | 0.46 | 0.00101801 | 0.049687129 |
| Itih3 | 4012 | 2368 | 3219 | 5796 | 2937 | 2607 | 2949 | 3324.57 | 0.49 | 0.000457949 | 0.029971704 |
| Nudt19 | 7648 | 4705 | 4764 | 10431 | 5495 | 4300 | 5264 | 5903.85 | 0.50 | 0.000356296 | 0.025567304 |
| 4930452B06Rik | 936 | 593 | 611 | 990 | 628 | 532 | 593 | 689.29 | 0.50 | 0.000347067 | 0.0251415 |
| Pid1 | 4238 | 2475 | 2946 | 3703 | 2511 | 2440 | 2595 | 2977.13 | 0.51 | 0.000935323 | 0.047368338 |
| Npas3 | 1286 | 774 | 1037 | 1406 | 829 | 746 | 923 | 990.84 | 0.53 | 0.000240194 | 0.02005545 |
| Gm4070 | 1676 | 1246 | 1289 | 2100 | 1018 | 1078 | 1415 | 1387.42 | 0.53 | 6.70E-05 | 0.008298919 |
| Dzip1 | 2745 | 2067 | 2177 | 3055 | 1830 | 1627 | 2147 | 2231.50 | 0.54 | 0.000128627 | 0.013223203 |
| Ndrg2 | 67612 | 48858 | 59222 | 91986 | 53090 | 40699 | 52332 | 58490.14 | 0.54 | 0.000122862 | 0.01296049 |
| Gm14416 | 490 | 334 | 426 | 615 | 314 | 338 | 367 | 408.55 | 0.54 | 0.000585465 | 0.03471987 |
| Acadl | 2670 | 1262 | 1846 | 2666 | 1542 | 1411 | 1582 | 1819.45 | 0.55 | 0.000394855 | 0.027307061 |
| Gstm7 | 1303 | 758 | 986 | 1300 | 783 | 806 | 768 | 952.17 | 0.55 | 0.000428937 | 0.029135873 |
| Kcnj16 | 1675 | 1039 | 1107 | 1592 | 951 | 865 | 1081 | 1178.36 | 0.58 | 2.28E-05 | 0.003805933 |
| Vwc2l | 1024 | 499 | 732 | 1019 | 535 | 567 | 623 | 702.54 | 0.58 | 0.000378657 | 0.026670243 |
| Stk3 | 3050 | 1965 | 2120 | 2612 | 1873 | 1572 | 1797 | 2147.57 | 0.59 | 0.00061065 | 0.03575034 |
| St8sia1 | 3921 | 2085 | 1729 | 3739 | 1817 | 2045 | 2015 | 2413.49 | 0.59 | 0.00062024 | 0.036015873 |
| Slc14a1 | 1372 | 663 | 815 | 1196 | 725 | 607 | 771 | 863.46 | 0.60 | 0.000305003 | 0.023667209 |
| Anxa5 | 8094 | 3902 | 5516 | 7761 | 4333 | 4089 | 4687 | 5393.55 | 0.60 | 3.16E-05 | 0.004665337 |
| Gm26880 | 3617 | 2705 | 2311 | 3020 | 2038 | 1946 | 2221 | 2572.48 | 0.62 | 0.000973817 | 0.048885188 |
| Gabra1 | 25914 | 22766 | 17681 | 30842 | 18970 | 14407 | 17576 | 21160.38 | 0.62 | 0.000399608 | 0.02755246 |
| Cadps | 36918 | 19934 | 16931 | 30022 | 18230 | 16315 | 18455 | 22036.28 | 0.62 | 0.000334387 | 0.024914892 |
| Apold1 | 1995 | 1085 | 1506 | 1931 | 934 | 1120 | 1304 | 1398.11 | 0.64 | 0.00012601 | 0.013223203 |
| Slc4a10 | 51815 | 25343 | 31585 | 37505 | 24490 | 22697 | 28254 | 31467.65 | 0.64 | 0.00048536 | 0.030904374 |
| Ell3 | 352 | 195 | 196 | 312 | 165 | 167 | 204 | 224.00 | 0.64 | 0.001004113 | 0.049398356 |
| Slc24a5 | 827 | 515 | 530 | 662 | 415 | 424 | 470 | 550.10 | 0.65 | 0.000640355 | 0.036653025 |
| Grin3a | 3151 | 1522 | 1932 | 2356 | 1452 | 1541 | 1566 | 1918.55 | 0.65 | 0.000497525 | 0.031202303 |
| Nefm | 11278 | 12376 | 12105 | 17739 | 8000 | 9215 | 10649 | 11716.07 | 0.66 | 0.000983612 | 0.049112294 |
| Phyh | 8589 | 4330 | 5171 | 6606 | 4852 | 3347 | 4264 | 5271.11 | 0.66 | 0.000864837 | 0.045095651 |
| Arhgap22 | 705 | 416 | 425 | 765 | 447 | 279 | 424 | 484.73 | 0.66 | 0.000655941 | 0.037444259 |
| Khk | 1016 | 737 | 858 | 1130 | 678 | 545 | 687 | 808.41 | 0.66 | 5.42E-05 | 0.007131002 |
| Pex5l | 27350 | 17930 | 16990 | 33137 | 18447 | 13007 | 15223 | 19929.59 | 0.67 | 1.37E-05 | 0.002565535 |
| Cd72 | 420 | 418 | 344 | 733 | 326 | 281 | 331 | 402.70 | 0.69 | 0.00088287 | 0.045377461 |
| Gm5148 | 594 | 458 | 519 | 735 | 281 | 405 | 465 | 492.84 | 0.70 | 0.000552194 | 0.033351624 |
| B2m | 7353 | 3969 | 5089 | 5768 | 4392 | 2839 | 3780 | 4743.82 | 0.70 | 0.000987801 | 0.049112294 |
| Gm17322 | 1198 | 991 | 604 | 1696 | 694 | 671 | 790 | 927.10 | 0.70 | 0.000829195 | 0.043634697 |
| F2r | 1727 | 677 | 1181 | 1680 | 853 | 803 | 853 | 1084.92 | 0.70 | 0.00043259 | 0.02916669 |
| Alox8 | 380 | 382 | 395 | 563 | 291 | 267 | 296 | 370.85 | 0.71 | 0.000841798 | 0.044095192 |
| Cd38 | 2417 | 966 | 1517 | 2348 | 1184 | 991 | 1257 | 1484.23 | 0.71 | 0.000129396 | 0.013223203 |
| Kcnab3 | 915 | 805 | 745 | 1041 | 580 | 551 | 595 | 754.66 | 0.73 | 4.97E-05 | 0.006643727 |
| Gm20459 | 2760 | 1175 | 1269 | 2119 | 1207 | 1051 | 1145 | 1497.12 | 0.74 | 0.000179687 | 0.016419302 |
| Vamp1 | 6553 | 5110 | 6190 | 5911 | 3117 | 3978 | 4703 | 5183.28 | 0.75 | 0.000781808 | 0.04201027 |
| Aldh1a1 | 11618 | 4688 | 5503 | 10454 | 5747 | 3903 | 5006 | 6483.72 | 0.75 | 0.000240935 | 0.02005545 |
| AW495222 | 208 | 187 | 172 | 257 | 132 | 126 | 135 | 175.05 | 0.76 | 0.0006258 | 0.036174718 |
| 3632451O06Rik | 5063 | 2973 | 2954 | 4120 | 2278 | 2256 | 2546 | 3158.74 | 0.77 | 9.80E-07 | 0.000311679 |
| Pls3 | 19716 | 8511 | 8523 | 14113 | 8292 | 6873 | 7907 | 10339.86 | 0.78 | 0.000128792 | 0.013223203 |
| Sst | 2519 | 2611 | 2350 | 2622 | 1764 | 1268 | 1800 | 2193.32 | 0.81 | 0.000745811 | 0.040754187 |
| Itga3 | 1660 | 635 | 916 | 1077 | 595 | 610 | 725 | 876.47 | 0.82 | 0.000460096 | 0.029971704 |
| Sh3gl2 | 11864 | 9060 | 6265 | 10478 | 5903 | 5256 | 5999 | 7861.09 | 0.83 | 2.20E-05 | 0.003726421 |
| Ace | 5593 | 2033 | 3635 | 4004 | 2607 | 2038 | 2099 | 3112.03 | 0.83 | 0.000726964 | 0.040178415 |
| Zmat4 | 6725 | 2948 | 3913 | 4566 | 3441 | 2126 | 2488 | 3724.04 | 0.84 | 0.000721607 | 0.040178415 |
| Kcns3 | 694 | 345 | 551 | 512 | 342 | 270 | 340 | 439.40 | 0.84 | 0.000762107 | 0.041313984 |
| Adcy8 | 2561 | 1000 | 1273 | 1559 | 960 | 832 | 1016 | 1297.36 | 0.85 | 0.000316145 | 0.023961042 |
| Disp2 | 13153 | 6218 | 4734 | 8268 | 3948 | 4886 | 5298 | 6531.79 | 0.85 | 0.000488631 | 0.030904374 |
| Ndufa12 | 10196 | 6489 | 8516 | 13360 | 6285 | 4523 | 6085 | 7817.08 | 0.85 | 2.14E-08 | 1.07E-05 |
| Klf4 | 499 | 265 | 339 | 690 | 182 | 252 | 336 | 353.01 | 0.85 | 0.000146269 | 0.014371574 |
| Trim66 | 1488 | 1016 | 651 | 1133 | 707 | 547 | 653 | 885.34 | 0.85 | 0.000448072 | 0.029729909 |
| Ide | 12562 | 10406 | 11822 | 13154 | 9575 | 5473 | 6438 | 10120.38 | 0.87 | 0.000426572 | 0.029061481 |
| Fam13a | 2007 | 1197 | 1479 | 1988 | 1043 | 930 | 927 | 1364.09 | 0.87 | 2.06E-08 | 1.05E-05 |
| Gm10524 | 764 | 270 | 366 | 716 | 306 | 268 | 302 | 410.58 | 0.88 | 0.000143193 | 0.014313692 |
| Gm26822 | 1142 | 1028 | 950 | 1022 | 539 | 517 | 850 | 883.96 | 0.88 | 0.000485969 | 0.030904374 |
| Faah | 6014 | 2891 | 2066 | 4261 | 2177 | 1976 | 2290 | 3027.40 | 0.88 | 0.00017029 | 0.015781777 |
| Myo5c | 1608 | 894 | 1359 | 1114 | 791 | 624 | 779 | 1042.84 | 0.90 | 0.000373445 | 0.02645642 |
| Gm15446 | 5346 | 3722 | 3736 | 7021 | 2863 | 2589 | 2862 | 3958.74 | 0.90 | 4.05E-18 | 1.03E-14 |
| Gm22988 | 4330 | 4357 | 3582 | 8981 | 2961 | 3257 | 2471 | 4194.05 | 0.91 | 7.40E-05 | 0.008827863 |
| Tep1 | 860 | 564 | 599 | 897 | 466 | 374 | 388 | 591.00 | 0.92 | 5.13E-08 | 2.35E-05 |
| Llgl2 | 333 | 204 | 210 | 237 | 130 | 141 | 150 | 202.72 | 0.93 | 0.000233702 | 0.019740507 |
| Aspg | 225 | 120 | 99 | 217 | 104 | 83 | 79 | 129.27 | 0.94 | 0.000639123 | 0.036653025 |
| Gm2115 | 2360 | 774 | 1135 | 2660 | 930 | 776 | 985 | 1300.93 | 0.94 | 7.76E-05 | 0.009016688 |
| Pkdcc | 674 | 283 | 386 | 378 | 237 | 247 | 230 | 348.49 | 0.95 | 0.000733182 | 0.040344414 |
| Cpeb1 | 2151 | 1721 | 1378 | 2093 | 1006 | 979 | 1091 | 1500.42 | 0.95 | 4.80E-08 | 2.24E-05 |
| Tmem25 | 2950 | 1594 | 1409 | 1756 | 1077 | 900 | 1231 | 1562.29 | 0.96 | 5.99E-05 | 0.007698684 |
| Cyp11a1 | 513 | 362 | 201 | 495 | 221 | 181 | 229 | 310.32 | 0.97 | 0.000189021 | 0.017102287 |
| Gm2004 | 851 | 494 | 788 | 792 | 465 | 288 | 458 | 596.00 | 0.98 | 8.98E-05 | 0.010229495 |
| Gm14150 | 2305 | 1237 | 1207 | 2503 | 1027 | 747 | 1032 | 1397.86 | 1.00 | 1.04E-09 | 8.17E-07 |
| Mast1 | 1978 | 1132 | 739 | 1100 | 609 | 683 | 686 | 992.20 | 1.01 | 0.000328952 | 0.024769887 |
| Gm24497 | 8674 | 13177 | 7274 | 14919 | 4308 | 6768 | 6857 | 9020.92 | 1.02 | 0.000509569 | 0.031783487 |
| AF529169 | 499 | 290 | 315 | 399 | 190 | 175 | 215 | 298.22 | 1.06 | 2.56E-07 | 9.77E-05 |
| Klf2 | 316 | 151 | 256 | 350 | 88 | 155 | 162 | 207.89 | 1.07 | 0.000126666 | 0.013223203 |
| Cmtm8 | 266 | 103 | 209 | 200 | 86 | 104 | 107 | 153.40 | 1.07 | 0.000615009 | 0.035913728 |
| Sgk1 | 40313 | 10143 | 19370 | 41712 | 15394 | 10555 | 13284 | 20323.90 | 1.07 | 0.000193386 | 0.017292194 |
| Chst8 | 878 | 308 | 439 | 574 | 261 | 242 | 315 | 422.50 | 1.08 | 2.46E-05 | 0.003989669 |
| Efr3a | 16744 | 9834 | 6708 | 8908 | 6402 | 4700 | 4940 | 8403.58 | 1.08 | 0.000157224 | 0.014953646 |
| 1810041L15Rik | 5368 | 1863 | 2786 | 2351 | 1490 | 1641 | 1503 | 2432.41 | 1.09 | 0.000312297 | 0.023829297 |
| Fzd4 | 1210 | 420 | 1007 | 857 | 425 | 415 | 475 | 688.05 | 1.09 | 0.000192505 | 0.017280947 |
| Gm24245 | 10686 | 12071 | 15361 | 13659 | 6366 | 6648 | 7516 | 10763.93 | 1.10 | 3.06E-05 | 0.004602063 |
| Gm4793 | 121 | 92 | 95 | 141 | 51 | 58 | 58 | 88.30 | 1.11 | 2.49E-05 | 0.003990652 |
| Cbln4 | 171 | 150 | 102 | 127 | 66 | 74 | 63 | 110.91 | 1.16 | 0.000720195 | 0.040178415 |
| Gpr64 | 156 | 209 | 207 | 298 | 83 | 119 | 115 | 173.14 | 1.17 | 0.000208018 | 0.01817455 |
| Grem1 | 357 | 353 | 431 | 792 | 221 | 193 | 264 | 368.24 | 1.17 | 2.53E-06 | 0.000679015 |
| Kcnmb2 | 386 | 154 | 255 | 204 | 132 | 81 | 144 | 195.25 | 1.19 | 0.000710866 | 0.039981406 |
| Nefh | 5284 | 2060 | 5033 | 3037 | 1761 | 1670 | 2163 | 3069.53 | 1.19 | 0.000226172 | 0.019318266 |
| Robo3 | 1473 | 917 | 309 | 1098 | 386 | 357 | 546 | 711.43 | 1.21 | 0.00084811 | 0.044324418 |
| Gm5860 | 413 | 342 | 481 | 292 | 133 | 205 | 230 | 315.09 | 1.21 | 0.00069862 | 0.039486717 |
| Tdrd5 | 223 | 150 | 167 | 151 | 79 | 73 | 95 | 137.55 | 1.22 | 7.22E-05 | 0.008766237 |
| Ifi27 | 7841 | 1732 | 4944 | 3744 | 2105 | 1986 | 2108 | 3470.34 | 1.22 | 0.000568374 | 0.033970365 |
| Pparg | 192 | 130 | 121 | 132 | 87 | 50 | 64 | 113.26 | 1.23 | 0.000351303 | 0.025288328 |
| Adcy1 | 91711 | 40587 | 21886 | 46094 | 29650 | 17227 | 19420 | 37457.19 | 1.23 | 0.000491646 | 0.031003506 |
| Ccdc136 | 3267 | 1322 | 2450 | 1420 | 1177 | 858 | 926 | 1674.47 | 1.23 | 0.000317164 | 0.023961042 |
| Spp1 | 1205 | 434 | 1309 | 1093 | 290 | 501 | 598 | 778.74 | 1.24 | 0.00043166 | 0.02916669 |
| Phactr2 | 4482 | 1364 | 3123 | 1666 | 1146 | 1273 | 1242 | 2078.87 | 1.25 | 0.000718155 | 0.040178415 |
| Gpr123 | 3965 | 1922 | 1857 | 1166 | 907 | 1114 | 1059 | 1760.36 | 1.26 | 0.000705852 | 0.039797162 |
| Nrip3 | 19688 | 4960 | 7959 | 6320 | 4998 | 3616 | 4156 | 7316.79 | 1.26 | 0.000680946 | 0.038774945 |
| Ecm2 | 2246 | 629 | 1427 | 763 | 544 | 548 | 621 | 980.80 | 1.27 | 0.001005619 | 0.049398356 |
| St8sia5 | 1662 | 1261 | 524 | 1085 | 545 | 473 | 504 | 875.22 | 1.27 | 0.000105866 | 0.011699824 |
| Hunk | 2815 | 792 | 572 | 1619 | 618 | 537 | 567 | 1019.13 | 1.32 | 0.000212139 | 0.018464134 |
| Tuba1c | 3298 | 1041 | 2002 | 1216 | 913 | 741 | 769 | 1445.55 | 1.34 | 0.000202169 | 0.017868174 |
| Sertm1 | 2435 | 562 | 511 | 1260 | 506 | 424 | 464 | 832.38 | 1.34 | 0.000373333 | 0.02645642 |
| C1ra | 288 | 136 | 165 | 116 | 83 | 77 | 66 | 136.45 | 1.35 | 0.000621479 | 0.036015873 |
| Gm26794 | 860 | 351 | 189 | 381 | 179 | 167 | 194 | 325.29 | 1.36 | 0.000374465 | 0.02645642 |
| Klhl33 | 466 | 560 | 476 | 982 | 311 | 218 | 233 | 463.45 | 1.37 | 4.02E-07 | 0.000139425 |
| Shank1 | 5532 | 3055 | 1025 | 2746 | 1298 | 1123 | 1349 | 2286.25 | 1.38 | 0.000339841 | 0.02493365 |
| Lgals1 | 2697 | 653 | 1065 | 663 | 500 | 529 | 511 | 942.65 | 1.39 | 0.001023903 | 0.049868412 |
| Cdkn1c | 3444 | 668 | 1935 | 1149 | 686 | 723 | 782 | 1337.68 | 1.39 | 0.000763436 | 0.041313984 |
| M5C1000I18Rik | 613 | 192 | 130 | 358 | 123 | 103 | 146 | 226.53 | 1.39 | 0.000278475 | 0.022560489 |
| Pvalb | 2757 | 1808 | 4154 | 2019 | 1191 | 1054 | 1227 | 2149.34 | 1.41 | 7.75E-05 | 0.009016688 |
| Hkdc1 | 341 | 268 | 94 | 222 | 103 | 79 | 98 | 174.36 | 1.42 | 0.000439459 | 0.02932842 |
| Steap2 | 6248 | 1656 | 4688 | 2346 | 1494 | 1426 | 1634 | 2836.58 | 1.42 | 0.000232541 | 0.01971513 |
| Vsnl1 | 11278 | 5413 | 3058 | 4884 | 2711 | 2307 | 2228 | 4549.59 | 1.43 | 2.33E-05 | 0.003841314 |
| Slc16a12 | 1609 | 390 | 1087 | 613 | 362 | 325 | 424 | 693.25 | 1.43 | 0.000315624 | 0.023961042 |
| Chrm3 | 3370 | 2143 | 714 | 1887 | 1004 | 622 | 761 | 1501.09 | 1.43 | 0.000219146 | 0.018930085 |
| Ppm1e | 31362 | 8442 | 7090 | 13228 | 6313 | 4835 | 5651 | 10579.90 | 1.44 | 5.69E-05 | 0.007384428 |
| Cab39l | 16121 | 3457 | 9222 | 5795 | 3544 | 2987 | 3551 | 6377.21 | 1.45 | 0.000152823 | 0.014698663 |
| Pip5k1b | 4156 | 820 | 1404 | 1200 | 795 | 653 | 667 | 1356.13 | 1.47 | 0.000401484 | 0.027598719 |
| Tmem132c | 756 | 487 | 722 | 401 | 142 | 309 | 278 | 464.58 | 1.47 | 0.000148909 | 0.014443567 |
| Cpne7 | 3799 | 661 | 1728 | 1581 | 668 | 728 | 754 | 1382.66 | 1.48 | 0.000119806 | 0.012696696 |
| Gfra2 | 2019 | 1382 | 607 | 686 | 587 | 390 | 401 | 898.80 | 1.49 | 0.000435761 | 0.02916669 |
| Sgcg | 164 | 141 | 236 | 248 | 67 | 103 | 54 | 148.65 | 1.51 | 4.94E-05 | 0.006643727 |
| Cacna1g | 4378 | 1642 | 1614 | 1166 | 619 | 800 | 1072 | 1628.07 | 1.53 | 0.000108519 | 0.011829036 |
| Tmem200a | 1285 | 219 | 185 | 820 | 186 | 202 | 224 | 407.38 | 1.55 | 0.000866847 | 0.045097697 |
| Kcnj3 | 13923 | 7547 | 4469 | 5691 | 3245 | 2615 | 2810 | 5839.17 | 1.56 | 3.18E-06 | 0.000831904 |
| Cgnl1 | 11099 | 2914 | 8813 | 4748 | 2315 | 2325 | 2929 | 5108.52 | 1.58 | 2.73E-05 | 0.004250655 |
| Adamts1 | 7135 | 2096 | 4199 | 2043 | 1540 | 1206 | 1479 | 2869.31 | 1.58 | 6.32E-05 | 0.008009527 |
| Cerkl | 219 | 71 | 112 | 82 | 44 | 40 | 46 | 88.34 | 1.58 | 0.000293068 | 0.023194012 |
| Pon3 | 887 | 158 | 535 | 253 | 155 | 156 | 176 | 333.43 | 1.60 | 0.000877178 | 0.045377461 |
| Gm13446 | 129 | 96 | 66 | 66 | 49 | 22 | 26 | 67.52 | 1.61 | 0.000984837 | 0.049112294 |
| Slc37a2 | 2716 | 575 | 1669 | 819 | 482 | 469 | 571 | 1053.07 | 1.62 | 0.000154128 | 0.014762078 |
| Itga8 | 3130 | 473 | 953 | 918 | 390 | 430 | 474 | 933.11 | 1.69 | 0.000134886 | 0.013722991 |
| Ptgs2 | 1010 | 263 | 161 | 317 | 152 | 127 | 129 | 297.25 | 1.70 | 0.000341001 | 0.024938816 |
| Slc29a4 | 676 | 137 | 525 | 221 | 112 | 131 | 148 | 284.45 | 1.71 | 0.000441955 | 0.029409281 |
| Pm20d1 | 253 | 92 | 268 | 231 | 67 | 58 | 82 | 151.27 | 1.72 | 3.27E-06 | 0.000840726 |
| Ifi27l2a | 230 | 84 | 160 | 193 | 51 | 59 | 48 | 116.66 | 1.72 | 8.51E-08 | 3.48E-05 |
| Htr1a | 1706 | 511 | 223 | 742 | 288 | 216 | 216 | 532.29 | 1.72 | 0.000158183 | 0.014962634 |
| Marveld2 | 514 | 97 | 176 | 251 | 75 | 80 | 82 | 174.52 | 1.73 | 2.95E-05 | 0.004538666 |
| Stac2 | 869 | 531 | 183 | 340 | 145 | 153 | 174 | 347.32 | 1.73 | 7.49E-05 | 0.008838638 |
| Sidt1 | 2476 | 1239 | 343 | 992 | 493 | 330 | 371 | 885.74 | 1.73 | 0.000175551 | 0.016138721 |
| Rad51ap2 | 160 | 162 | 82 | 84 | 54 | 30 | 40 | 92.88 | 1.75 | 0.000256463 | 0.021041899 |
| Eps8l1 | 749 | 266 | 198 | 152 | 108 | 81 | 136 | 242.69 | 1.77 | 0.000362554 | 0.025854304 |
| Tc2n | 1359 | 373 | 804 | 389 | 158 | 249 | 294 | 527.20 | 1.78 | 4.97E-05 | 0.006643727 |
| Cbln1 | 914 | 449 | 329 | 152 | 118 | 160 | 167 | 340.23 | 1.80 | 0.000404784 | 0.027742274 |
| Fhad1 | 1982 | 485 | 1012 | 342 | 297 | 316 | 272 | 687.40 | 1.81 | 0.000252986 | 0.02090649 |
| Crhbp | 1099 | 669 | 224 | 692 | 275 | 140 | 155 | 461.97 | 1.88 | 1.04E-05 | 0.002107264 |
| Hapln4 | 3226 | 1863 | 1445 | 1342 | 620 | 495 | 607 | 1413.10 | 1.92 | 2.38E-09 | 1.65E-06 |
| Zfp804b | 307 | 133 | 137 | 95 | 49 | 48 | 47 | 119.58 | 1.93 | 1.37E-05 | 0.002565535 |
| Msx1 | 792 | 114 | 437 | 114 | 100 | 103 | 96 | 254.96 | 1.97 | 0.000972105 | 0.048885188 |
| Fgf10 | 625 | 255 | 272 | 127 | 85 | 89 | 92 | 228.32 | 1.99 | 3.26E-05 | 0.004788985 |
| Grp | 117 | 139 | 40 | 75 | 38 | 21 | 19 | 68.11 | 2.00 | 0.000462191 | 0.029971704 |
| Lefty1 | 516 | 57 | 55 | 427 | 68 | 51 | 61 | 155.27 | 2.00 | 0.000984269 | 0.049112294 |
| Rtn4r | 677 | 399 | 114 | 208 | 134 | 66 | 77 | 244.58 | 2.03 | 0.000169163 | 0.015741069 |
| Fam19a1 | 2969 | 422 | 411 | 1086 | 234 | 278 | 326 | 758.58 | 2.09 | 2.07E-05 | 0.003539649 |
| Plek2 | 353 | 60 | 182 | 93 | 45 | 43 | 39 | 116.69 | 2.10 | 0.000110745 | 0.012014548 |
| Slc16a8 | 1085 | 157 | 533 | 298 | 125 | 104 | 149 | 347.36 | 2.12 | 1.92E-05 | 0.003390101 |
| Kcng4 | 1251 | 520 | 908 | 248 | 155 | 220 | 193 | 529.94 | 2.13 | 1.29E-05 | 0.00249221 |
| Igfn1 | 293 | 181 | 134 | 47 | 39 | 43 | 41 | 118.87 | 2.19 | 0.000148859 | 0.014443567 |
| Bdnf | 3180 | 944 | 470 | 644 | 264 | 289 | 334 | 859.82 | 2.20 | 0.001012469 | 0.049522291 |
| Gm7224 | 26 | 22 | 27 | 65 | 5 | 11 | 7 | 22.51 | 2.21 | 0.00022192 | 0.019097638 |
| Cck | 10758 | 4869 | 1713 | 3440 | 1740 | 769 | 890 | 3454.72 | 2.26 | 0.00074597 | 0.040754187 |
| Lmx1a | 717 | 106 | 495 | 150 | 61 | 92 | 92 | 250.76 | 2.29 | 7.34E-05 | 0.008797019 |
| Gm11490 | 35 | 24 | 15 | 23 | 4 | 6 | 6 | 16.47 | 2.30 | 0.000535094 | 0.032624845 |
| Rab20 | 537 | 90 | 405 | 135 | 58 | 55 | 83 | 200.07 | 2.30 | 4.02E-05 | 0.005608421 |
| Kcnc2 | 12082 | 4288 | 6090 | 3724 | 1582 | 1238 | 1506 | 4460.41 | 2.30 | 1.69E-10 | 1.61E-07 |
| Ccnb1ip1 | 39 | 100 | 67 | 139 | 11 | 26 | 20 | 59.25 | 2.31 | 2.94E-05 | 0.004538666 |
| Steap4 | 399 | 54 | 264 | 39 | 41 | 40 | 43 | 130.19 | 2.33 | 0.000968078 | 0.048811199 |
| Adra1d | 885 | 127 | 113 | 270 | 59 | 73 | 70 | 212.69 | 2.34 | 2.65E-05 | 0.004151611 |
| Gm2223 | 104 | 330 | 211 | 78 | 79 | 34 | 20 | 143.75 | 2.34 | 0.000812195 | 0.043439154 |
| Gm14631 | 21 | 59 | 75 | 38 | 13 | 9 | 12 | 36.66 | 2.37 | 0.000575344 | 0.034208298 |
| Enpp2 | 597775 | 87070 | 399167 | 108659 | 65053 | 58937 | 64367 | 202544.75 | 2.37 | 0.000412064 | 0.028156905 |
| Alox12b | 108 | 70 | 16 | 56 | 13 | 15 | 10 | 41.42 | 2.38 | 0.000195048 | 0.01737289 |
| Wdr72 | 842 | 181 | 663 | 153 | 50 | 131 | 111 | 318.82 | 2.40 | 5.71E-05 | 0.007384428 |
| Cox8b | 448 | 40 | 221 | 69 | 38 | 40 | 36 | 127.52 | 2.43 | 0.000309189 | 0.023750519 |
| Tjp3 | 671 | 77 | 413 | 95 | 35 | 85 | 68 | 210.78 | 2.44 | 0.000265979 | 0.021744772 |
| Npr3 | 3607 | 823 | 2133 | 1061 | 340 | 384 | 409 | 1271.95 | 2.44 | 5.60E-09 | 3.66E-06 |
| Col4a4 | 448 | 32 | 309 | 76 | 34 | 50 | 44 | 144.67 | 2.45 | 0.000566395 | 0.033940679 |
| Slc26a4 | 443 | 98 | 31 | 74 | 35 | 22 | 28 | 100.01 | 2.52 | 0.000387384 | 0.02692233 |
| Rec8 | 78 | 79 | 291 | 340 | 30 | 42 | 35 | 128.94 | 2.55 | 3.65E-06 | 0.000919405 |
| Abhd1 | 812 | 537 | 687 | 855 | 141 | 110 | 121 | 474.30 | 2.64 | 1.24E-39 | 7.09E-36 |
| Calml4 | 3696 | 463 | 2026 | 613 | 237 | 306 | 325 | 1109.29 | 2.66 | 0.000525357 | 0.032327819 |
| A930018M24Rik | 376 | 468 | 482 | 978 | 109 | 77 | 102 | 369.51 | 2.66 | 5.09E-19 | 1.46E-15 |
| Cldn1 | 3274 | 276 | 1613 | 659 | 293 | 203 | 222 | 928.70 | 2.66 | 0.00082249 | 0.043620959 |
| 2310002F09Rik | 361 | 19 | 25 | 181 | 24 | 18 | 16 | 80.54 | 2.78 | 0.000388116 | 0.02692233 |
| Gm10462 | 61 | 92 | 90 | 93 | 29 | 11 | 0 | 57.77 | 2.78 | 0.001007894 | 0.049404056 |
| Tgtp1 | 1375 | 228 | 1247 | 233 | 150 | 105 | 116 | 521.64 | 2.80 | 0.000628558 | 0.03624264 |
| BC021767 | 57 | 10 | 29 | 17 | 2 | 4 | 7 | 17.96 | 2.81 | 0.000750858 | 0.040826339 |
| Nrn1 | 12569 | 3611 | 1817 | 1464 | 1058 | 568 | 534 | 3076.31 | 2.81 | 0.000820406 | 0.043620959 |
| Igfbp2 | 15840 | 1993 | 10103 | 2298 | 924 | 1168 | 1371 | 4945.83 | 2.84 | 0.000472204 | 0.030277945 |
| Crhr2 | 738 | 96 | 440 | 84 | 38 | 52 | 60 | 221.46 | 2.89 | 1.12E-05 | 0.0022186 |
| Kl | 7657 | 837 | 4723 | 1163 | 453 | 499 | 526 | 2316.23 | 2.98 | 0.000268217 | 0.021849654 |
| Wdr86 | 4093 | 273 | 1266 | 477 | 217 | 155 | 208 | 928.86 | 3.02 | 0.000640479 | 0.036653025 |
| Pon1 | 93 | 9 | 123 | 27 | 7 | 10 | 9 | 42.39 | 3.04 | 0.000336429 | 0.024914892 |
| A2m | 6167 | 532 | 2998 | 547 | 300 | 307 | 387 | 1621.46 | 3.05 | 0.000930779 | 0.047242723 |
| BC048943 | 57 | 16 | 39 | 8 | 3 | 4 | 5 | 19.93 | 3.08 | 0.000495761 | 0.031177104 |
| Ins2 | 82 | 14 | 79 | 20 | 8 | 7 | 3 | 32.16 | 3.15 | 0.000151926 | 0.014673984 |
| Prlr | 6883 | 654 | 3715 | 543 | 270 | 391 | 406 | 1876.79 | 3.17 | 0.000810414 | 0.043439154 |
| Krt8 | 1464 | 173 | 826 | 274 | 47 | 100 | 92 | 430.65 | 3.20 | 5.51E-05 | 0.007202752 |
| Krt18 | 2331 | 334 | 1450 | 316 | 85 | 156 | 139 | 709.51 | 3.25 | 7.70E-05 | 0.009016688 |
| Mageb16-ps1 | 23 | 9 | 18 | 23 | 1 | 1 | 4 | 11.16 | 3.28 | 0.000280885 | 0.022560489 |
| Tmem27 | 153 | 26 | 121 | 20 | 10 | 9 | 8 | 52.35 | 3.30 | 2.52E-05 | 0.004000671 |
| Mfrp | 4425 | 336 | 2760 | 402 | 160 | 231 | 244 | 1256.64 | 3.35 | 0.000573382 | 0.034180412 |
| Tmem72 | 3447 | 483 | 2864 | 274 | 117 | 198 | 216 | 1154.35 | 3.50 | 0.000293839 | 0.023194012 |
| Clec18a | 309 | 67 | 125 | 50 | 18 | 9 | 11 | 85.42 | 3.53 | 5.85E-08 | 2.62E-05 |
| Steap1 | 1654 | 169 | 1083 | 122 | 62 | 64 | 82 | 480.74 | 3.59 | 0.000217087 | 0.018823291 |
| Smim22 | 171 | 22 | 72 | 13 | 8 | 6 | 4 | 42.88 | 3.61 | 3.70E-05 | 0.005254543 |
| F5 | 6002 | 864 | 5267 | 576 | 250 | 317 | 302 | 2069.62 | 3.63 | 9.11E-05 | 0.010320779 |
| Akr1c18 | 80 | 44 | 2 | 53 | 6 | 1 | 4 | 26.14 | 3.63 | 0.000306518 | 0.023704429 |
| Otx2 | 3302 | 419 | 2262 | 434 | 81 | 165 | 181 | 1015.52 | 3.64 | 3.06E-05 | 0.004602063 |
| Sult1c2 | 179 | 12 | 54 | 14 | 7 | 2 | 7 | 38.37 | 3.66 | 0.000127758 | 0.013223203 |
| Col8a1 | 6998 | 545 | 3744 | 463 | 199 | 256 | 291 | 1822.94 | 3.68 | 0.000230768 | 0.019637585 |
| E230013L22Rik | 103 | 16 | 67 | 6 | 5 | 2 | 5 | 30.64 | 3.75 | 0.000142101 | 0.014270352 |
| Slco1a5 | 684 | 91 | 434 | 76 | 20 | 24 | 30 | 201.47 | 3.84 | 1.65E-08 | 8.78E-06 |
| 1500015O10Rik | 15538 | 995 | 7128 | 742 | 336 | 436 | 491 | 3704.40 | 3.95 | 0.000191637 | 0.017270735 |
| Gm10259 | 21 | 20 | 205 | 69 | 7 | 5 | 5 | 53.75 | 4.05 | 8.32E-06 | 0.001830857 |
| Apoo-ps | 33 | 69 | 58 | 24 | 3 | 4 | 3 | 32.31 | 4.08 | 4.83E-07 | 0.000162598 |
| RP23-131O4.2 | 245 | 37 | 154 | 25 | 7 | 10 | 5 | 72.00 | 4.09 | 3.35E-07 | 0.000123721 |
| 1110059M19Rik | 2082 | 274 | 1373 | 249 | 56 | 66 | 57 | 618.05 | 4.18 | 1.03E-06 | 0.000323626 |
| Kcnj13 | 7916 | 1078 | 6879 | 559 | 169 | 282 | 291 | 2628.46 | 4.24 | 2.07E-05 | 0.003539649 |
| Defb9 | 56 | 2 | 31 | 6 | 1 | 1 | 2 | 14.28 | 4.26 | 0.000891753 | 0.045666922 |
| Eci3 | 182 | 11 | 94 | 25 | 4 | 3 | 6 | 46.63 | 4.27 | 2.55E-06 | 0.000679015 |
| 2900093L17Rik | 283 | 36 | 178 | 36 | 7 | 3 | 13 | 82.12 | 4.28 | 9.80E-08 | 3.94E-05 |
| Folr1 | 13626 | 816 | 5761 | 513 | 196 | 306 | 290 | 3090.69 | 4.38 | 7.24E-05 | 0.008766237 |
| Gm16090 | 217 | 13 | 97 | 12 | 0 | 4 | 8 | 50.44 | 4.53 | 3.63E-05 | 0.005254543 |
| Stra8 | 7 | 86 | 108 | 15 | 1 | 5 | 3 | 40.51 | 4.58 | 3.08E-05 | 0.004602063 |
| C920025E04Rik | 78 | 4 | 11 | 17 | 2 | 1 | 0 | 14.80 | 4.70 | 0.000333285 | 0.024914892 |
| Sostdc1 | 3183 | 244 | 1993 | 205 | 59 | 58 | 54 | 858.45 | 4.74 | 4.01E-06 | 0.000976372 |
| Slc4a5 | 3030 | 215 | 2249 | 202 | 34 | 83 | 51 | 879.83 | 4.80 | 1.08E-05 | 0.002174952 |
| Aqp1 | 7203 | 501 | 4363 | 420 | 98 | 143 | 120 | 1897.31 | 4.81 | 4.88E-06 | 0.001150707 |
| Dpep1 | 385 | 19 | 256 | 7 | 6 | 4 | 9 | 102.35 | 4.88 | 0.000240599 | 0.02005545 |
| Mroh4 | 20 | 23 | 14 | 13 | 2 | 0 | 0 | 11.27 | 4.89 | 0.000182877 | 0.016612039 |
| Wfdc2 | 581 | 48 | 450 | 27 | 6 | 10 | 11 | 171.50 | 5.11 | 8.70E-06 | 0.001896428 |
| Cldn3 | 173 | 15 | 76 | 6 | 0 | 1 | 5 | 40.03 | 5.23 | 1.44E-05 | 0.002667299 |
| Defb11 | 1012 | 78 | 521 | 56 | 3 | 10 | 23 | 248.67 | 5.27 | 6.98E-06 | 0.001550376 |
| Olfr570 | 164 | 21 | 180 | 11 | 2 | 2 | 4 | 59.99 | 5.36 | 1.81E-07 | 7.15E-05 |
| Kcne2 | 4596 | 372 | 2998 | 225 | 46 | 56 | 53 | 1245.44 | 5.44 | 3.67E-07 | 0.0001294 |
| Cldn2 | 5738 | 415 | 3515 | 270 | 31 | 62 | 74 | 1498.26 | 5.62 | 3.57E-07 | 0.000127546 |
| Olfr1507 | 132 | 9 | 87 | 13 | 2 | 0 | 2 | 36.21 | 5.64 | 2.25E-06 | 0.00062705 |
| Ttr | 1336646 | 100690 | 804074 | 57307 | 8459 | 11228 | 11018 | 345461.40 | 5.94 | 2.57E-12 | 2.80E-09 |
| Tcf21 | 45 | 8 | 35 | 0 | 0 | 0 | 1 | 13.78 | 6.13 | 0.000827714 | 0.043634697 |
| Rprl3 | 97 | 48 | 23 | 87 | 0 | 0 | 2 | 35.23 | 6.61 | 1.27E-08 | 6.92E-06 |
| Gm10698 | 10 | 2001 | 888 | 8 | 6 | 7 | 17 | 564.79 | 6.73 | 0.000137446 | 0.013921603 |
| Psmb5-ps | 17 | 8 | 16 | 56 | 0 | 0 | 0 | 12.50 | 6.97 | 1.61E-06 | 0.000478113 |
| Gm5828 | 20 | 27 | 27 | 31 | 0 | 0 | 0 | 16.04 | 7.31 | 6.81E-08 | 2.90E-05 |
| Gm10184 | 2136 | 429 | 3049 | 2645 | 18 | 9 | 9 | 1193.72 | 7.49 | 8.79E-26 | 4.02E-22 |
