# Supplementary material for: Parkinson’s disease-related Leucine-rich repeat kinase 2 modulates nuclear morphology and genomic stability in striatal projection neurons during aging
Source: Mol Neurodegener. 2020 Feb 19;15:12. doi: 10.1186/s13024-020-00360-0 (PMC7031993; doi:10.1186/s13024-020-00360-0)
Supplement: Supplementary file 2 — Additional file 2: Table S2. Differentially expressed genes with adjust p < 0.05 in 12-month-old Lrrk2+/+ and Lrrk2−/− mice. [file 13024_2020_360_MOESM2_ESM.docx]

**Supplementary Table 2 Differentially expressed genes with adjust *p* <0.05 between 12-month-old *Lrrk2*^+/+^ and *Lrrk2*^-/-^ mice**

| **Gene_ID** | **-/-**  **#1** | **-/-**  **#2** | **-/-**  **#3** | **+/+**  **#1** | **+/+**  **#2** | **+/+**  **#3** | **Base**  **Mean** | **log2FoldChange** | **p-value** | **p-adj** |
| --- | --- | --- | --- | --- | --- | --- | --- | --- | --- | --- |
| Rpl3-ps1 | 23146 | 12295 | 9697 | 145 | 125 | 80 | 8137.93 | 7.27 | 9.82E-84 | 1.92E-79 |
| Lrrk2 | 1510 | 1301 | 1042 | 8414 | 8354 | 6212 | 4194.74 | -2.33 | 6.31E-69 | 6.16E-65 |
| Gm10600 | 11 | 10 | 8 | 761 | 806 | 1004 | 408.86 | -6.27 | 1.62E-68 | 1.05E-64 |
| Gm10184 | 522 | 455 | 740 | 7 | 16 | 13 | 330.36 | 5.86 | 7.49E-52 | 3.66E-48 |
| Herc3 | 1167 | 1220 | 923 | 5856 | 3456 | 4107 | 2607.74 | -1.75 | 4.74E-50 | 1.85E-46 |
| AA465934 | 875 | 888 | 394 | 81 | 78 | 57 | 412.22 | 3.54 | 1.35E-48 | 4.39E-45 |
| Gm5859 | 26 | 21 | 11 | 456 | 399 | 432 | 208.05 | -4.25 | 2.11E-48 | 5.14E-45 |
| Gm8822 | 369 | 627 | 253 | 17 | 4 | 10 | 223.92 | 5.58 | 1.97E-48 | 5.14E-45 |
| Gm13301 | 131 | 110 | 82 | 756 | 817 | 631 | 396.35 | -2.54 | 2.56E-36 | 5.56E-33 |
| Ppp1r3e | 113 | 144 | 89 | 668 | 620 | 543 | 341.54 | -2.17 | 5.54E-33 | 1.08E-29 |
| Ide | 4105 | 4621 | 3467 | 3168 | 2143 | 2118 | 3319.44 | 0.99 | 1.70E-31 | 3.01E-28 |
| Gm17167 | 289 | 285 | 247 | 1154 | 1021 | 921 | 621.12 | -1.66 | 2.65E-31 | 4.32E-28 |
| Cd59a | 483 | 669 | 417 | 3151 | 1971 | 1405 | 1237.27 | -1.76 | 3.35E-26 | 5.03E-23 |
| Gm21541 | 1 | 2 | 2 | 233 | 303 | 513 | 170.44 | -7.54 | 8.68E-24 | 1.21E-20 |
| 2610017I09Rik | 1574 | 1518 | 1230 | 3891 | 3323 | 3086 | 2350.72 | -1.00 | 1.40E-21 | 1.82E-18 |
| Tmem254a | 225 | 273 | 215 | 1302 | 684 | 1018 | 581.14 | -1.80 | 5.71E-21 | 6.97E-18 |
| A930018M24Rik | 173 | 266 | 161 | 77 | 74 | 47 | 137.49 | 1.85 | 3.56E-12 | 4.09E-09 |
| Gm9780 | 27 | 95 | 27 | 569 | 234 | 323 | 190.73 | -2.67 | 5.79E-12 | 6.28E-09 |
| 4930466F19RIK | 0 | 3 | 2 | 70 | 105 | 71 | 39.29 | -5.40 | 1.24E-11 | 1.27E-08 |
| Homez | 420 | 389 | 389 | 1356 | 919 | 752 | 668.96 | -1.04 | 1.93E-11 | 1.89E-08 |
| Gm13304 | 1 | 2 | 2 | 230 | 28 | 108 | 52.66 | -5.83 | 2.05E-11 | 1.91E-08 |
| Ccnb1ip1 | 69 | 51 | 49 | 6 | 2 | 0 | 31.84 | 4.73 | 3.68E-11 | 3.27E-08 |
| Gm13302 | 3 | 1 | 5 | 53 | 118 | 92 | 43.97 | -4.65 | 5.40E-11 | 4.59E-08 |
| Slc39a2 | 207 | 220 | 97 | 657 | 621 | 418 | 348.21 | -1.47 | 8.70E-11 | 7.08E-08 |
| Kpna2 | 438 | 442 | 178 | 1612 | 878 | 879 | 681.48 | -1.42 | 2.88E-10 | 2.25E-07 |
| Pnp2 | 95 | 56 | 60 | 351 | 238 | 211 | 157.69 | -1.64 | 3.99E-10 | 3.00E-07 |
| Tmem260 | 892 | 890 | 739 | 2087 | 1646 | 1273 | 1207.92 | -0.71 | 5.05E-10 | 3.65E-07 |
| Col5a2 | 242 | 287 | 236 | 819 | 727 | 459 | 439.64 | -1.11 | 8.44E-10 | 5.89E-07 |
| Gm10557 | 107 | 92 | 133 | 511 | 402 | 297 | 243.08 | -1.55 | 9.61E-10 | 6.47E-07 |
| Klhl33 | 367 | 406 | 260 | 187 | 200 | 149 | 267.24 | 1.18 | 1.95E-09 | 1.27E-06 |
| 2610005L07Rik | 2514 | 2322 | 1650 | 4997 | 3864 | 3167 | 2970.71 | -0.63 | 2.22E-09 | 1.40E-06 |
| Gm21093 | 709 | 839 | 605 | 1572 | 1388 | 1366 | 1050.83 | -0.77 | 2.60E-09 | 1.59E-06 |
| Sycp1 | 3 | 2 | 0 | 97 | 78 | 28 | 30.37 | -5.06 | 2.96E-09 | 1.75E-06 |
| Pnpla3 | 177 | 220 | 153 | 697 | 349 | 454 | 321.92 | -1.17 | 4.46E-09 | 2.56E-06 |
| Gm26782 | 173 | 216 | 135 | 603 | 352 | 363 | 289.52 | -1.06 | 4.91E-09 | 2.74E-06 |
| Fgfbp3 | 349 | 452 | 292 | 272 | 221 | 167 | 295.43 | 0.99 | 1.27E-08 | 6.90E-06 |
| Gm17081 | 3 | 4 | 6 | 46 | 73 | 72 | 32.87 | -3.66 | 1.42E-08 | 7.49E-06 |
| Ddhd1 | 2542 | 2929 | 2054 | 5615 | 3981 | 3343 | 3286.39 | -0.51 | 1.64E-08 | 8.45E-06 |
| G530011O06Rik | 313 | 435 | 507 | 196 | 150 | 190 | 316.67 | 1.51 | 1.81E-08 | 9.05E-06 |
| A730060N03Rik | 349 | 623 | 458 | 216 | 238 | 213 | 363.27 | 1.34 | 2.37E-08 | 1.16E-05 |
| Sgcg | 58 | 91 | 73 | 14 | 8 | 20 | 47.11 | 2.64 | 3.26E-08 | 1.55E-05 |
| Wnk4 | 967 | 875 | 567 | 579 | 552 | 450 | 671.99 | 0.84 | 1.47E-07 | 6.84E-05 |
| Btaf1 | 3401 | 3071 | 2519 | 3157 | 2361 | 1890 | 2736.30 | 0.56 | 1.66E-07 | 7.52E-05 |
| CR974586.1 | 56 | 51 | 27 | 235 | 111 | 181 | 102.72 | -1.73 | 1.90E-07 | 8.42E-05 |
| Mroh7 | 607 | 457 | 428 | 1174 | 1054 | 921 | 750.96 | -0.82 | 2.86E-07 | 0.000124231 |
| 6820431F20Rik | 2419 | 2553 | 1990 | 5175 | 4063 | 2985 | 3087.16 | -0.53 | 4.76E-07 | 0.00020194 |
| Cdh4 | 1445 | 1595 | 1154 | 1386 | 1168 | 899 | 1276.93 | 0.54 | 5.75E-07 | 0.000238797 |
| Ang | 4 | 24 | 10 | 89 | 93 | 83 | 47.57 | -2.59 | 8.95E-07 | 0.000364066 |
| Gm10698 | 6 | 27 | 6 | 97 | 476 | 370 | 162.54 | -4.54 | 2.40E-06 | 0.000955683 |
| Gm16286 | 3567 | 3843 | 2740 | 6320 | 4698 | 4553 | 4178.66 | -0.36 | 3.14E-06 | 0.00122456 |
| Lynx1 | 11947 | 11441 | 7691 | 21771 | 14605 | 16806 | 13607.97 | -0.53 | 3.31E-06 | 0.001241435 |
| Myh6 | 326 | 373 | 365 | 242 | 226 | 202 | 298.17 | 0.93 | 3.37E-06 | 0.001241435 |
| Rpgrip1 | 88 | 105 | 135 | 49 | 30 | 43 | 80.02 | 1.73 | 3.37E-06 | 0.001241435 |
| Gm20625 | 94 | 72 | 47 | 36 | 13 | 10 | 46.31 | 2.17 | 3.82E-06 | 0.001382617 |
| Myo1b | 4199 | 4536 | 3988 | 4445 | 3589 | 2650 | 3922.48 | 0.54 | 4.13E-06 | 0.001464899 |
| Csmd1 | 3590 | 3640 | 2892 | 3411 | 2922 | 2617 | 3194.08 | 0.43 | 5.34E-06 | 0.001861783 |
| B230217O12Rik | 100 | 222 | 116 | 410 | 355 | 401 | 257.63 | -1.20 | 7.76E-06 | 0.002660068 |
| Sgpp2 | 1386 | 1584 | 1059 | 2631 | 2070 | 1759 | 1696.49 | -0.42 | 8.11E-06 | 0.002730513 |
| Sgk1 | 3871 | 3172 | 3263 | 8620 | 5200 | 5368 | 4744.18 | -0.60 | 1.07E-05 | 0.003555715 |
| B3galt2 | 508 | 550 | 375 | 1475 | 723 | 740 | 687.03 | -0.74 | 1.30E-05 | 0.004171213 |
| Gm11632 | 9 | 33 | 37 | 1 | 1 | 0 | 15.20 | 5.64 | 1.29E-05 | 0.004171213 |
| Dhtkd1 | 574 | 753 | 652 | 541 | 508 | 337 | 571.08 | 0.79 | 1.36E-05 | 0.004291018 |
| Apobec3 | 163 | 207 | 138 | 407 | 311 | 317 | 248.33 | -0.78 | 1.45E-05 | 0.00449649 |
| Tmem254c | 158 | 170 | 90 | 563 | 327 | 212 | 233.83 | -1.11 | 1.64E-05 | 0.004988848 |
| E230025N22Rik | 406 | 403 | 315 | 253 | 281 | 219 | 319.28 | 0.82 | 1.72E-05 | 0.005096508 |
| Entpd4 | 4653 | 6655 | 5117 | 11953 | 8768 | 7947 | 7291.04 | -0.53 | 1.70E-05 | 0.005096508 |
| Gm10132 | 4 | 46 | 9 | 0 | 0 | 0 | 10.02 | 6.90 | 1.76E-05 | 0.005132747 |
| Cnih3 | 592 | 873 | 591 | 1663 | 1016 | 1431 | 994.64 | -0.75 | 2.40E-05 | 0.006891001 |
| Kcnj3 | 1216 | 1572 | 1056 | 3876 | 1673 | 2317 | 1849.84 | -0.74 | 2.50E-05 | 0.00707049 |
| Gm2309 | 4 | 1 | 1 | 31 | 29 | 30 | 14.94 | -3.68 | 2.59E-05 | 0.007222178 |
| 4930480K23Rik | 657 | 507 | 360 | 441 | 350 | 281 | 434.29 | 0.76 | 2.70E-05 | 0.007414508 |
| Kcng4 | 149 | 100 | 128 | 886 | 211 | 302 | 265.51 | -1.50 | 3.32E-05 | 0.009012748 |
| Tmem117 | 637 | 636 | 512 | 666 | 321 | 343 | 518.22 | 0.73 | 3.41E-05 | 0.009114273 |
| Gm10125 | 614 | 693 | 521 | 1276 | 877 | 899 | 789.67 | -0.47 | 3.54E-05 | 0.009353937 |
| Nnt | 1020 | 1271 | 921 | 3090 | 1658 | 1428 | 1481.89 | -0.63 | 3.91E-05 | 0.010184794 |
| Cdk5rap1 | 276 | 411 | 276 | 697 | 565 | 642 | 465.46 | -0.75 | 4.35E-05 | 0.011182896 |
| Abcc6 | 152 | 140 | 78 | 74 | 63 | 25 | 89.47 | 1.46 | 4.91E-05 | 0.012455841 |
| Nub1 | 5315 | 6122 | 4791 | 6363 | 4810 | 4316 | 5274.61 | 0.34 | 5.19E-05 | 0.012999833 |
| Ryr1 | 4850 | 4910 | 3606 | 2873 | 3862 | 2821 | 3896.50 | 0.70 | 5.27E-05 | 0.01301843 |
| St18 | 854 | 642 | 521 | 1667 | 1476 | 898 | 968.36 | -0.73 | 5.57E-05 | 0.013584527 |
| Hes5 | 1041 | 724 | 637 | 721 | 522 | 543 | 704.75 | 0.69 | 6.05E-05 | 0.014583869 |
| Sox18 | 417 | 494 | 333 | 346 | 332 | 260 | 366.63 | 0.65 | 6.68E-05 | 0.01590816 |
| Mesp2 | 96 | 85 | 54 | 51 | 26 | 28 | 57.43 | 1.44 | 6.88E-05 | 0.016176319 |
| Duxbl2 | 4 | 28 | 10 | 96 | 115 | 44 | 45.70 | -2.37 | 7.49E-05 | 0.017421377 |
| Plac9b | 9 | 28 | 32 | 125 | 100 | 81 | 59.02 | -1.84 | 8.42E-05 | 0.019340437 |
| Peli2 | 2273 | 2666 | 1961 | 2359 | 2106 | 1740 | 2189.50 | 0.41 | 0.000110765 | 0.025151311 |
| Gm21092 | 3908 | 4101 | 3380 | 8878 | 6546 | 4373 | 4999.17 | -0.50 | 0.000121687 | 0.027313855 |
| Ppwd1 | 507 | 646 | 367 | 514 | 379 | 302 | 449.50 | 0.61 | 0.000132527 | 0.029408903 |
| Gm13306 | 48 | 79 | 49 | 153 | 138 | 188 | 106.37 | -1.23 | 0.000139122 | 0.030525526 |
| Sbsn | 1342 | 1321 | 1047 | 1216 | 1045 | 991 | 1167.57 | 0.44 | 0.000142708 | 0.030964363 |
| Gm13086 | 106 | 125 | 106 | 643 | 116 | 450 | 238.72 | -1.53 | 0.000154638 | 0.033184331 |
| Gm20938 | 223 | 370 | 198 | 733 | 379 | 682 | 413.71 | -0.95 | 0.000161036 | 0.034181682 |
| Aldh1a1 | 1518 | 1853 | 1131 | 3726 | 2189 | 1874 | 1950.74 | -0.51 | 0.000187008 | 0.039267612 |
| Baz1a | 932 | 615 | 504 | 559 | 529 | 344 | 585.42 | 0.78 | 0.000193954 | 0.039868825 |
| Tox4 | 2950 | 2814 | 2336 | 5263 | 3649 | 3481 | 3327.59 | -0.34 | 0.000192115 | 0.039868825 |
| Tlk2 | 2607 | 2860 | 2002 | 3079 | 2152 | 2023 | 2432.51 | 0.31 | 0.000208879 | 0.042489376 |
| Parp2 | 1115 | 1183 | 864 | 1206 | 934 | 732 | 1000.64 | 0.41 | 0.000223171 | 0.044928737 |
| Gm21967 | 690 | 973 | 693 | 751 | 415 | 602 | 692.82 | 0.68 | 0.00023812 | 0.047449144 |
| 4632419I22Rik | 452 | 566 | 427 | 1169 | 761 | 627 | 640.26 | -0.53 | 0.000241145 | 0.04756645 |
