# Supplementary figures and images for: Parkinson’s disease-related Leucine-rich repeat kinase 2 modulates nuclear morphology and genomic stability in striatal projection neurons during aging
Source: Mol Neurodegener. 2020 Feb 19;15:12. doi: 10.1186/s13024-020-00360-0 (PMC7031993; doi:10.1186/s13024-020-00360-0)

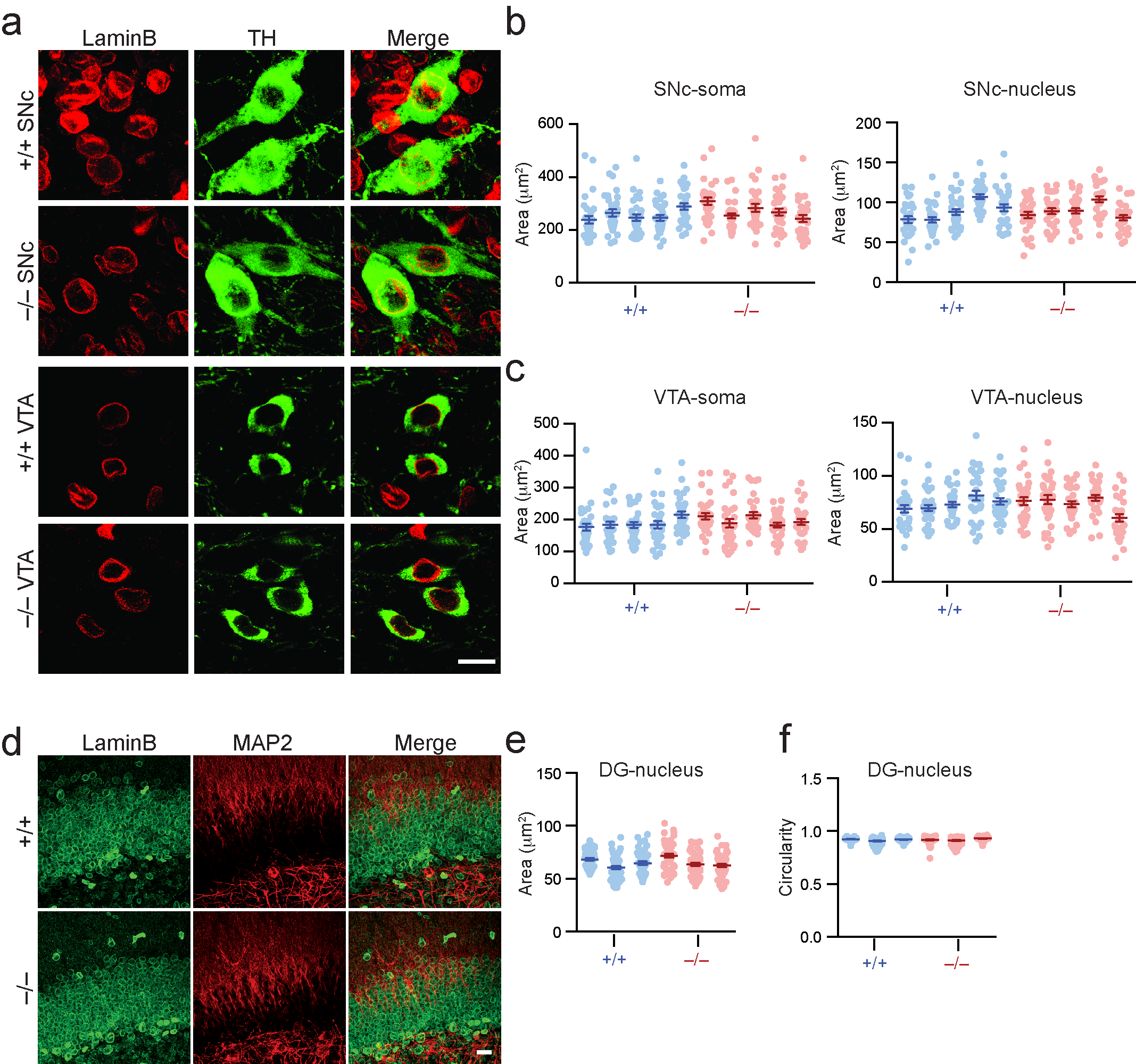

Supplement: Supplementary file 3 — Additional file 3: Figure S1. No alteration of nuclear size in midbrain dopaminergic neurons and hippocampal neurons of 12-month-old Lrrk2−/− mice. a-c Co-staining of Lamin B and TH in SNc and VTA neurons of 12-month-old Lrrk2+/+ and Lrrk2−/− mice (a). Scale bar, 20 μm. The areas of soma and nuclei in SNc neurons were measured from five 12-month-old Lrrk2+/+ and Lrrk2−/− mice (b). N = 5 mice per genotype, about 30 neurons counted per animal. Conditional logistic regression test, no statistically significant difference was identified. The areas of soma and nuclei in VTA neurons were measured from five 12-month-old Lrrk2+/+ and Lrrk2−/− mice (c). N = 5 mice per genotype, about 30 neurons counted per animal. Conditional logistic regression test, no statistically significant difference was identified. d-f Co-staining of Lamin B and MAP2 in hippocampal dentate gyrus (DG) neurons of 12-month-old Lrrk2+/+ and Lrrk2−/− mice (d). Scale bar, 20 μm. The area of nuclei in DG neurons was measured from three 12-month-old Lrrk2+/+ and Lrrk2−/− mice e). N = 3 mice per genotype, about 50 neurons counted per animal. Conditional logistic regression test, no statistically significant difference was identified. The circularity of nuclei in DG neurons was measured from three 12-month-old Lrrk2+/+ and Lrrk2−/− mice (f). N = 3 mice per genotype, about 50 neurons counted per animal. Conditional logistic regression test, no statistically significant difference was identified. [file 13024_2020_360_MOESM3_ESM.tif]

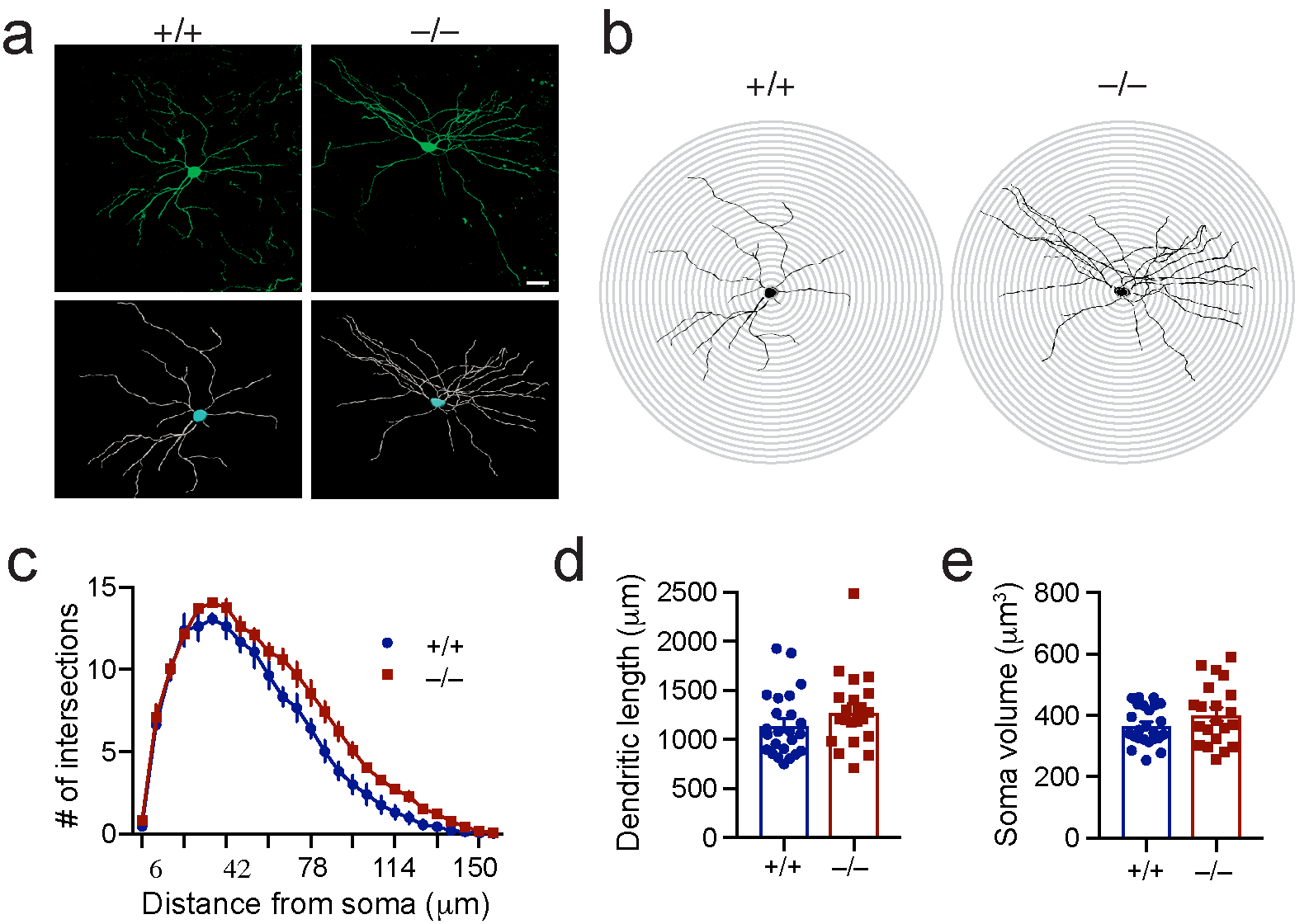

Supplement: Supplementary file 4 — Additional file 4: Figure S2. No alteration of dendritic complexity in the SPNs of 3-month-old Lrrk2−/− mice. a GFP-labeled SPNs (top panel). 3D reconstruction of the top fluorescent image (bottom panel). Scale bar, 20 μm. b, c Sholl analysis of dendritic complexity GFP-labeled SPNs. N = 5 mice per genotype, 5–9 neurons per animal. Benjamin-Hochberg multiple comparison test, no statistically significant difference was identified. d Dendritic length of GFP-labeled SPNs. N = 5 mice per genotype, 5–9 neurons per animal. Unpaired t-test, p = 0.195. e Soma volume of GFP-labeled SPNs. N = 5 mice per genotype, 5–9 neurons per animal. Unpaired t-test, p = 0.151. [file 13024_2020_360_MOESM4_ESM.tif]

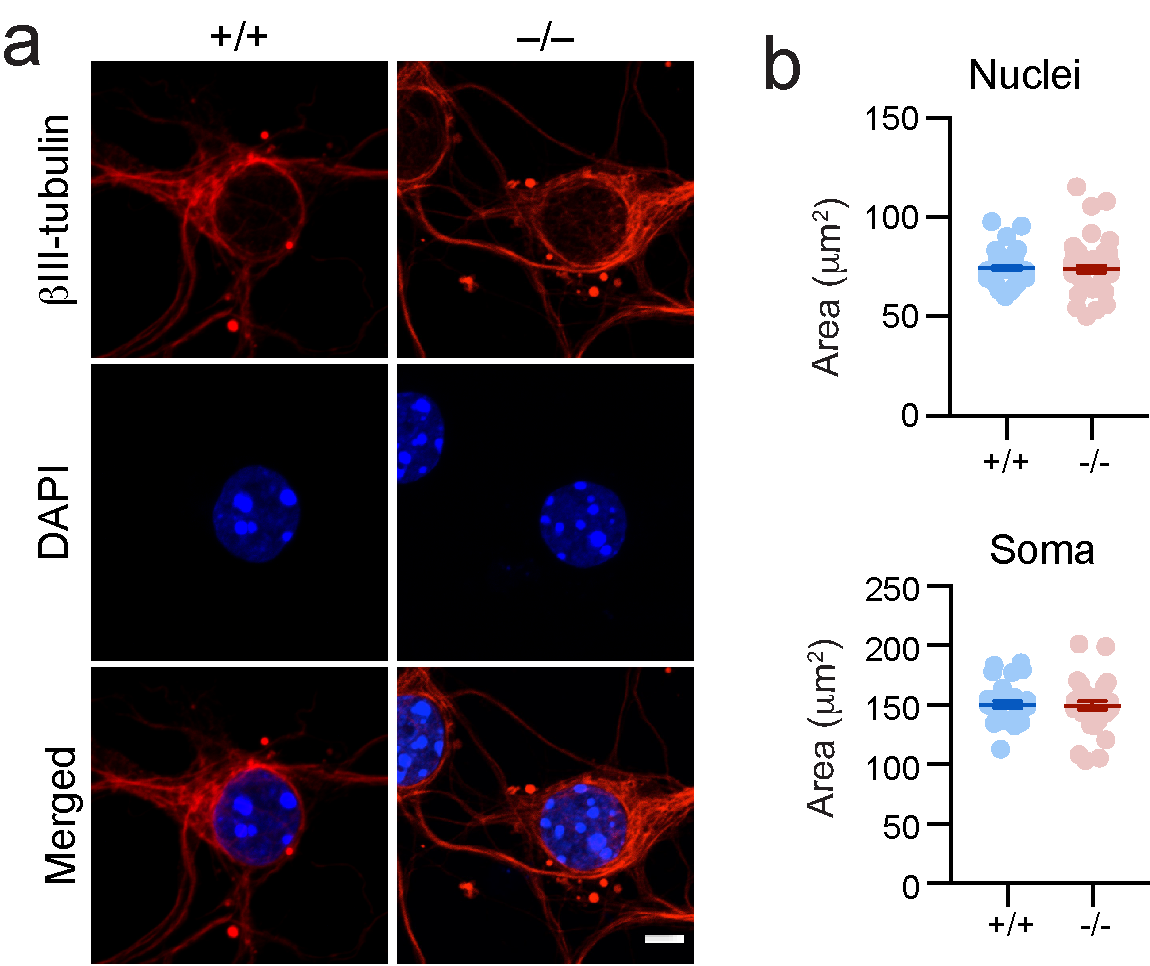

Supplement: Supplementary file 5 — Additional file 5: Figure S3. No alteration of nuclear and soma size of the Lrrk2−/− SPNs after 2 weeks in culture. a Co-staining of βIII-tubulin and DAPI of the cultured SPNs. Scale bar, 20 μm. b Unpaired t-test, n = 50 neurons per genotype. [file 13024_2020_360_MOESM5_ESM.tif]
